# Mitochondrial proteome of *Acanthamoeba* delineates aerobic and anaerobic pathways dynamically regulated by oxygen

**DOI:** 10.1016/j.cell.2026.08.056

**Published:** 2026-10-01

**Authors:** Jonathan A. Stefely, Felicia G. Deng, Michael Z. Chen, Khanh Nguyen, Bridget E. Luce, Michelle Y. Fry, Xiaoyan A. Guo, Konstantinos Maliaris, Keith D. Rivera, Malvina Papanastasiou, Tiana M. Issa, Bharath Kanakapura Sundararaj, Sophia LY. Root, DR Mani, MitoCarta Tree of Life Consortium, Luke H. Chao, Sarah E. Calvo, Namrata D. Udeshi, John Samuelson, Vamsi K. Mootha

**Affiliations:** 1 Department of Molecular Biology, Massachusetts General Hospital, Boston, MA 02114, USA; 2 Harvard Medical School, Boston, MA 02115, USA; 3 Broad Institute of MIT and Harvard, Cambridge, MA 02142, USA; 4 Howard Hughes Medical Institute, Massachusetts General Hospital, Boston, MA 02114, USA; 5 Department of Molecular and Cell Biology, Boston University Goldman School of Dental Medicine, Boston, MA 02118, USA; 6 Lead contact

**Keywords:** HydA, HydE, HydF, HydG, PFOR, HCP, NirK, NDH2, HupA, Abf2, TFAM, SDHB, MDH, HGT, BN-PAGE-MS, mitochondrial ribosome, hybrid cluster protein, complex I, ATP synthase, mitochondria, *Acanthamoeba*, proteome, complexome, protozoa, horizontal gene transfer, electron transport chain, oxidative phosphorylation, anaerobic metabolism, hydrogenase

## Abstract

*Acanthamoeba castellanii* causes infectious blindness and is a key evolutionary outgroup to humans and fungi. Its divergent mitochondria are of outstanding interest due to predicted aerobic and anaerobic functions with potential for drug targeting. However, a detailed delineation of its bioenergetic machinery, their activities, and their regulation remains lacking. Here, we integrate mitochondrial immunoprecipitation, density gradient purification, mass spectrometry, protein correlation profiling, and microscopy to generate a high-confidence inventory of the *Acanthamoeba* mitoproteome. The resulting AcMitoCarta contains 1,122 proteins, including 381 lacking orthologs in human and yeast mitochondria. Complexome analysis highlights 20 macromolecular assemblies. Complementary proteomic and transcriptomic profiling reveals extensive rewiring of the organelle’s bioenergetic machinery by oxygen, including induction of an anaerobic pyruvate:ferredoxin oxidoreductase-to-hydrogenase pathway under anoxia. We further demonstrate that *Acanthamoeba* can produce H_2_ gas under anoxic conditions, via a mitochondria-localized, oxygen-labile hydrogenase. AcMitoCarta establishes a framework for dissecting the interplay between aerobic and anaerobic energy metabolism.

## In brief

The complete mitochondrial proteome of Acanthamoeba castellanii—an infectious amoeba that causes blindness – catalogs 1,122 proteins, with over a third with no human or yeast counterpart, including potential drug candidates. Oxygen levels rewire this organism’s bioenergetic machinery, switching on an anaerobic pathway that produces H2 gas via a mitochondrial hydrogenase.

## Graphical Abstract

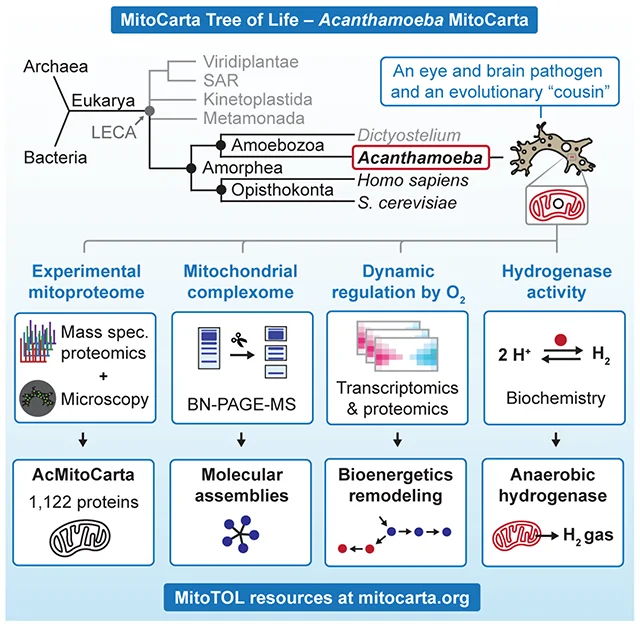

## INTRODUCTION

Mitochondria are best known for powering aerobic metabolism. Decades of studies, primarily in animals and yeast, revealed how a small number of proteins encoded by the mitochondrial genome (mtDNA) partner with many proteins encoded by the nuclear genome. Together, they assemble the machinery of the electron transport chain (ETC) and oxidative phosphorylation (OXPHOS)—enabling production of ATP using redox energy.^1^ With a few notable differences (*e.g.*, lack of complex I in *Saccharomyces*), human and yeast mitochondria are very similar.^2^

However, across the broader tree of life, mitochondria have diversified dramatically.^3,4^ In certain lineages, reductive evolution led to the complete loss of mtDNA and OXPHOS, as in *Trichomonas vaginalis*, whose mitochondrion-related hydrogenosome^5^ generates ATP via anaerobic pathways. In others, mitochondria have gained new capabilities, such as the acquisition of rhodoquinone biosynthesis via horizontal gene transfer (HGT)^6^ to enable fumarate-based respiration in *Ascaris*.^7^ There is ongoing debate as to whether the original endosymbiont and earliest mitochondria were strict aerobes, strict anaerobes, or facultative anaerobes.^8,9^ A better understanding of the diversity of mitochondrial energy metabolism can provide insight into the origins of the organelle and how environmental pressures have shaped its evolution.

Here, as part of our MitoCarta Tree of Life Consortium (MitoTOL), we selected *Acanthamoeba castellanii* for mitochondrial proteome characterization as a representative of Amoebozoa.^10^ *Acanthamoeba* is ideally positioned (Figure 1A) for comparative biology as an evolutionary “cousin” to the Opisthokonta clade containing humans and *Saccharomyces*—whose mitochondria are extensively investigated and for which high-quality mitoproteomes are available.^11–14^ *Acanthamoeba* is a globally distributed, protozoan pathogen that causes painful, infectious blindness and fatal brain abscesses.^15–22^ *Acanthamoeba* is also a model for oxygen biology as it must adapt to variable O_2_ levels ranging from near-anoxia in deep water to 21% O_2_ in air-exposed soil, and prior studies indicate capacity for switching between aerobic and anaerobic metabolism.^23–27^ In host interactions, *Acanthamoeba* is predicted to encounter hypoxia in tissue abscesses and in the cornea, especially during sleep or behind contact lenses, which are a risk factor for *Acanthamoeba* keratitis. Current drugs are toxic and not targeted,^28,29^ and eye surgery is frequently required.^30,31^ Comprehensive and accurate inventories of protozoan mitoproteomes, such as that of *Acanthamoeba*, promise to shed molecular insights into their bioenergetic capacities and motivate new therapies targeting these pathways.

Early studies pointed to fascinating features of *Acanthamoeba* mitochondria. Although it is a “cousin” of animals and fungi, its circular mtDNA encodes an impressive 42 open reading frames (ORFs)^32–35^ in comparison to 13 in humans^36^ and eight conserved mtDNA ORFs in yeast.^37^ *Acanthamoeba* contains a superset of mitochondrial pathways found in humans and yeast, including: (i) a full OXPHOS system spanning complex I–V,^33,35,38–40^ (ii) an ER-mitochondria encounter structure (ERMES)^41^, (iii) an uncoupling protein (UCP)^42^, (iv) three type II NADH dehydrogenases (NDH2s)^43^ (found in yeast but absent in humans), and (v) an alternative oxidase (AOX),^44,45^ which is absent in humans and in yeast. In addition, prior genome analysis suggested *Acanthamoeba* may possess pathways for anerobic ATP production not present in yeast or animals. These include [FeFe]-hydrogenase (HydA) and its assembly factors (HydE, HydF, HydG), pyruvate:ferredoxin oxidoreductase (PFOR), and acetate:succinate CoA transferase 1B (ASCT1B), which is important for anaerobic ATP production via substrate-level phosphorylation.^46^ While mitochondrial localization has been predicted,^46^ definitive subcellular localization of these enzymes, their assembly factors, and their activities is still lacking. Neither *Acanthamoeba* cells nor their isolated mitochondria have been shown to generate or consume H_2_. It also remains unclear whether these pathways and their activities are constitutively active or environmentally regulated.

Systematic and accurate characterization of the *Acanthamoeba* mitoproteome would help to definitively localize these enzymes and illuminate any missing pathway members, but this is particularly challenging for this organism for several reasons. First, nuclear genome annotation has been a longstanding challenge due to many introns and short intergenic distances (median of 41 nucleotides), making it difficult to determine whether an N-terminal mitochondrial targeting sequence (MTS) is present. Second, the organism is notorious for having been the recipient of extensive HGT,^47^ creating challenges for comparative genetics with other Amoebozoa such as *Dictyostelium*.^48^ Third, as an eye pathogen, special laboratory precautions are essential, and to date, not a single epitope-tagged mitochondrial protein has been localized using microscopy.

A key advance in *Acanthamoeba* mitoproteomics was a 2014 study by Gawryluk and colleagues^33^ who performed mass spectrometry (MS) proteomics on purified mitochondria. While no quality assessment was provided in that manuscript, we estimate that their experimental MS dataset has 61% sensitivity with a ~25% false discovery rate (FDR) (see Methods). Despite the lack of a high-quality genome assembly and annotation at that time, Gawryluk et al used domain expertise in mitochondrial biology to manually remove 165 contaminants from their experimental MS inventory and manually add 324 predictions based on sequence homology—yielding a final list of 1,033 proteins (709 with MS support, plus 324 predictions).^33^ While an important first step, it was heavily dependent on manual curation and relied on earlier-generation MS,^49^ both of which contribute to low sensitivity and specificity.

Here, we sought to overcome these challenges to create a high accuracy, experimentally defined mitoproteome and use this resource to investigate the flexibility of this organelle’s bioenergetics. We first re-annotated the entire *Acanthamoeba* nuclear genome using deep transcriptomics, improving the accuracy from 52% to 98% (Stefely et al^50^). We used the Neff strain because it provides the reference nuclear^51^ and mtDNA^32^ genomes for *Acanthamoeba castellanii* in VEuPathDB,^52^ NCBI GenBank, and UniProt. We then integrated mitochondrial purification with protein MS and protein correlation profiling to generate a high-quality mitoproteome. We augment this with epitope tagging and microscopy to arrive at AcMitoCarta—an inventory of 1,122 experimentally defined mitochondrial proteins. We report the complexome of *Acanthamoeba* mitochondria, explore how the mitoproteome remodels in anaerobic and aerobic environments, and demonstrate hydrogenase activity in anaerobically isolated mitochondria. Finally, we extend these findings to clinical isolates of *Acanthamoeba*.

## RESULTS

### Mass spectrometry-based MitoViaPCP inventory

After testing multiple strategies, we arrived at a uniform workflow across our MitoTOL project, leveraging: (i) serial density and affinity purification of mitochondria, (ii) data-independent acquisition (DIA) MS-based proteomics^53–55^ of samples spanning the purification steps, and (iii) data analysis using protein correlation profiling (PCP)^56^ to distinguish true mitochondrial proteins from contaminants.

To enable mitochondrial immunoprecipitation (MitoIP),^57^ we expressed 3xHA-eGFP on the cytosolic face of the mitochondrial outer membrane protein TOM7 using a genetically encoded “MitoIP” construct (3xHA-eGFP-TOM7) (Figures 1B and S1A). Mitochondria were isolated using magnetic anti-GFP microbeads, treated with proteinase K (PK) to digest peripheral contaminants, and purified with an isotonic Percoll density gradient (Figure 1C). Mitochondrial density increased after PK treatment (Figure 1C), which we attribute to removal of associated buoyant light membrane particles from other organelles. Both the outer and inner mitochondrial membranes remained intact (Figure 1C). Mitochondria of increasing purity were isolated throughout the MitoIP procedure and a parallel procedure with wild type (WT; untransfected) cells, as quantified by transmission electron microscopy (TEM) (Figure 1C) and streptavidin blotting for endogenously biotinylated mitochondrial carboxylases (Figure S1B).

The WT and MitoIP mitochondrial purifications were each conducted in biological triplicate and all fractions (24 total samples) were analyzed by MS-based proteomics (Figure S1C). Across all samples, we identified 9,579 proteins in the ACANB genome annotation^50^ (Figure 1D and Tables S1, S2). Proteins benchmarked as mitochondrial based on homology with human and yeast mitochondrial proteins increased in relative abundance throughout the procedure (Figure S1D).

Because no single fraction contains 100% pure mitochondria, we used PCP across all fractions to score proteins based on their profile similarity to a core set of mitochondrial proteins. To define this profile, we selected three mitochondrial marker proteins that are robustly expressed, highly conserved, and readily detected by MS: TOM40, localized to the outer membrane, and mitochondrial heat shock protein 60 (HSPD1) and iron-sulfur cluster assembly protein ISCU, localized to the matrix. Individual protein abundances were correlated with the average profile of the three markers to determine their PCP scores (Figure 1D). Those with a PCP score >0.6 defined a “MitoViaPCP” list, which has 1,084 proteins with an estimated sensitivity of 80% and FDR of 8% (Figure 1E and Table S2), on par with the human MitoCarta.^11^ In comparison to the MS-based list of Gawryluk and colleagues^33^ (FDR 25% at 61% sensitivity), we identified 496 proteins that are unique to MitoViaPCP, including the anaerobic hydrogenase HydA and two of its assembly factors, HydE and HydG (Figure S1E). Most of the 286 proteins detected via MS by Gawryluk et al and missing in the MitoViaPCP dataset were likely non-mitochondrial contaminants based on their PCP scores.

### Confocal microscopy

A major gap in prior studies has been the absence of confocal microscopy validation of any mitochondrial protein in *Acanthamoeba*. To stain endogenous mitochondria, we developed an anti-MICU1 antibody targeting a conserved mitochondrial protein involved in calcium uptake^58^ (MICU1). We also found the large mtDNA of *Acanthamoeba* to be visible using nuclear dyes such as Hoechst 33342 (Figure 1F).

We used gene synthesis based on our ACANB annotation^50^ to clone C-terminal HA-tagged constructs of 76 MitoViaPCP proteins (selected across the range of PCP scores) plus an additional 22 candidates scoring below our PCP threshold (primarily selected due to homology to known mitochondrial proteins) (Figures 1F, S1F, and S2). The microscopy validated 74/76 (97%) MitoViaPCP proteins, including two dual localized in mitochondria and another organelle (OsmC and Erv1). Two of the tagged MitoViaPCP proteins were observed in the cytosol (ACANB00049 and ACANB05560), although epitope tags can disrupt localization. In total, our microscopy localized 96 mitochondrial proteins (74 MitoViaPCP plus the 22 additional), including seven that are conserved in mitochondria of multiple pathogenic protists but absent in humans (ClpQ protease, Class-I fumarase, a PLD-type cardiolipin synthase, three NDH2s, and AOX)—as highlighted by our comparative MitoTOL study^59^.

### The *Acanthamoeba* MitoCarta

To generate a complete mitoproteome inventory, we combined the MS-based MitoViaPCP dataset (n = 1,084), the MitoViaMicroscopy set (n = 96), and mtDNA ORFs^32^ (n = 42) (Figure 1G). The latter are notoriously difficult to detect and quantify by MS because they tend to be small and hydrophobic (Figure S3A). We make this AcMitoCarta inventory (n = 1,122) freely available (mitocarta.org and Table S2).

We compared AcMitoCarta to the mitoproteome of Gawryluk and colleagues^33^ (n = 1,033, 69% experimental, 31% predictions) (Figure 2A). The 316 AcMitoCarta proteins absent from the Gawryluk list have strong PCP scores and high DeepLoc2.1^60^ mitochondrial scores, which provide orthogonal support (Figure 2B). The 316 proteins present only in AcMitoCarta also tend to have lower abundance (Figure 2C), providing a rationale for why they were previously missed. Conversely, the 227 proteins exclusive to the Gawryluk list tend to have low or negative PCP scores (Figure 2A), and low DeepLoc2.1 mito scores (Figure 2B), indicative of non-mitochondrial proteins. Notably, for the 806 overlapping proteins (Figure 2A), the prior list had experimental support for only 591 (73%).

Compared to the entire proteome, AcMitoCarta proteins tend to have lower molecular weights (MWs), higher isoelectric points, and higher abundance (Figure S3B). As expected, they are also enriched for proteins of prokaryotic lineage (Figure 2D). Only 46% (514/1,122) of AcMitoCarta proteins have a predicted MTS by TargetP2.0^61^ (highlighting the need to experimentally define a mitoproteome), compared to 1% (124/14,822) of non-AcMitoCarta proteins (*P* < 0.0001 by Chi-square) (Figure S3C).

Based on OrthoGroups (OGs) (Table S3)^59^, 741 AcMitoCarta proteins are orthologous to known human^11^ or yeast^14^ mitochondrial proteins, but 381 AcMitoCarta proteins are not (Figure 2E). Of these “unique” AcMitoCarta proteins, 168 have no known Pfam domain^62^ and 300 have no bidirectional best hit (BBH) in *Dictyostelium discoideum*.^63^ The 316 AcMitoCarta-only proteins (Figure 2A) include homologs of conserved proteins such as the intermediate peptidase MIPEP (Oct1)^64,65^ but are also enriched for proteins unique to *Acanthamoeba* (Figure 2F). Expanding our AcMitoCarta orthogroup analysis to the entire genomes of 203 eukaryotic species and 34 prokaryotic clades (Figure 2G), we find 70% have homologs in prokaryotes, 19% appear to have emerged in eukaryotes, and 11% are lineage-specific, most of which are not found in other Amoebozoa genomes.

Beginning with human “MitoPathways”,^13^ we determined whether *Acanthamoeba* has mitochondrial or non-mitochondrial orthologs (Table S3). Some pathways are highly conserved and have ancient prokaryotic origins (*e.g.*, CoQ biosynthesis^66^), while other pathways such as OXPHOS have a mix of prokaryotic origins and more recent eukaryotic ancestry (Figure 2G). Human CI, CIV, and CV each contain multiple subunits that lack *Acanthamoeba* orthologs, which indicates either metazoan innovations or marked sequence divergence. “Central dogma” pathways such as mtDNA maintenance and the mitochondrial ribosome also show marked divergence, with multiple human members lacking detectable orthologs in *Acanthamoeba* (Figure 2H). For example, *Acanthamoeba* lacks a typical mitochondrial ortholog of the canonical mtDNA-binding proteins TFAM^67,68^ or Abf2.^69,70^

### Horizontal gene transfer shapes the *Acanthamoeba* mitoproteome

The *Acanthamoeba* genome sequence revealed many genes derived from prokaryotes via HGT,^47^ which we confirmed in our ACANB annotation^50^. We predict that 53 (5%) AcMitoCarta proteins are products of relatively recent HGT from prokaryotes (Figure 2I and Table S2), accounting for 13% of prokaryote-derived HGTs in *Acanthamoeba* (418 total, 2.6% of the entire proteome) and indicating a 2-fold enrichment in the mitoproteome. These 53 AcMitoCarta proteins arose from diverse prokaryotic donors at various timepoints in evolution, ranging from an Amorphea ancestor to *Acanthamoeba* itself (Figure 2I). The most recent 35 HGTs are not seen in *Dictyostelium*. 32 of the 53 proteins have a predicted MTS, and 41 of these proteins have at least one Pfam domain, providing a foundation for predicting function (Table S2).

Twelve of the 53 HGT AcMitoCarta proteins have a DNA- or RNA-related Pfam, six of which are predicted to be products of recent HGT into *Acanthamoeba* not observed in other amoebozoans. These include a HGT homolog of HupA,^71–73^ a bacterial nucleoid DNA-binding protein typically found in prokaryotes with rare HGTs to eukaryotes (Figure S3D), including to the red algae *Cyanidioschyzon merolae*, where it localizes to chloroplasts.^74^ We hypothesize that AcHupA is a functional mtDNA-binding HGT replacement for a TFAM-like protein. Consistent with this model, AcHupA retains conserved DNA-binding residues seen in bacterial HupA (Figures 2J and S3E). To determine if AcHupA binds DNA, we recombinantly expressed and purified AcHupA (Figure S3F) and examined its DNA-binding capability in an electrophoretic mobility shift assay (EMSA^75^) (Figure 2K). Mutating one of the conserved arginine residues (R66) involved in DNA binding to a glutamate resulted in a slight shift in the protein concentration required to induce an electromobility shift (Figure 2K), suggesting involvement in DNA binding, like the homologous R58 in *Escherichia coli* HupA.^71^ DNA binding is weakened but not abolished because multiple HupA arginine residues bind DNA. Finally, a C-terminally 3xHA-tagged AcHupA construct co-localizes with mtDNA in *Acanthamoeba* (Figures 2L and S3G).

These HGT-derived proteins provide footholds for uncovering unique biology. However, we also predict that many of the gaps in knowledge of pathway members will be filled not by HGT proteins, but by lineage-specific proteins, requiring discovery by empiric biochemistry.

### *Acanthamoeba* mitochondrial complexome

To map uncharacterized AcMitoCarta proteins into macromolecular assemblies with known functions, we conducted complexome profiling. This work builds on a prior analysis of five OXPHOS complexes^35^ by leveraging: (i) MitoIP, (ii) AcMitoCarta, (iii) our ACANB genome annotation^50^, (iv) MitoTOL orthogroups^59^, and (v) analysis of 15 complexes outside of OXPHOS.

*Acanthamoeba* MitoIP mitochondria were solubilized with digitonin or dodecyl-D-maltoside (DDM) and complexes were separated by blue native-polyacrylamide gel electrophoresis (BN-PAGE).^76,77^ In-gel enzyme assays detected OXPHOS complexes (Figure 3A).^78^ Two BN-PAGE lanes (digitonin or DDM) were cut into 60 slices each and analyzed by MS, which detected a total of 1,005 AcMitoCarta proteins (Figure 3B and Table S4). Apparent MWs of each gel slice were calculated based on a standard set of bovine OXPHOS complexes run in an adjacent lane (Figures S4A and S4B). Based on co-migration of conserved AcMitoCarta proteins, we detected 20 mitochondrial complexes including pyruvate dehydrogenase (PDH), electron transfer flavoprotein (ETF), isocitrate dehydrogenase (IDH), methylcrotonyl-CoA carboxylase (MCC), propionyl-CoA carboxylase (PCC), and succinyl-CoA ligase (SUCL), prohibitin (PHB), a mitochondrial contact site and cristae organizing system (MICOS), TOM, subassemblies of translocase of the inner membrane (TIM),^79^ and a COQ8A-COQ9 interaction suggesting a CoQ biosynthesis metabolon (complex Q)^66^ (Figures 3C, D).

Most complex members co-migrated similarly in both detergents, but some differences proved useful (*e.g.*, CI and MICOS peaked separately in DDM) (Figure 3C). Several complexes also show unique patterns of sub-peaks that provide a useful “fingerprint” for uncovering complex members (*e.g.*, CV_2_ dimer plus a higher-order multimer, CV_m_) (Figure 3C). Members of the same complex generally displayed similar abundance (estimated by intensity based absolute quantitation, iBAQ), and iBAQ values can also allow determination of stoichiometry of multimeric subunits, such as the three α and three β subunits in each CV monomer (Figure 3D).

Over 300 AcMitoCarta proteins from orthogroups not seen in human or yeast mitochondria (hereafter called “unique” and rendered in green in Figure 3) were also detected, nearly half of which migrated in high MW regions of the gel (above CII), suggesting participation in large macromolecular assemblies (Figure 3B). We used both BN-PAGE-MS profile correlations based on “bait” profiles of known complexes and manual-inspection matching of complex “fingerprints” to link uncharacterized proteins to known complexes (Figures 3C, 3E). Co-migration hits confirm those reported previously for CI, CIV, and CV^35^ and extend to select previously unreported members and other complexes. For example, we observe 63 conserved members of the large or small mitoribosomes, plus 19 unique co-migration partners, potentially representing mitoribosome members (Figure 3F).

Because BN-PAGE-MS co-migration alone cannot unequivocally distinguish *bona fide* complex membership, we further tested CI, CIV, and CV candidates by IP-MS using HA-epitope tagged complex members validated by microscopy (Figures S2). Mitochondria were isolated from each transgenic line and solubilized with DDM, and then HA-tagged constructs and protein interaction partners were isolated by anti-HA IP (Figure S4D) and analyzed by MS (Table S5). The IP-MS profiles verified association of 12/13 CI co-migration hits, 6/6 CIV co-migration hits, and 9/9 CV co-migration hits (Figures 3G and S4E). Integration of IP-MS and BN-PAGE-MS data revealed several unique features:

**Complex I** proteins exhibit both a high-MW fully assembled complex and low-MW subassemblies including an N-like module and a QP-like module.^80–82^ Thirteen unique CI co-migrating proteins not seen in human CI likely contribute to the higher MW of AcCI relative to those of mammals^76,83^ and plants.^84^ These include four previously unreported proteins seen by both BN-PAGE-MS and IP-MS: ACANB03328 (in both fully assembled CI and the N-like module), and CYB5B, ACANB02186, and ACANB01954 (each in both fully assembled CI and in the QP-like module). ACANB01954 has a rhodanese pfam domain and is predicted to be a relatively recent HGT from gammaproteobacteria (Figure S4F). Our data also help fill gaps for conserved CI members previously lacking BN-PAGE-MS evidence (*e.g.*, MT-ND2, MT-ND6, NDUFV2 (NuoE), NDUFS4, NDUFS6, and NDUFB2).

**Complex II**: A possible *SDHB* gene duplication was reported previously, but the SDHB2 protein was not observed.^33,35^ Here, we find both SDHB paralogs in AcMitoCarta and in CII via BN-PAGE-MS (Figure S4C). Our separate transcriptomic and proteomic profiling shows reciprocal regulation of SDHB1 and SDHB2 during encystation, with SDBH2 being encystation-induced^50^. Here, because our BN-PAGE-MS was conducted with trophozoites, we see higher abundance of SDHB1 (Figure 3D).

**Complex III** was observed as a dimer (Figure 3B), with nine conserved co-migrating subunits including UQCRC2. *Acanthamoeba* lacks UQCRC1 but has another orthogroup member, PMPCB^35^ (Figure S4C), which we confirmed as a member of AcCIII by our parallel structural studies^85^, revealing a unique composition of the matrix-facing heterotetramer of PMPCB and UQCRC2.

**Complex IV** was identified based on co-migration of highly conserved CIV subunits^35^ COX1-2, 3, and 5B—plus BN-PAGE-MS evidence for COX6A1 and COX6B2, and a HIGD2A homolog interacting with CIV (Figures 3C–E). We also observe six additional co-migration partners including possible remote homologs of COX1C, COX4, COX6C, and COX7C, as reported previously^35^—plus BN-PAGE-MS evidence for a second COX7C homolog (COX7C2)—hits now verified by IP-MS (Figure S4E).

**Complex V:** Ten conserved CV members co-migrated, with nine additional co-migration partners including a prokaryotic HGT-derived protein (Figures 3C–E). As reported previously,^35^ we observed co-migration between CV and malate dehydrogenase (AcMito-MDH). Importantly, our IP-MS confirms this surprising hit (Figures 3G and S4E), and iBAQ data indicate that MDH is highly abundant (Figure 3D). These atypical subunits likely contribute to the higher apparent MW of the AcCV_2_ dimer (~2 MDa) (Figure 3E) relative to the BN-PAGE-MS apparent MWs of CV monomers or dimers seen in mammals,^76,83^ yeast,^77^ plants,^84^ *Plasmodium*,^86^ and *Toxoplasma*^87–89^ (although the *Toxoplasma* CV dimer structure also has a high MW ~2.5 MDa due to lineage-specific subunits).^90^ Our cryo-electron microscopy (cryo-EM) studies yield an atomic model of AcCV^85^, verifying the nine additional partners, including an AcMito-MDH dimer integrated into the peripheral stalk through interactions with OSCP and a structural tether—ACANB14707, now named MDH-ATP synthase adaptor (MASA) subunit—physically linking the tricarboxylic acid (TCA) cycle to OXPHOS.

### Bioenergetics pathways not seen in humans

In addition to an atypical OXPHOS system, the AcMitoCarta has bioenergetics pathways not seen in humans, including AOX^44^ and NDH2s^43^ (Figure 4A) and proteins with homologs enriched in anaerobic prokaryotes (see Methods), such as HydA, HydE–G, PFOR, and ASCT1B (Figure 4B). All of these were found in our MitoViaPCP list with high scores (Figure 4C), providing protein-level evidence for the full set of genomic predictions^46^ including three proteins lacking prior MS evidence (HydA, HydE, and HydG) (Figure S1E). IP-MS of HydA indicates an interaction with ferredoxin (FDX) (Figure 4D), suggesting that PFOR could pass electrons to HydA via FDX, as seen in other anaerobes,^91,92^ although this remains to be determined directly. We also confirmed HydA, HydE–G, and PFOR to be mitochondrial via epitope tagging and microscopy (Figure 4E).

A homolog of the bacterial hybrid cluster protein (HCP)—named after its atypical iron-sulfur cluster which contains bridging oxygen^93,94^—also showed strong anaerobic association (Figure 4B). HCP scores highly in our MitoViaPCP list (Figure 4C) and was confirmed to be mitochondrial by microscopy (Figure 4E). Bacterial HCP homologs are thought to catalyze anaerobic conversion of nitric oxide (NO) into nitrous oxide (N_2_O).^93,94^ Eukaryotic HCP proteins^95^ have been reported in the mitoproteomes of the algae *Chlamydomonas reinhardtii*^96,97^ and the anaerobic ciliate *Nyctotherus ovalis*,^98^ but these localizations await rigorous validation. We hypothesize that HCP could function under anoxia in either elimination of toxic NO or in a nitrogen metabolism pathway. We searched our AcMitoCarta for additional nitrogen-related enzymes and found a mitochondrial homolog of the copper-dependent nitrite (NO_2_^−^) reductase NirK^99,100^ (not reported previously^33^), although its functions remain unknown.

### Oxygen and nutrient perturbation profiling

To understand how the mitoproteome and its anaerobic and aerobic pathways are remodeled in response to environmental cues, we cultured *Acanthamoeba* in biological triplicate under 15 distinct environmental conditions, including an O_2_ sweep. All 48 samples (including two sets of triplicate controls) were analyzed by MS and RNA-seq (Figure 5A). In both nutrient-rich and starvation media, we cultured cells for 24 hours under normoxia (21% O_2_), hypoxia (1% O_2_), or near-anoxia (<0.01% O_2_), plus one or six hours of normoxic recovery after 24 hours of near-anoxia. Normoxic rich medium maintains motile *Acanthamoeba* trophozoites, while starvation triggers encystation (Figures 5B, S5A, and S5B). Anoxia caused trophozoites to become nonadherent with atypical morphology, but adherence and normal morphology were regained within 1 hour of re-oxygenation (Figures 5B). In starvation medium, anoxia blocked cyst wall formation, indicating that encystation requires O_2_, consistent with a recent report^23^ (Figure 5B). For separate “carbon and iron” perturbations, glucose was either removed or replaced with galactose or glycerol, and ferrous iron was either removed or replaced with ferric iron, changes that also altered morphology (Figure S5C).

MS and RNA-seq revealed dynamic environment-dependent remodeling of the proteome and transcriptome (Figures S5D and Tables S6, S7). For example, subsets of the AcMitoCarta were increased or decreased based on the carbon or iron nutrients in the medium (Figures 5C and S5D), suggesting nutrient-dependent regulation of select metabolic pathways.

We had hypothesized that in the absence of O_2_, anaerobic pathway members such as HydA and PFOR might increase, but that most of the mitoproteome including OXPHOS would decrease. Unexpectedly, nearly all mitochondrial proteins increased under low O_2_ conditions in both rich medium and starvation medium (Figures 5C and S5D). Using the fraction of total proteomic signal intensity derived from AcMitoCarta genes as a proxy, the relative abundance of the mitoproteome also increased under both hypoxia and anoxia (a result seen for both the nuclear- and mtDNA-encoded subsets of AcMitoCarta) (Figure 5D). In contrast to the proteomics results, only a smaller subset of AcMitoCarta transcripts increased under hypoxia or anoxia (Figure S5D), suggesting both transcriptional and post-transcriptional regulation mechanisms (*e.g.*, decreased relative degradation of mitochondria). Top hits for the most upregulated AcMitoCarta transcripts and proteins under hypoxia and anoxia include PFOR, HydA, HydE–G, and HCP (Figure 5E). Upregulation at both the RNA level and the protein level helps distinguish anaerobic pathway members (*e.g.*, PFOR and HydA) from their parallel aerobic machinery (*e.g.*, PDH and complex I) (Figure 5F). Similar results are seen for aerobic OXPHOS CII–CV compared to the H-cluster and iron-sulfur (Fe-S) cluster (ISC) biogenesis proteins needed for HydA maturation (*e.g.*, HydE–G, ISCA1, ISCA2, and FDX) (Figure 5G). Overall, these data support a model in which hypoxia or anoxia triggers selective maintenance or upregulation of the bulk of the mitoproteome, which can then import and complete assembly of the key mitochondrial PFOR-to-HydA pathway after its transcription has been induced by hypoxia.

An outlier in these patterns is AOX, which shows intriguing O_2_-dependent changes that cannot be readily explained by known AOX biochemistry (Figure 5G). Interestingly, all three NDH2s are increased under hypoxia and anoxia at the protein level, and one is even induced at the RNA level (Figure 5G), raising the provocative hypothesis that NDH2s might have an unexplored role in anaerobic physiology. This NDH2 induced by low O_2_ at the RNA level is likely a matrix-facing internal NDH2 (NDI) based on its phylogenetic affiliation with biochemically characterized homologs in other organisms^101–107^ (Figure S5E).

### H_2_ gas generation by *Acanthamoeba* mitochondria

Whether *Acanthamoeba* contains an enzymatically active HydA that can generate H_2(g)_ has not yet been experimentally determined. The primary sequence of *Acanthamoeba* HydA contains highly conserved metal ligand-binding residues, including residues forming a unique H-cluster that is key for enzymatic activity (Figure S6) and is assembled by HydE–G in other species.^108^ AlphaFold3^109^ predictions of the AcHydA structure reveal a conserved active site including H-cluster residues (Figures 6A and 6B). In HydA homologs from other species, the HydA H-cluster is rapidly inactivated by O_2_.^110–112^ To assess HydA activity, we cultured *Acanthamoeba* under strictly anaerobic conditions in an anoxic glovebox with a catalyst system that removes trace amounts of O_2_. We find that *Acanthamoeba* can survive at least five days of true anoxia (although it does not proliferate under anoxia). To determine whether anaerobically cultured *Acanthamoeba* cells can produce H_2(g)_, we used a H_2(g)_ specific Clark-type (amperometric) electrode to detect H_2(g)_ and cell-permeable reduced methyl viologen as an electron donor, which is a standard assay technique for bidirectional hydrogenases.^111^ As a control, we used aerobically cultured cells (normoxia, 21% O_2_), brought into the anaerobic glovebox for removal of O_2_ prior to the assay. We find that anaerobically cultured *Acanthamoeba* can produce H_2(g)_, but aerobically cultured *Acanthamoeba* lacks H_2(g)_ production capacity (Figure 6C).

Irrespective of its primary physiologic role, in characterized examples such as prokaryotic HydA,^110,111,113^ HydA is fundamentally a bidirectional enzyme that can operate in either the H_2(g)_ production or consumption (hydrogenase) directions. We find that *Acanthamoeba* HydA can also operate in the H_2(g)_ consumption direction using (oxidized) methyl viologen as an electron acceptor that turns blue upon reduction (Figure 6D), a classical assay in this field.^111^ While *Acanthamoeba* could theoretically oxidize H_2(g)_ produced by a nearby H_2(g)_ producing organism, our perturbation profiling showing an increase in *HydA* transcripts under anoxia suggests that a primary role for HydA is H_2(g)_ production, using H^+^ as an electron acceptor when O_2_ is absent.

To further test this H_2(g)_ production activity, we cultured *Acanthamoeba* under anaerobic conditions and then isolated mitochondria from these cells under anaerobic conditions. We find the specific activity of H_2(g)_ production to be higher in anoxic mitochondria than in whole cells (Figure 6E), consistent with the mitochondrial localization of HydA. Exposing these anoxic mitochondria to air (21% O_2_) decreased the H_2(g)_ production activity (Figure 6F), consistent with the known O_2_ sensitivity of the HydA H-cluster. This would provide a rapid O_2_ dependent post-translational control of HydA enzyme activity, in addition to the observed O_2_ dependent transcriptional regulation of *HydA* (Figure 5F). Together, these results provide evidence for an O_2_ regulated mitochondrial HydA pathway (Figure 6G) with capacity for anoxic H_2(g)_ production through use of H^+^ as an electron acceptor.

### Anaerobic bioenergetics in clinically derived *Acanthamoeba* lines

The above work was conducted with the genomic reference Neff strain, which is classified as genotype T4 Neff. We conducted parallel anaerobic physiology studies with a panel of established clinical lines^114–116^ derived from human cornea infections (Figure 7). Like the Neff reference strain, we find that representative lines from genotypes T4A, T4B, T4C, and T4D exhibit rapid morphologic recovery from anoxia (Figure 7A), H_2(g)_ production capacity (Figure 7B), and hydrogenase activity (H_2(g)_ consumption activity) (Figure 7C). Together, these experiments provide evidence that the anaerobic PFOR-to-HydA pathway is active in clinically derived *Acanthamoeba*.

To test the physiologic relevance of mitochondrial bioenergetics pathways under either normoxia or anoxia, we used of set of small molecule chemical inhibitors of mitochondrial enzymes: (i) nitazoxanide, a PFOR inhibitor used as an antiparasitic drug,^117^ with demonstrated activity in *Acanthamoeba*,^26,118,119^ (ii) diphenylene iodonium chloride (DPI), which inhibits flavoenzymes including NDH2 based on work in *Plasmodium*^120^ and *Trypanosoma*^121^ (and potentially other flavoproteins^122^), (iii) 2-heptyl-4-hydroxyquinoline *N*-oxide (HQNO), a CoQ-site NDH2 inhibitor^123^ that also possibly targets the CoQ-sites of CIII and DHODH,^122^ (iv) oligomycin, a CV (ATP synthase) inhibitor, with previously reported activity against *Acanthamoeba*^124^, and (v) SHAM, an AOX inhibitor with reported activity in *Acanthamoeba* at high concentrations.^23^ To quantify the impact of these inhibitors, we used a luciferase-based cellular ATP assay.^122^ Consistent with prior work in *Acanthamoeba*, we observe an IC50 of ~9 μM for oligomycin under normoxia^124^ (Figures 7D and S7). Importantly, we observe significant activity of nitazoxanide under strict anoxia (Figures 7D and S7), confirming the importance of mitochondrial PFOR in *Acanthamoeba’s* anaerobic physiology. At the concentrations tested here (up to 50 μM), the AOX inhibitor SHAM had no significant activity. Surprisingly, both DPI and HQNO showed greater inhibitory activity under anoxia than under normoxia (Figures 7D and S7). These results indicate that a flavoprotein and a protein with a CoQ site play key roles in *Acanthamoeba’s* anaerobic physiology, possibly including one of the NDH2 enzymes, such as the NDI1 that we observed to be upregulated under anoxia (Figure 5G). These patterns of inhibition were seen in both the reference Neff strain and a set of clinically derived lines (Figures 7D and S7). Together, these results demonstrate the importance of mitochondrial aerobic and anaerobic bioenergetics pathways in *Acanthamoeba* physiology and provide a toolkit for dissecting the roles of these pathways in infectious disease biology.

## DISCUSSION

Here we have generated and released a high-quality, experimentally-defined inventory of 1,122 mitochondrial proteins in the Amoebozoa branch of biology—a key evolutionary outgroup to opisthokonts such as humans and yeast. The 741 AcMitoCarta proteins shared with either human or yeast mitochondria provide a foundational set for defining mitochondrial proteins likely present in the last Amorphea common ancestor (Figure 1A). The AcMitoCarta also contributes to our map of the LECA mitoproteome, directly supporting 44% of our LECA mitoproteome^59^. The AcMitoCarta is available online at mitocarta.org.

The AcMitoCarta paints a rich mosaic of mitochondrial protein ancestry including ancient vertically inherited genes of prokaryotic origin (~65%), eukaryotic innovations (~30%, including many lineage-specific proteins), and a relatively high rate of recent HGT from prokaryotes (~5%). Our analysis of HGT proteins in the AcMitoCarta highlighted AcHupA as a mtDNA-binding protein, providing an anchor for studying *Acanthamoeba* mtDNA pathways, which are highly divergent. Over 300 lineage-specific AcMitoCarta proteins are uncharacterized and provide opportunities for future work.

AcMitoCarta highlights an elaborate network of unusual metabolic pathways in *Acanthamoeba*. Malate metabolism provides a key example. *Acanthamoeba* mitochondria contain at least two atypical enzymes that generate malate, (i) the malate synthase-isocitrate lyase (MS-ICL) fusion protein^33,125^ that enables its glyoxylate cycle (not seen in humans) and (ii) a class-I fumarate hydratase^33^ (associated with anaerobic organisms). Of note, the *Acanthamoeba* genome also encodes a class-II fumarase (ACANB03214), present in the mitochondria of humans, with a PCP score of −0.57, providing evidence for non-mitochondrial localization, conflicting with prior predictions.^33^ AcMitoCarta also includes at least two enzymes that catabolize malate: (i) a malic enzyme (ME1) and (ii) a mito-MDH. The *Acanthamoeba* genome also encodes two non-mitochondrial MDHs, which have negative PCP scores. How malate metabolism is regulated under variable conditions is a fascinating area for future investigation. Even mito-MDH is unusual, as defined in our cryo-EM work^85^ revealing a physical link between mito-MDH and ATP synthase by a dedicated scaffolding protein. Malate appears to lie at the center of the TCA cycle, OXPHOS, and the glyoxylate cycle.

Our integrated complexome profiles and IP-MS studies provide valuable resources for mapping unusual features of additional macromolecular assemblies, such as those of complex I. The observation of both fully assembled complex I and N-like and QP-like modules^80–82^ was striking. Whether these N- or QP-like modules are *in vivo* CI assembly pathway intermediates like those observed in plants^84^ and mammals,^82^ free complexes *in vivo* like those seen under some conditions in *Neurospora*,^126^ or *in vitro* artifacts due to solubilization remains unknown. The N-like module is more abundant in DDM than in digitonin, suggesting detergent-sensitivity. Assembly and disassembly of N- and QP-like modules could regulate OXPHOS, reactive oxygen species production, and alternative ETC pathways.

An important feature of our work is that it experimentally delineates both aerobic and anaerobic pathways for mitochondrial ATP production. Most extant mitochondria generate ATP either strictly via aerobic (*e.g.*, humans) or anaerobic (*e.g.*, *Trichomonas*) pathways. While the identification of H_2_ gas generation capacity by *Acanthamoeba* mitochondria provides experimental evidence that the organelle can use a proton as an electron acceptor in the absence of O_2_, the primary *in vivo* directionality of the hydrogenase under various conditions and the endogenous electron donors remain to be determined experimentally. Another future challenge is to determine the environmental contexts in which *Acanthamoeba’s* anaerobic and aerobic pathways operate. As a foundation, our perturbation profiling demonstrated that under anoxia, *Acanthamoeba* upregulates and activates its mitochondrial hydrogenase pathway.

At present, we have few if any effective medical therapies for treating *Acanthamoeba* infections, and our work highlights the potential of targeting AcMitoCarta pathways. Motivated by the hydrogenase activity seen in human keratitis-derived lines, targeting anaerobic pathways in *Acanthamoeba* seems particularly appealing, as they are not present in the human host and presumably can be targeted more safely. Metronidazole provides a key proof of principle for safely targeting anaerobic pathways in the cytosol of protozoan pathogens such as *Giardia*,^127^ and metronidazole has been suggested as a potential part of a drug cocktail for *Acanthamoeba*.^128–130^ The 381 AcMitoCarta proteins lacking orthologs in humans or yeast are an especially high priority set for nominating potential drug targets. Simultaneous targeting of both aerobic and anaerobic pathways could provide an effective combination.

*Acanthamoeba*’s highly-branched mitochondrial bioenergetics pathways are unique among organisms that are experimentally tractable. The short list of other eukaryotes with experimentally demonstrated hydrogenase activity includes: (i) green algae with chloroplast-localized enzymes,^131–135^ (ii) *Trichomonas*^5,131,132^ and *Neocallimastix*^133^ with hydrogenosome-localized activity (lacking OXPHOS), and (iii) *Giardia*^134^ and *Entamoeba*^135^ with cytosolic hydrogenases (both with mitochondrion-related organelles lacking OXPHOS). The anaerobic mitochondrion-like organelles of *Blastocystis*^136^ and *Nyctotherus ovalis*^98,137,138^ contain hydrogenases, but these organelles lack OXPHOS CIII, CIV, and CV. Other eukaryotes with limited anaerobic mitochondrial pathways lack HydA (*e.g.*, *Euglena gracilis*^139,140^ and *Ascaris*^7^). Genomic predictions suggest a small number of additional eukaryotes with hydrogenase genes^9^ (*e.g.*, reported cytosolic HydA in *Naegleria*^141^), but their enzymatic activities, subcellular localizations, and mitoproteomes have not yet been rigorously defined. *Acanthamoeba* is remarkable because it provides an example of a mitochondrion with both a complete aerobic OXPHOS (CI–CV) and an anaerobic PFOR-to-HydA pathway. With the addition of its three NDH2s, AOX, ETF complex, and other ETC inputs (Figure 6G), *Acanthamoeba* appears to have the largest, most branched mitochondrial bioenergetics system that has been empirically characterized to date—with both a high-accuracy experimental mitoproteome and documented biochemical activities for OXPHOS CI–CV and an anaerobic hydrogenase.

An open question is to what extent the earliest mitochondria had both aerobic and anaerobic pathways for energy conservation.^3,4^ In companion MitoTOL work^59^, we examined the phylogeny and predicted localization of anaerobic pathways across the tree of life and found that some anaerobic proteins (HydA, HydE, HydF, HCP, Class-I fumarase, and ASCT1B) have homologs with predicted mitochondrial localization that are found in clades spanning diverse eukaryotic lineages—suggesting ancient ancestry of these anaerobic pathways in mitochondria potentially dating back to LECA. Thus, the highly branched ETC of *Acanthamoeba* mitochondria, with both aerobic and anaerobic branches toggled by O_2_ level, might resemble the ancestral composition and dynamic physiology of the LECA mitochondrial ETC.

### Limitations of the study

For organisms that cannot be cultured or harvested at scale for our experimental MitoTOL approach, a bioinformatics-based strategy remains valuable. However, through MitoTOL, we have already shown excellent performance and broad applicability in six divergent organisms (*Acanthamoeba*, *Babesia*, *Giardia*, *Trypanosoma*, *Leishmania*, and *Arabidopsis*).

The Percoll gradient used in this study provided high-purity mitochondria, as did OptiPrep (iodixanol) gradients in other MitoTOL work. We recommend direct comparison of Percoll and OptiPrep in future work.

Under the detergent conditions we optimized for our BN-PAGE-MS, we observed multiple higher-order complexes including dimers of CIII and CV, yet we do not see evidence for OXPHOS supercomplexes like those seen in mammals,^76,142^ plants,^84^ *Plasmodium*,^86^ and *Toxoplasma*.^143^ Whether *Acanthamoeba* has supercomplexes *in vivo* remains unknown.

This work was primarily focused on *Acanthamoeba* trophozoites because we currently lack methods to isolate mitochondria from cysts, which have an extremely durable cell wall comprised of proteins and carbohydrates.^116,144,145^ We separately report how the *Acanthamoeba* mitoproteome is remodeled during encystation^50^.

Phylogenetic inference at deep timescales is challenging due to phylogenetic artifacts and potentially complex evolutionary histories involving multiple HGTs, duplications, and losses; we attempted to account for these alternative scenarios, but we recognize that the limitations of current phylogenetic methods may affect inferences about deep timepoints such as LECA.

## RESOURCE AVAILABILITY

### Lead contact

Further information and requests for resources and reagents should be directed to and will be fulfilled by the lead contact, Vamsi K. Mootha (vamsi_mootha@hms.harvard.edu).

### Materials availability

All unique materials are available upon request from the lead contact.

## STAR★METHODS

### EXPERIMENTAL MODELS

#### Cell culture

For our mitochondrial proteomics work, we selected the reference Neff strain of *Acanthamoeba castellanii* for the following reasons: (i) In VEuPathDB (and AmoebaDB), the primary bioinformatics resource for parasitologists, the Neff strain provides the only transcriptome, the only proteome, and the only reference nuclear genome for *Acanthamoeba castellanii*. The Neff strain is also the reference strain for the *Acanthamoeba* genus in both NCBI GenBank/Refseq and Uniprot. (ii) The Neff strain provides the primary reference mtDNA sequence.^32^ (iii) We markedly improved genome annotation quality for this reference Neff strain. Please see our separate ACANB manuscript^50^ (increased annotation accuracy from 52% to 98%). (iv) The Neff strain has a long history of lab safety. (v) Neff is the primary reference strain for *Acanthamoeba* biology. (vi) The Neff strain is classified as genotype T4, which is a common genotype seen in human infections.

Unless otherwise stated, *Acanthamoeba castellanii* strain Neff was cultured in vented tissue culture flasks (75-cm or 182-cm side-arm flasks) with standard medium (SM) (20 g/L proteose peptone, 1 g/L yeast extract, 4 mM MgSO_4_, 0.4 mM CaCl_2_, 3.4 mM sodium citrate, 50 uM Fe(NH_4_)_2_(SO_4_)_2_, 2.5 mM Na_2_HPO_4_, 2.5 mM KH_2_PO_4_, pH 6.5, 100 mM glucose, Penicillin-Streptomycin 50 U/mL) at 30 °C in stationary dry incubators. Transfected lines were maintained in SM with geneticin (G418, 25 ug/mL).

Details for clinically derived lines^114,115^ are provided in the Key Resources Table. Genotypes were confirmed as described previously.^116^ Clinically-derived lines were cultured using the same procedures as the Neff strain.

### METHOD DETAILS

#### Molecular cloning of plasmids

DNA constructs were cloned into the ectopic circular DNA plasmid GAPDH-EGFP-polyAtailRev vector^144^ (from John Samuelson, Boston University). Constructs were amplified via PCR using Q5 Hotstart Master Mix 2x (New England BioLabs, Cat. #M0494S) and PCR fragments were assembled with NEBuilder HiFi DNA Assembly Master Mix (New England Biolabs, Cat. #E2621). The assembled plasmids were transformed into chemically competent 5-alpha *E. coli* (NEB Cat. #C2987H) for scale-up and preparation. Isolated plasmids were sequenced via whole plasmid sequencing.

#### Plasmid transfections

Prior to the day of transfection, wild type *A. castellanii* was cultured into early log phase growth (confluency 10–50%) (axenic cultures, adherent cell monolayers, standard media, vented side-arm tissue culture flasks, 30 °C, stationary dry incubator). On the day of transfection, trophozoites were harvested into Encystation Medium (EM) (20 mM Tris-HCl, pH 8.8, 100 mM KCl, 8 mM MgSO_4_, 0.4 mM CaCl_2_, 1 mM NaHCO_3_) with Pen-Strep by decanting the spent culture media, adding EM (10 mL), mechanically detaching the cells by hitting the back of the flask five times, transferring the cell suspension to a 15-mL tube, and harvesting a cell pellet by centrifugation (1,000 *g*, 5 min, 4 °C). The supernatant was decanted, the cells were resuspended in EM (10 mL) by gentle pipetting and then pelleted again by centrifugation (1,000 *g*, 5 min, 4 °C). This washed cell pellet was resuspended in EM and manually counted by hemocytometer. A 25-cm side arm culture flask was seeded with 5x10^5^ cells in EM (2 mL final volume) and incubated to allow the cells to adhere (30 min, 30 °C) (typically cell density after seeding: 5–10% confluency). Plasmid DNA (4 ug in a sterile tube) was heated briefly (5 min, 85 °C) and then prepared for transfection with Lipofectamine 3000. Lipofectamine 3000 (7.5 uL) was diluted in EM (125 uL). The plasmid DNA (4 ug) was diluted with EM to a final volume of 117 uL, and then P3000 reagent (8 uL) was added. The diluted Lipofectamine 3000 (125 uL) and the DNA mixture (125 uL) were mixed and incubated (15 min, rt). This DNA-lipofectamine mixture (250 uL) was added to the adherent trophozoites in EM (1 mL) and incubated (4 h, 30 °C). The EM and transfection mixture was removed by pipetting and replaced with SM with antibiotics (Pen-Strep) (2 mL) and incubated (1 day, 30 °C). One day after transfection, to initiate selection, the spent media was decanted, SM+G418 (12 ug/mL G418) was added, and the cultures were incubated again (3 days, 30 °C). The selection media was exchanged every 3 days until adherent trophozoites began to proliferate.

##### Isolation of mitochondria

###### Preparation of isotonic Percoll gradients

Percoll is supplied as a 23% colloidal suspension in water. Here, this solution is considered “100% Percoll”. 50% isotonic Percoll (0.28 M sucrose, 10 mM HEPES-KOH pH 7.2, 0.2 mM EDTA, 50% Percoll, v/v) was prepared by mixing equal volumes of 100% Percoll and “2X SHEP” buffer (560 mM sucrose, 20 mM HEPES-KOH pH 7.2, 0.4 mM EDTA, 2x Roche complete protease inhibitor mix, 0.5 mM freshly added PMSF). Lower percentages (35% or 25%) of isotonic Percoll were prepared by mixing the appropriate volumes of 50% isotonic Percoll and “1X SHEP” buffer (0.28 M sucrose, 10 mM HEPES-KOH, pH 7.2, 0.2 mM EDTA, 1x Roche complete protease inhibitor mix, 0.25 mM PMSF). Immediately prior to use, three-step gradients of Percoll were prepared on ice in Beckman Coulter ultracentrifuge tubes (14 x 89 mm, Polypropylene, Cat # 331372) (bottom layer, 2 mL of 50% isotonic Percoll; middle layer, 2 mL of 35% isotonic Percoll; top layer, 4 mL of 25% isotonic Percoll).

###### Isolation of wild type mitochondria for proteomics

*Acanthamoeba castellanii* strain Neff, wild type, was maintained in log phase growth in standard media for three days prior to the experiment. The experimental cultures (182-cm vented flasks, 30 mL standard medium) were inoculated with 3x10^6 cells, and the cultures were incubated (30 °C, stationary, 38 hours) until the cells reached confluency. For each biological replicate, fifteen 182-cm flasks (affording approximately 6x10^8 cells after harvest and washing) were used for a single preparation of mitochondria.

Both adherent cells and non-adherent cells were harvested. The following steps were conducted at 4 °C, in a cold room and on ice when possible, and using pre-cooled equipment and pre-cooled, freshly prepared buffers. The culture flasks were transferred to a 4 °C cold room. Using the existing media in the flasks, the adherent cells were mechanically detached from the tissue culture flask (hitting the back of the flask 5 times, hitting the side of the flask 5 times, and then rocking the flask back and forth 5 times) and transferred to 250-mL plastic conical tubes on ice. The harvested cells were centrifuged (800 *g*, 5 min, 4 °C) to collect intact cells. The cells were resuspended in phosphate buffered saline (PBS) with 0.5 mM PMSF (50 mL per biological replicate harvested, 4 °C) and then centrifuged (800 *g*, 5 min, 4 °C) again to collect washed intact cells. The supernatants were decanted and disposed, and the cell pellets were resuspended in sucrose-HEPES-EDTA buffer with BSA and 2x protease inhibitors (SHEB2xP) (0.28 M sucrose, 10 mM HEPES-KOH, pH 7.2, 0.2 mM EDTA, 1 mg/mL fatty acid free BSA, 2x Roche complete protease inhibitor mix, 0.5 mM freshly added PMSF) (final volume to 30 mL, 4 °C).

Aliquots of whole-cell samples (300 uL) were centrifuged (1,000 *g*, 10 min, 4 °C) to isolate washed cell pellets, and then either (i) frozen in N_2(l)_ for proteomics or (ii) resuspended in PBS (100 uL, 4 °C), mixed (1:1, v/v) with “2x FGP” fixative (2.5% paraformaldehyde, 5% glutaraldehyde, 0.06% picric acid in 0.2 M cacodylate buffer, pH 7.4) (100 uL, freshly prepared), and stored at 4 °C, protected from light, for electron microscopy (final fixative concentrations: 2.5% glutaraldehyde 1.25% paraformaldehyde and 0.03% picric acid in 0.1 M sodium cacodylate buffer, pH 7.4).

The rest of the cell suspension was homogenized with a Dounce homogenizer (15 mL of cell suspension in a 15-mL glass Dounce homogenizer on ice in a cold room, 15 strokes with loose pestle, 10 strokes with tight pestle, minimizing bubble formation). The whole-cell homogenate was transferred into a 50-mL conical tube on ice. The combined cell homogenates were centrifuged at slow speed (800 *g*, 10 min, 4 °C) to pellet intact cells and nuclei. The slow-spin supernatant was recovered, transferred to a clean 50-mL conical tube on ice, and centrifuged again at slow speed (800 *g*, 10 min, 4 °C) to clear any remaining intact cells or nuclei. The slow-spin supernatant was recovered, transferred to a clean 50-mL conical tube on ice, and centrifuged at high speed (8,000 *g*, 10 min, 4 °C) to pellet mitochondria. The high-speed supernatant was decanted and disposed. The high-speed pellet was gently washed with SHEBP (0.28 M sucrose, 10 mM HEPES-KOH, pH 7.2, 0.2 mM EDTA, 1 mg/mL fatty acid free BSA, 1x Roche complete protease inhibitor mix, 0.25 mM freshly added PMSF) (0.5 mL, 4 °C) to remove the “buffy coat” (contaminating light membranes). The brown mitochondrial pellet was gently resuspended, first with a glass rod into a small volume of buffer (~1 mL, SHEBP, 4 °C), and then by gently pipetting (SHEBP, 4 °C, final volume to 12 mL). The resuspended mito pellet was transferred to a 15-mL conical tube and further resuspended by gentle pipetting with a 10- mL pipette. The resuspended mitochondria were centrifuged at slow speed (600 *g*, 10 min, 4 °C). The slow-spin supernatant was recovered, transferred to a clean 15-mL conical tube on ice, and centrifuged at high speed (8,000 *g*, 10 min, 4 °C). The mitochondria were gently resuspended in SHEP (0.28 M sucrose, 10 mM HEPES-KOH, pH 7.2, 0.2 mM EDTA, 1x Roche complete protease inhibitor mix, 0.25 mM PMSF) (400 uL, 4 °C).

300 uL aliquots of these resuspended mitos were loaded onto freshly prepared three-step (50%, 35%, 25%) isotonic Percoll gradients (see details above in separate methods section), and ultra-centrifuged (SW 41 Ti rotor, 18,500 rpm, 42,261 average *g*, 60 min, 4 °C).

Aliquots of the remaining resuspended mitochondria were centrifuged (15,000 *g*, 10 min, 4 °C) to isolate crude mitochondria pellets, and then either (i) frozen in N_2(l)_ for proteomics or (ii) resuspended in PBS (100 uL, 4 °C), mixed with 2x FGP fixative (100 uL, freshly prepared), and stored at 4 °C, protected from light, for electron microscopy.

After ultracentrifugation, the major band of material (at the 25%-35% Percoll step gradient interface) were isolated using a 5-mL syringe and an 18 G needle, puncturing through the side of the ultracentrifuge tube to access this layer of material (~1–2 mL of volume was collected). This material was transferred to a 15-mL centrifuge tube, and SHEP (12 mL, 4 °C) was added to dilute the Percoll. The isolated mitochondria were centrifuged (15,000 *g*, 10 min, 4 °C). The resultant pellet of isolated mitochondria was gently resuspended in SHEP (200 uL, 4 °C) and split into aliquots. The samples were centrifuged (15,000 *g*, 10 min, 4 °C) and the supernatants were discarded, isolating the mito pellets. The proteomics samples were immediately snap frozen in N_2(l)_. The sample for electron microscopy was resuspended in PBS (100 uL, 4 °C), 2x FGP fixative (100 uL, freshly prepared) was added, and the sample was stored at 4 °C, protected from light.

###### Mitochondrial immunoprecipitation (MitoIP) for proteomics

*Acanthamoeba castellanii* strain Neff transfected with a plasmid encoding the MitoIP construct 3xHA-eGFP-TOM7 was maintained in log phase growth in standard media with G418 (25 ug/mL) for three days prior to the experiment. The experimental cultures (182-cm vented flasks, 30 mL standard medium) (note lack of G418 in the media for the experimental cultures) were inoculated with 2x10^6 cells, and the cultures were incubated (30 °C, stationary, 41 hours) until the cells reached confluency. For each biological replicate in this experiment, fifteen 182-cm flasks (affording approximately 5x10^8 cells after harvest and washing) were used. A typical input for a single MitoIP in other experiments would be five confluent 182-cm flasks.

Both adherent cells and non-adherent cells were harvested. The following steps were conducted at 4 °C, in a cold room and on ice when possible, and using pre-cooled equipment and pre-cooled, freshly prepared buffers. The culture flasks were transferred to a 4 °C cold room. Using the existing media in the flasks, the adherent cells were mechanically detached from the tissue culture flask (hitting the back of the flask 5 times, hitting the side of the flask 5 times, and then rocking the flask back and forth 5 times) and transferred to 250-mL plastic conical tubes on ice. The harvested cells were centrifuged (800 *g*, 5 min, 4 °C) to collect intact cells. The cells were resuspended in phosphate buffered saline (PBS) with 0.5 mM PMSF (50 mL per biological replicate harvested, 4 °C) and then centrifuged (800 *g*, 5 min, 4 °C) again to collect washed intact cells. The supernatants were decanted and disposed, and the cell pellets were resuspended in sucrose-HEPES-EDTA buffer with BSA and 2x protease inhibitors (SHEB2xP) (0.28 M sucrose, 10 mM HEPES-KOH, pH 7.2, 0.2 mM EDTA, 1 mg/mL fatty acid free BSA, 2x Roche complete protease inhibitor mix, 0.5 mM freshly added PMSF) (final volume to 50 mL, 4 °C).

Aliquots of whole-cell samples (600-uL or 300-uL) were centrifuged (1,000 *g*, 10 min, 4 °C) to isolate washed cell pellets, and then either (i) frozen in N_2(l)_ for proteomics or (ii) resuspended in PBS (100 uL, 4 °C), mixed with 2x FGP fixative (100 uL, freshly prepared), and stored at 4 °C, protected from light, for electron microscopy.

The rest of the cell suspension was homogenized with a Dounce homogenizer (15 mL of cell suspension in a 15-mL glass Dounce homogenizer on ice in a cold room, 15 strokes with loose pestle, 10 strokes with tight pestle, minimizing bubble formation). The whole-cell homogenate was transferred into a 50-mL conical tube on ice. The combined cell homogenates were centrifuged at slow speed (800 *g*, 10 min, 4 °C) to pellet intact cells and nuclei. The slow-spin supernatant was recovered, transferred to a clean 50-mL conical tube on ice, and centrifuged again at slow speed (800 *g*, 10 min, 4 °C) to clear any remaining intact cells or nuclei. The slow-spin supernatant was recovered and transferred to a clean 50-mL conical tube on ice.

To isolate crude mitochondria fractions (the input material for the MitoIP), 1.5-mL aliquots of the slow-spin supernatant were centrifuged (15,000 *g*, 10 min, 4 °C), the supernatant was discarded, the pellets were isolated, and then either (i) frozen in N_2(l)_ for proteomics or (ii) resuspended in PBS (100 uL, 4 °C), mixed with 2x FGP fixative (100 uL, freshly prepared), and stored at 4 °C, protected from light, for electron microscopy.

The remaining slow-speed spin supernatants were evenly split into three 15-mL tubes on ice (~14 mL per tube) for anti-GFP magnetic microbead IPs. Magnetic microbeads (75 uL/tube) were added and incubated (4 °C, 60 min, gentle rotation, in cold room). Working in a cold room, pre-separation filters (30 μm) and an LS column was placed into a magnetic QuadroMACS multistand separator and equilibrated with SHE buffer (3 mL). The immunocapture was applied to pre-separation filters (30 μm) and the column in 3-mL steps. The columns for MitoIP and MitoIP + Percoll were washed with SHEP buffer (4x 4 mL, 4 °C). The columns for MitoIP + Proteinase K + Percoll were washed with SHE buffer (4x 4 mL, 4 °C) (*N.b.*, no protease inhibitors).

The columns for MitoIP and MitoIP + Percoll were removed from the magnet and the immunocapture was gently flushed out in SHEP (4 mL, 4 °C) with a plunger. Additional SHEP (4 mL, 4 °C) was added. The mitochondria were pelleted by centrifugation (12,000 *g*, 3 min, 4 °C).

The columns for MitoIP + Proteinase K + Percoll were removed from the magnet and the immunocapture was gently flushed out in SHE + Proteinase K (4 mL) with a plunger. This tube with the eluate was moved from the cold room to ambient room temperature (~20 °C), mixed gently by inverting twice, and then incubated (10 min, rt). To stop the protease treatment, the tube was placed back on ice and PMSF was added to a final concentration of 500 uM (to 4 mL reaction, added 20 uL of 100 mM PMSF stock). SHEB2xP (8 mL, 4 °C) was added to further inhibit the proteinase activity. The mitochondria were pelleted by centrifugation (12,000 *g*, 6 min, 4 °C).

The samples for MitoIP only were gently resuspended in SHEP (270 uL, 4 °C) and aliquoted for proteomics and electron microscopy. The samples were centrifuged (15,000 *g*, 10 min, 4 °C) and the supernatants were discarded, isolating the pellets. The proteomics and immunoblot samples were immediately snap frozen in N_2(l)_. The sample for electron microscopy was resuspended in PBS (100 uL, 4 °C), 2x FGP fixative (100 uL, freshly prepared) was added, and the sample was stored at 4 °C, protected from light.

The samples for MitoIP + Percoll and MitoIP + Proteinase K + Percoll were gently resuspended in SHEP (200 uL, 4 °C), loaded onto freshly prepared three-step (50%, 35%, 25%) isotonic Percoll gradients (see details above in separate methods section), and ultra-centrifuged (SW 41 Ti rotor, 18,500 rpm, 42,261 average *g*, 60 min, 4 °C). The major bands of material from each Percoll gradient were isolated using a 5-mL syringe and an 18 G needle, puncturing through the side of the ultracentrifuge tube to access this layer of material (~2 mL of volume was collected). The material for MitoIP + Percoll samples were isolated from the 25%-35% Percoll step gradient interface. The material for MitoIP + Proteinase K + Percoll samples were isolated from the 35%-50% Percoll step gradient interface. These materials were transferred to 15-mL centrifuge tubes, and SHEP (12 mL, 4 °C) was added to dilute the Percoll. The isolated mitochondria were centrifuged (15,000 *g*, 10 min, 4 °C). The resultant pellet of isolated mitochondria was gently resuspended in SHEP (270 uL, 4 °C) and split into aliquots for proteomics and electron microscopy. The samples were centrifuged (15,000 *g*, 10 min, 4 °C) and the supernatants were discarded, isolating the pellets. The proteomics and immunoblot samples were immediately snap frozen in N_2(l)_. The sample for electron microscopy was resuspended in PBS (100 uL, 4 °C), 2x FGP fixative (100 uL, freshly prepared) was added, and the sample was stored at 4 °C, protected from light.

##### Electron microscopy

###### Transmission electron microscopy

Cell pellets or pellets of mitochondria were fixed in FGP fixative (see above, >2 hours, room temp), washed in 0.1 M cacodylate buffer, postfixed with 1% (w/v) osmium tetroxide (OsO_4_), 1.5% (w/v) potassium ferrocyanide (K_4_[Fe(CN)_6_]) (1 hour, room temp), washed twice in water and once in maleate buffer (50 mM maleate, pH 5.15), and then incubated in 1% (w/v) uranyl acetate in maleate buffer (1 hour, room temp), followed by two washes in water and subsequent dehydration in increasing concentrations of ethanol (10 min each: 50%, 70%, 90%, twice in 100%). The samples were then placed in propyleneoxide for one hour and infiltrated overnight in a 1:1 mixture of propyleneoxide and Spurr’s low viscosity resin (Electron Microscopy sciences, Hatfield, PA). The following day, the samples were embedded in Spurr’s resin and polymerized (60 °C, 48 hours). Ultrathin sections (80 nm) were cut on a Reichert Ultracut-S microtome, picked up onto copper grids stained with lead citrate, and examined in a JEOL 1200EX Transmission electron microscope, with images recorded using an AMT 2k CCD camera.

###### Quantification of mitochondria from transmission electron microscopy

Mitochondrial area relative to the total area of cellular material was determined using transmission electron microscopy (TEM) images of samples collected during the mitochondrial isolation procedure. Representative low power TEM fields (approximately 345 square microns of total area) were selected for quantitative analysis using the National Institutes of Health (NIH) *FIJI* software. Using the freehand selection tool, mitochondrial material or non-mitochondrial material were selected as regions of interest (ROIs) and the pixel areas were quantified with the *FIJI* ROI Manager. For whole cell samples, relative mito area was calculated as [summed mito area] / [total cell area]. For organelle samples, relative mito area was calculated as [summed mito area] / ([summed mito area] + [summed non-mito material area]).

##### Antibodies and immunoblots

###### Immunoblots

Samples for immunoblot analyses were lysed in RIPA buffer (Sigma Cat. #20-188) with protease inhibitors (cOmplete^™^, Protease Inhibitor Cocktail, Roche Cat. # 65741000). Protein quantitation was performed by BCA assay per standard protocol (microplate assay, Thermo Cat. #23225). Immunoblot samples were diluted in RIPA buffer to equal protein concentrations, mixed with lithium dodecyl sulfate (LDS) loading buffer with dithiothreitol (DTT) (10 mM final concentration), and denatured by heating (95 °C, 30 min). After SDS-PAGE (4–12% Tris-glycine gel, 135 V, 60 min, rt) proteins were transferred to PVDF and the membrane was blocked with EveryBlot Blocking Buffer (10 min, rt, gentle rocking) (BioRad Cat. #12010020) and then incubated with primary antibody (4 °C, overnight, gentle rocking) (anti-pan-actin 1:1000 v/v, Cell Signaling Cat. #8456S; anti-HA 1:1000 v/v, Cell Signaling C29F4, Cat. #3724; each in EveryBlot) or streptavidin-HRP (Thermo Cat. #21126, 0.3 ug/mL in EveryBlot). The PVDF was washed with TBST (3x 5 min, rt, gentle rocking), incubated with secondary antibody where indicated (*e.g.*, anti-Rabbit antibody Cell Signaling Cat. # 7074S, 1:10,000 in EveryBlot, 1 h, rt, gentle rocking), washed with TBST (3x 5 min, rt, gentle rocking), and then incubated with Clarity Western ECL Substrate (5 min, rt, gentle rocking; BioRad Cat. #1705061) prior to imaging.

###### Production of a custom anti-AcMICU1 antibody

The intermembrane space (IMS) protein mitochondrial calcium uptake 1 (MICU1) from *Acanthamoeba castellanii* (AcMICU1, ACANB11408) was cloned into a pET28a(+) vector with an N-terminal 8xHis tag and a TEV cleavage site. AcMICU1 was expressed in NEB^®^ 5-alpha competent *E. coli* in Luria-Broth (LB) containing kanamycin (50 ug/mL). Cultures were induced at OD600 ~0.6 with isopropyl β-D-thiogalactopyranoside (IPTG) (0.8 mM) and cultured for 3 h at 37 °C before harvesting and flash freezing.

Cell pellets were resuspended (50 mM HEPES pH 7.2, 500 mM NaCl, 20 mM imidazole, 1 mM TCEP, 1 mM PMSF, 4 uL benzonase, 10 uL lysonase, 1x Roche complete protease inhibitor mix) and sonicated at 70% for 7 cycles (2 seconds on and 1 second off for 1 minute). The cell lysate was centrifuged (40,000 *g*, 60 min, 4 °C) and the supernatant was collected. The lysate was loaded onto an equilibrated Ni-NTA resin column (50 mM HEPES, pH 7.2, 500 mM NaCl, 20 mM imidazole, 1 mM TCEP). The column was then washed with the same equilibration buffer. AcMICU1 was eluted from the column (50 mM HEPES pH 7.2, 200 mM NaCl, 400 mM imidazole, 1 mM TCEP) and EGTA was added to a final concentration of 5 mM. The protein was concentrated in a 10 kDa concentrator to ~5 mL (OD = 20, extinction coefficient = 0.715) before it was run through FPLC (HiLoad 16 600 Superdex 200, 25 mM HEPES pH 7.2, 150 mM NaCl, 1 mM TCEP, and 1 mM EGTA), and collected in fractions (final OD = 5.9). The 8xHis tag expressed with AcMICU1 was cleaved by an in-house poly-His-tagged TEV protease with a ratio of 1:10 AcMICU1:TEV protease in equilibration buffer (2 h, 30 °C). The reaction mixture was loaded onto an equilibrated Ni-NTA column, washed with equilibration buffer, and eluted with elution buffer (4 °C). Cleaved AcMICU1 was concentrated using a 30 kDa concentrator to 500 uL and frozen in liquid nitrogen. As a quality control, SDS-PAGE was run on the reaction mixture after 1 h, the flowthrough, and the elution, and stained with Coomassie Blue (Abcam Cat# ab119211). Using standard immunoblot methods, an immunoblot with an anti-His antibody (Cell Signaling Cat# 2365S) was performed on the TEV cleavage elution to verify cleavage of the His tag.

The purified protein was sent to Cocalico Biologicals as an antigen for custom antibody production in rabbits, from which crude serum was received. Antibodies were isolated from the crude serum with a Protein A kit (Thermo Cat# 89960) according to standard protocol, resuspended 1:1 with 50% glycerol, and stored at −20 °C.

##### Mitochondrial proteomics

###### Preparation of samples for MS-based proteomics

Whole cell pellets and pellets of mitochondria of increasing purity were lysed in SDS lysis buffer (5% SDS, 50 mM TEAB pH 8.5, 2 mM MgCl_2_ 2 ug/mL aprotinin, 10 ug/mL leupeptin, 1 mM PMSF) and incubated on top of ice. 3 μL 250 units/μL benzonase (Thomas Scientific, E1014–25KU) were then added to each sample to shear DNA and samples were incubated at room temperature. Lysates were clarified by centrifugation (20,000 *g*, 10 min) and the supernatant was retained. BCA protein assay was used to estimate protein concentration. Disulfide bonds were reduced by addition of DTT to a final concentration of 5 mM and incubation at 25 °C with shaking at 1,000 rpm. Cysteine residues were alkylated with 10 mM IAA in the dark at 25 °C with shaking at 1,000 rpm. Lysates were acidified by adding phosphoric acid to 1.2% v/v.

Acidified lysates were precipitated with 6 times the sample volume of ice-cold S-Trap buffer (90% methanol, 100 mM TEAB), transferred to S-Trap cartridges and centrifuged (4,000 *g*). Precipitated proteins were washed 4 times with S-Trap buffer, centrifuged (4,000 *g*) and reconstituted in digestion buffer (50 mM TEAB with trypsin and LysC, each at 1:50 enzyme:substrate w/w ratio). After overnight digestion, peptides were eluted from the S-Trap, first with 50 mM TEAB, next with 0.1% FA and finally with 500 μL 50% ACN/0.1% FA. Eluates were snap frozen and vacuum-centrifuged until dry.

Dry digested peptides were reconstituted in 1% FA and desalted with 40 mg Sep-Pak tC18 96-well plate (Waters, SKU:186002320). Briefly, C18 wells were conditioned sequentially with 100% ACN, 50% ACN/0.1% FA, and 4x 0.1% FA. Peptides were loaded onto the C18 wells and washed with 3x with 0.1% FA, followed by 1% FA. Desalted peptides were then eluted with 2 steps of 50% ACN/ 0.1% FA, snap-frozen, and vacuum-centrifuged until completely dry. Dry desalted peptides were then reconstituted in 30% ACN/0.1% FA and quantified via BCA assay.

###### LC-MS/MS analysis

Peptide samples were reconstituted in 3% ACN/0.1% FA for LC-MS/MS analysis. Experiments were run on an Orbitrap Astral mass spectrometer (Thermo scientific) coupled to a Vanquish Neo UHPLC system (Thermo Scientific). 500 ng peptides from each sample were injected onto a PepSep C18 column (Bruker Daltonics, 25 cm length x 75 μm inner diameter, 1.5 μm particular size) with a flow rate of 300 nL/min. Samples were run on a linear 47.5 minute gradient from 4.6% solvent B (99.9% ACN, 0.1% FA) to 24% solvent B before ramping up to 48% solvent B over 4 minutes. To wash the column, solvent B was increased to 72% for 7.5 minutes. MS1 spectra were measured with a resolution of 240,000 at 200 m/z and a mass range from 300 to 1,800 m/z. The maximum injection time for MS1 was set at 5 ms and with an AGC target of 2E6. MS1 spectra were acquired with a timed loop control of 0.6 s. MS2 scans covered a mass range of 380–980 m/z with an isolation window of 4 Th. The maximum injection time was set to 5 ms for DIA scans, with an AGC target of 5E5. Precursors were fragmented with higher-energy collisional dissociation (HCD) with a normalized collision energy (NCE) set to 27%.

Mitochondrial protein correlation profiling experiments were run on a timsTOF HT mass spectrometer (Bruker) coupled to a NanoElute 2 HPLC (Bruker), equipped with a PepSep Advanced column (Bruker) and analyzed using a 28 minute gradient. DIA-PASEF analysis is performed with ion mobility range of 0.70 1/K0 [V-s/cm-2] to 1.45 1/K0 [V-s/cm-2, ramp and accumulation time of 70ms. DIA windows range from 330 m/z to 1523 m/z with 36 variable isolation windows and a cycle time of 1.67 s. The collision energy was ramped as a function of increasing mobility starting from 30 eV at 0.8 1/K0 [V-s/cm-2] to 55 eV at 1.6 1/K0 [V-s/cm-2].

###### Mass spectrometry data processing

MS spectra were searched using Spectronaut 19. Data were searched against the *Acanthamoeba castellanii* (ACANB^50^) proteome in directDIA mode using quantification settings with a precursor Q-value cutoff of 0.01, a precursor PEP cutoff of 0.2, protein Q-value cutoff (experiment) of 0.01, a protein Q-value cutoff (run) of 0.05, and a protein PEP cutoff of 0.75. The MS/MS search included the fixed modification of Carbamidomethyl (C) and variable modifications, Oxidation (M), Acetyl (Protein N-term), Deamidation (N), and FormylMet (Protein N-term), with a maximum variable modification count of 5.

Column sum normalization was applied across samples (normalized_intensityg,s = scalingFactor * intensityg,s /columnSums, for protein group *g* and sample *s*, where columnSums is the sum of intensities of sample *s*, and scalingFactor = mean(columnSums for all *s*). Next, protein groups were expanded such that normalized_intensity*p,s* = normalized_intensity*g,s* for all proteins *p* in protein group *g*. Normalized intensities were log_10_-transformed and then MinProbAll imputation was performed (missing values were replaced with values drawn from a Gaussian with mean set to the first percentile of values across all samples, and sigma set to the median of standard deviations of each protein across all samples).

###### Protein correlation profiling

Z-scores were computed for each protein across all samples. We performed imputation and Z-score analysis separately for the full dataset (24 samples) and a subset of TOM7 transfected samples (15 samples). PCP_score was calculated as the Pearson correlation between the Z-score profile for each protein versus the mean of three mitochondrial markers (TOM40 = ACANB12271; ISCU = ACANB10723; HSPD1 = ACANB13112) for the 15 TOM7 transfected samples. Genes with PCP_score ≥0.6 were labeled “MitoViaPCP”, with threshold 0.6 selected to maximize area under the precision-recall curve based on benchmark data (resulting in an estimated 8% FDR).

###### Systematic gene feature annotations

Protein sequences from ACANB^50^ were annotated with targeting signals (TargetP2.0),^61^ protein domains (HMMER version 3.1 using the Pfam-A database version 35),^146^ transmembrane helices (TMHMM version 2.0),^147^ DeepLoc2.1,^60^ and homology (BLASTP^148^ version 2.2.30+) to *Homo sapiens* (RefSeq release 63, using longest transcript per Entrez gene), *Saccharomyces cerevisiae S288C* (downloaded from *Saccharomyces* Genome Database (SGD)^149^ on 2023-05-22), *Dictyostelium discoideum* (downloaded from dictyBase^48^ on 2022-10-27), *Entamoeba histolytica* (downloaded from UniProt), *Acanthamoeba castellanii Neff* (“UniProt” protein sequences downloaded from UniProt on 2026-05-08, taxonomy=1257118; AmoebaDB57 downloaded from AmoebaDB version 57).

###### Benchmark protein lists

“Gold positive” mitochondrial *Acanthamoeba* proteins were created based on orthology to two model organisms with well-defined mitochondrial inventories: *Homo sapiens* and *Saccharomyces cerevisiae*. *Acanthamoeba* orthologs to each species were defined based on best bidirectional BLASTP best hits (expect < 1e–3). Human genes were partitioned into sets: Hmito (n = 1,137 genes in MitoCarta3.0^13^) or Hnon-mito (n = 18,111 all other human genes). Yeast genes were partitioned into sets: Ymito (n = 900 mitochondrial genes in Morgenstern et al,^14^ Ypossible-mito (n = 381 genes with mitochondrial GOslim assignment in SGD as of 2023-08-21, excluding Ymito genes), Ynon-mito (n = 4,753 all other genes). *Acanthamoeba* genes were partitioned into mitochondrial “gold+” (n = 296, orthologs to human Hmito and yeast Ymito genes, or encoded on the mtDNA), “possible_mito” (n = 3,169, homologs to human Hmito or yeast Ymito or yeast Ypossible_mito or DeepLoc2.1^60^ mitochondrial prediction score ≥ 0.4), “other“ (n = 12,479, all other genes, which we use as “gold–” for benchmarking), in that assignment order. Extrapolating from human MitoCarta3.0,^13^ we define a prior probability that 6% of *Acanthamoeba* genes encode mitochondrial proteins – and we note that mitochondria comprise an estimated 6% of the *Acanthamoeba* WT cells based on two-dimensional TEM images (see Figure 1). Based on this prior, we estimate “gold+” represents 31% of true mitochondrial proteins (0.31 = 296/(0.06*15,944)) and “gold−“ represents 83% of true non-mitochondrial proteins (0.83 = 12,479/(0.94*15,944)) – thus it is imperative to correct for the sizes of our benchmark sets when estimating false discovery rates. We benchmarked dataset accuracy using “gold+” and “gold−” as described in MitoCarta2.0^12^: true positives (TP), true negatives (TN), false positives (FP), false negatives (FN), sensitivity SN = TP / (TP + FN), specificity SP = TN / (TN + FP), corrected false discovery rate cFDR = (1 – SP)/(1 – SP + SN x *Oprior*), and Precision = 1 – cFDR, with *Oprior* = 0.06 prior probability of mitochondrial localization.

###### Comparison to prior mitoproteomics

Accuracy of the Gawryluk and colleagues 2014 mass spectrometry-based experimental mitoproteome^33^ was assessed using downloaded table S1 from that paper, with the 872 proteins detected in enriched mitochondria mapped to ACANB identifiers via peptide sequences with unique matches (829 proteins mapped), although identical sensitivity and precision was observed when mapping was performed at the protein level based on BLASTP bidirectional best hit (Expect < 1e-3).

To compare AcMitoCarta to the final mitoproteome list from Gawryluk et al ^33^, we downloaded their supplemental materials that contained protein sequences corresponding to their final experimental mitochondrial proteins (n = 709, “N#” and “M#” identifiers), experimental proteins called contaminants (n = 165, “C#” identifiers), and predicted mitochondrial proteins (n = 324 “B#” identifiers) (total of 1,198 proteins, with B282a and B282b counted once as B282). The custom Gawryluk protein IDs and their sequences are based multiple genomic assemblies (BioProject PRJNA66753 and BioProject PRJNA20303), and we found nine pairs of sequences to be identical (B306|B311, B239|N087A, B098|N607, B281|N418, B174|N290, B085|N373, B142|N365, B093|B099, B079|B190). We mapped the Gawryluk protein sequences to ACANB protein sequences via BlastP Expect < 0.001. The 1,164 IDs that we could confidently map are included as a column in Table S2. The 34 Gawryluk IDs that could not be confidently mapped to ACANB IDs include nine sequence duplicates listed above, nine proteins that failed to map likely due to differences in both genome sequence and annotation (B273, B307, B308, B317, C129, N494, N532, N564, N627), and 16 additional proteins that lacked bidirectional best hits (BBHs). Of the final mitoproteome from Gawryluk and colleagues (n = 1,033, including 324 predictions and 709 with experimental evidence, after manual removal of 165 predicted contaminants), 1,003 were found to be a BBH with an ACANB ID and were used as a foundation for the analysis shown in Figure 2.

##### Confocal immunofluorescence microscopy

###### Selection of microscopy targets

To test the localization of proteins in the MitoViaPCP list, 76 proteins across the full range of MitoViaPCP scores (1.0–0.6) were selected for HA-tagging and confocal immunofluorescence microscopy. These MitoViaPCP list targets included: (i) primarily uncharacterized proteins with no predicted localization and (ii) a smaller number of bidirectional best hits to human MitoCarta3.0 proteins, gold+ mitochondrial protein predictions, and predicted gold negative proteins found in the MitoViaPCP list.

Outside of the MitoViaPCP list, we selected 22 additional possible mitochondrial candidates for tagging and microscopy, prioritizing candidates using the following criteria: (i) MitoViaPCP score <0.6, and (ii) not mtDNA-encoded, and (iii) poorly measured in MitoIP proteomics (mean correlation across replicates <0.7), and (iv) either gold+ or predicted mitochondrial via homology and mitochondrial targeting sequence.

###### Cultures for microscopy

Cells were cultured in an 8-well chamber slide system (Nunc^™^ Lab-Tek^™^ II CC2^™^; Thermo Cat# 154941PK), seeded with 1x10^4 cells in standard medium (200–300 uL per well). Cultures were typically incubated under normoxia (ambient air) (30 °C, stationary dry incubator) for 12–24 hours. In some cases (*e.g.*, for the HydA-HA construct), to increase the abundance of proteins induced by anoxia, cultures were incubated under anoxia (Coy glovebox, 2.5% H_2_, 97.5% N_2_) for 12–24 hours and then re-oxygenated for 10–30 minutes under ambient air to allow the cells to re-adhere prior to fixation.

###### Fixation and immunostaining

The culture medium was aspirated and the cells were immediately fixed with formaldehyde (4%) in PBS (150 uL, 22 °C, 30 min). The fixative was aspirated and the cells were washed with PBS (150 uL, 22 °C, 1 min) and then 1% BSA in PBS (150 uL, 22 °C, 1 min). The cells were permeabilized with 0.1% (w/v) Triton-X-100 in PBS (150 uL, 22 °C, 5 min). The detergent solution was aspirated and replaced with 1% BSA in PBS (150 uL) (blocking solution) and incubated (1 h, 22 °C). The blocking solution was aspirated, replaced with primary antibody solution (*e.g.,* Anti-HA-AF488 (mouse, 1:400, v/v) and anti-AcMICU1 (rabbit, 1:1000, v/v), diluted in PBS with BSA (1%, w/v)) and incubated (150 uL/well, gentle rocking protected from light, 1 h, rt). The cells were washed with 1% BSA in PBS (150 uL, ~10 min, 22 °C) three times. The wash solution was aspirated, replaced with secondary antibody solution (*e.g.*, 1:1000 (v/v) dilution of goat anti-Rabbit-AF647 conjugate, diluted in PBS with BSA (1%, w/v)), and incubated (150 uL/well, gentle rocking protected from light, 1 h, 22 °C). The cells were washed with 1% BSA in PBS (150 uL, ~10 min) three times. The slides were mounted with Prolong Glass Antifade Mountant with NucBlue (Thermo Cat# P36981) and a glass coverslip and then stored in the dark at room temp to cure overnight.

###### Image acquisition and processing

Microscopy was conducted with a Nikon Eclipse Ti2, Nikon AX, DUX-ST equipped with laser lines at 405, 488, 561, and 640 nm. Z-series images (stacks of ~10 images separated by ~0.1 um) were acquired. After acquisition, Nikon 3D deconvolution was applied to the Z-series. Images were corrected for background using FIJI (Image J) software, from which TIFF image files of a single plane of the Z-series were exported.

##### Data integration to generate the MitoCarta

The *Acanthamoeba* MitoCarta (AcMitoCarta) (n = 1,122) is the union of three lists: (1) MitoViaPCP (n = 1,084, experimentally defined, mass spectrometry-based mitochondrial proteome inventory, detailed above), (2) MitoViaMicroscopy (proteins found to be mitochondrial by confocal immunofluorescence microscopy, as detailed above) (n = 96), and (3) mtDNA-encoded proteins (n = 42).

###### Manual gene names and annotations

For the AcMitoCarta inventory (n = 1,122), gene names and descriptions were assigned manually based on integration of available evidence including direct biochemical evidence (mitochondrial proteomics, CryoEM, BN-PAGE-MS, IP-MS, in vitro biochemical assays) and inference via homology (BLASTP against human and yeast, and MitoTOL orthogroups^59^), as detailed by evidence codes in the supplementary tables. For example, gene names were assigned based on homology and structural biology (CryoEM) (Fry et al^85^) (OXPHOS complexes V and III, and Hsp60). Over 600 AcMitoCarta gene names were assigned because they were either bidirectional best hits (BBH) via BLASTP with known human MitoCarta3.0 genes or in orthogroups shared with human MitoCarta3.0 genes (plus BN-PAGE-MS, IP-MS, and/or structural biology evidence for a subset). 53 AcMitoCarta gene names were assigned primarily based on membership in orthogroups shared with yeast mitochondrial proteins (plus BN-PAGE-MS evidence for ten of these). 388 genes were described as “uncharacterized mitochondrial protein” and were not assigned new names (other than their systematic ACANB gene ID from Stefely et al^50^) because they lacked clear homologs with well-characterized mitochondrial proteins in other key organisms (*e.g.*, humans and yeast).

##### Gene ancestry bioinformatics

###### Homology across species

Homology of *Acanthamoeba* proteins to 203 eukaryotic species, 29 bacterial clades (grouped at superfamily-level), and five archaeal clades (grouped at kingdom-level) was determined based on orthogroups that were inferred by OrthoFinder^150^ (v2.5.5) and refined using a custom pipeline incorporating profile Hidden Markov Model and AlphaFold^109^ structure alignments (Chen et al^59^; also see michaelzhuchen GitHub).

###### Species tree

The species tree used for visualizations was inferred from a concatenated alignment of universal ribosomal proteins across eukaryotes, bacteria, and archaea using a manually-refined NCBI taxonomy as a guide tree (Chen et al^59^; also see michaelzhuchen GitHub).

###### Determination of prokaryotic vs eukaryotic gene ancestry

The putative ancestry of each protein was assigned based on the presence/absence of its orthogroup across species. First, proteins with homologs in at least two prokaryotic clades were labeled as “Prokaryotic”. Second, proteins with homologs in at least two eukaryotic superfamilies (requiring homologs in at least two species within each superfamily) and in orthogroups containing >90% eukaryotic species were labeled as “Eukaryotic”. Third, proteins with no identified homologs beyond Amoebozoa were labeled as “Lineage-specific”. Lastly, any proteins that did not satisfy the criteria for these labels was labeled as “Indeterminate”.

###### Protein phylogeny inference

Protein phylogenies were inferred for each orthogroup of interest using a custom pipeline. Briefly, for each orthogroup, we created a multiple sequence alignment of the orthogroup member proteins, inferred initial trees by maximum-likelihood, rooted the initial trees using a prokaryotic outgroup and/or minimum ancestor deviation, reconciled the rooted trees with the species tree using the AleRax reconciliation algorithm, and then inferred branch lengths for the resulting reconciled consensus phylogeny (for methodological details see Chen et al^59^; also see michaelzhuchen GitHub). The reconciled consensus phylogeny was then annotated with characterized homologs from the literature.

###### Determination of horizontal gene transfer

Prokaryote-derived HGT into the whole proteome of *Acanthamoeba* was inferred by phylogenetic analysis. Phylogenetically resolved orthogroups (PhROGs) that contained any *Acanthamoeba* protein and a prokaryotic homolog were subjected to the protein phylogeny inference procedure described above. The reconciled protein phylogenies were screened for candidate prokaryote-derived HGT, defined as a well-supported (reconciliation conditional clade probability of at least 0.5), eukaryotic (containing at least two-fold enrichment of eukaryotic species relative to all species in the dataset) clade that had non-eukaryotic sister and cousin clades. The timing of each transfer was assigned based on the eukaryotic species in the clade, filtering for *Acanthamoeba*, Amoebozoa, or Amorphea timings to identify *Acanthamoeba* proteins that were acquired by relatively recent HGT. The putative prokaryotic donor group was assigned based on the prokaryotic group in the sister clade that had the highest enrichment score, defined as the fraction of prokaryotic group species found in the sister clade relative to the total number of species in the prokaryotic group. HGT annotations are included as columns in Table S2.

###### Anaerobic enrichment screen in prokaryotes

In brief, prokaryotic oxygen phenotypes were retrieved from publicly available databases and used to identify orthogroups that were enriched in anaerobic versus aerobic prokaryotic members. Detailed methodology is described in Chen et al^59^.

##### HupA Biochemistry

###### Purification of recombinant *Acanthamoeba* HupA protein

AcHupA (ACANB05819) was cloned into a pET28a(+) vector with an N-terminal 6xHis tag and SUMO fusion protein. AcHupA-R66E mutant was generated via site-directed mutagenesis. Both wild-type and mutant AcHupA were expressed in NiCo21(DE3) *E. coli* grown in 2×YT Broth. Cultures were induced at OD_600_ ~0.6 with IPTG (0.5 mM) and grown overnight at 18 °C before harvesting and flash freezing.

Cell pellets were resuspended in Buffer A (50 mM HEPES, pH 7.9, 300 mM NaCl, 10 mM imidazole, 10 mM β-mercaptoethanol) with DNase I and protease inhibitors (PMSF, benzamidine), homogenized, and lysed using a cell disruptor. Lysates were centrifuged to separate the soluble fraction, which was incubated with 3 mL pre-equilibrated Ni-NTA agarose (Qiagen) (1.5 h, 4 °C, rotating). Resin was transferred to a gravity column, and flow-through was re-applied before washing with 75 mL Buffer A. Protein was eluted with 10 mL Buffer B (50 mM HEPES pH 7.9, 300 mM NaCl, 300 mM imidazole, 10 mM β-ME). The His-SUMO tag was cleaved via incubation ULP1 for 1 h at 4 °C, then the solution was passed through a 0.2 μm filter and loaded onto an ÄKTA FPLC. Tag-free protein was separated from ULP1 and SUMO by cation exchange chromatography (HiTrap SP, 2×1 mL) ranging from low salt (50 mM HEPES pH 7.9) to high salt (50 mM HEPES, pH 7.9, 1 M NaCl). The protein eluted between 600–800 mM NaCl, was quantified by NanoDrop, and flash frozen at 100 μM for downstream assays.

###### Electrophoretic mobility shift assay

Purified WT AcHupA or R66E AcHupA protein was first diluted in a working buffer (20 nM pUC19 plasmid, 50 mM MgCl_2_, 100 mM KCl) to variable concentrations on ice, with a final reaction volume of 40 μL. Each reaction was incubated at RT for 30 min, then 10 μL of each reaction was mixed with 1 μL 80% glycerol. The samples were run on a 1% agarose gel containing 1X GelRed (Biotium Cat #41003) (110 V, 30 min) and imaged using a Biorad ChemiDoc MP Imager.

##### Mitochondrial Complexomics

###### Blue native PAGE for mitochondrial complexomics

*Acanthamoeba* mitochondria pellets were isolated by mitochondrial immunoprecipitation (MitoIP) as described above, then snap frozen in N_2(l)_ and stored at −80 °C. Two 25 μg samples were prepared: one sample solubilized in digitonin to a detergent:protein ratio (m/m) of 6.7, and the other solubilized in n-dodecyl-β-d-maltoside (DDM) to a detergent:protein ratio (m/m) of 2. For each sample at 4 °C on ice, NativePAGE 4x Sample Buffer (5 uL, Thermo Cat# BN2007), 5% digitonin (4 uL) or 5% DDM (1.2 uL) (Thermo Cat#BN2008), Benzonase (Sigma Cat#E8263-5KU, 50 units/uL, 0.8 μL), MgCl_2_ (50 mM, 0.8 μL), and dH_2_O were added to mitochondria pellet resuspended in dH_2_O (5.0 mg/mL, 5 μL), to a final volume of 20 μL and an initial mitochondrial mass of 30 ug. The samples were incubated on top of ice (4 °C, 20 min) and then centrifuged (20,000 *g*, 10 min, 4 °C). The supernatant (20 μL) was transferred into a new tube on ice and NativePAGE 5% G-250 Sample Additive (Thermo Cat# BN2007) (volume equal to ¼ of the detergent concentration) was added. Two control samples with bovine heart mitochondria (5.5 mg/mL, Abcam Cat. #110338) solubilized in either digitonin or DDM were also prepared according to the parameters above.

The electrophoresis system (XCell SureLockMini-Cell) was set up on ice and a NativePAGE Novex 3% to 12% Bis-Tris protein gel (Thermo Cat. #BN2011BX10) was placed inside. Dark Blue Cathode Buffer (1 mL, 4 °C) (10 mL 20X NativePAGE Running Buffer, 10 mL 20X NativePAGE Cathode Additive (Thermo Cat# BN2007), 180 mL dH_2_O) was gently pipetted inside the gel wells to wash, and an additional 20 mL was poured into the inner chamber of the gel box for leak inspection. The electrophoresis system was placed on ice and moved into the cold room. NativeMark Unstained Protein Standard (7 μL, Thermo Cat# LC0725) and the samples (16.7 μL for a final input of 25 μg) were loaded into the gel wells, avoiding bubble formation. Dark Blue Cathode Buffer was added to the inner chamber until full and Anode Buffer (600 mL, 4 °C) (50 mL 20X NativePAGE Running Buffer, 950 mL dH_2_O) was added to the outside chamber. The gel was run on ice (150 V, 30 min, 4 °C) The run was paused, and the Dark Blue Cathode buffer was carefully removed and replaced with Light Blue Cathode Buffer (200 mL, 4 °C) (10 mL 20X NativePAGE Running Buffer, 1 mL 20X NativePAGE Cathode Additive, 189 mL dH_2_O). The gel run was resumed (250 V, 150 min, 4 °C), still on ice.

The gel was fixed in Fixation Solution (40% (v/v) methanol, 10% (v/v) acetic acid, 10 mM ammonium acetate) (30 min, RT, gentle rocking). The gel was de-stained in Destaining Solution (10% (v/v) acetic acid) (3x 20 min, RT, gentle rocking) until the background became clear and the NativeMark Unstained Protein Standard could be visualized. The gel was washed in dH_2_O (3x 5 min, RT, gentle rocking) and digitally imaged with an Amersham Imager 680.

A custom cutting template of 60 slices, upon which the gel could be directly superimposed, was prepared digitally and printed. A 96-well filtered microtiter plate was activated (100 uL, 100% acetonitrile) and spun (1,500 *g*, room temp). The plate was washed (100 μL 50 mM ammonium bicarbonate prepared in LC-MS grade H_2_O) and spun (1,500 *g*, room temp) three times. Then the wells were filled (150 μL, 50 mM ammonium bicarbonate), covered, and placed on ice. The gel was laid out on a cutting board over the template. The gel lanes of interest were carefully cut out with a straight edge razor blade. The gel lanes were slid apart and separately sliced into five major sections each, for separate processing starting with the fragile, topmost section of the gel. A scalpel was used to carefully cut a pre-defined gel band of interest by cutting horizontally from one side of the gel to the other, starting from the bottom of the section. After each band was removed, the band was further cut into 2–3 cubes, then carefully transferred into the corresponding well of the 96-well plate with tweezers. The scalpel and tweezers were washed with methanol (LC-MS grade) and water (LC-MS grade) to avoid contamination. The gel band processing was repeated for all 60 bands of the gel. After the 96-well plate was fully loaded, it was stored at −80 °C prior to further processing of the gel slices.

###### BN-PAGE OXPHOS in-gel enzyme activity assays

BN-PAGE was run on *Acanthamoeba* MitoIP pellets (described above) and bovine heart mitochondria pellets (Abcam Cat. #110338), solubilized in 1% (w/v) digitonin, as described in the previous section. A lane with the NativeMark Unstained Protein Standard was also included. At the end of the gel run, the gel was briefly washed in ice-cold dH_2_O, then divided into the molecular weight ladder and gel lanes for assessing OXPHOS activity. All activity solutions were prepared fresh, and all gels were scanned with an Amersham Imager 680.

To assess Complex I activity, the gel was incubated in 10 mL “Complex I” (NADH dehydrogenase) activity solution (3 mM iodonitrotetrazolium chloride, 1 mM NADH, 5 mM Tris pH 7.5) (7 min, RT, gentle rocking) until red bands appeared, then incubated with 10% acetic acid to stop activity.

To assess Complex II activity, the gel was incubated in 10 mL Complex II activity solution (20 mM succinate, 0.2 mM PMSF, 3 mM nitrotetrazolium blue, 5 mM Tris pH 7.5) (1 h, RT, gentle rocking) until violet bands appeared, then incubated with 10% acetic acid to stop activity.

To assess Complex IV activity, the gel was incubated with 10 mL Complex IV activity solution (0.05 mM cytochrome c, 2.3 mM diaminobenzidine, 50 mM sodium phosphate pH 7.2) (30 min, RT, gentle rocking) until brown bands appeared, then incubated with 10% acetic acid to stop activity.

To assess Complex V activity, the gel was incubated with 10 mL Complex V activity solution (14 mM magnesium sulfate, 0.2% (w/v) lead nitrate, 8 mM adenosine triphosphate (ATP), 270 mM glycine, 25 mM Tris pH 8.4) (16 h or overnight, RT, gentle rocking), the incubated with 50% methanol (10 min) to stop activity.

To visualize the molecular weight ladder, the gel lane was stained with Coomassie Brilliant Blue R-250 stain solution (0.1% (w/v) Coomassie Brilliant Blue R-250, 25% (v/v) isopropanol, 10% (v/v) acetic acid) (30 min, RT, gentle rocking), then destained with 10% acetic acid until the ladder can be visualized (3–4 h, RT, or overnight, 4 °C, gentle rocking).

##### BN-PAGE gel slice processing and LC-MS/MS analysis

Gel bands were destained by incubating with 100 μL of 50% acetonitrile (ACN) at 400 rpm for 15 min at room temperature. The solution was removed, and bands were washed with 100 μL of 50 mM ammonium bicarbonate (AB) under the same conditions. This destaining and washing cycle was repeated three times until gel bands were completely clear. For protein reduction and alkylation, samples were treated with 50 μL of 10 mM dithiothreitol (DTT) at 400 rpm for 45 min at 37 °C, followed by carbamidomethylation using 50 μL of 55 mM iodoacetamide in the dark for 1 h at room temperature. Samples were then washed sequentially with 50% ACN and AB as described above. Gel bands were dehydrated with 100 μL of 100% ACN for 10 min at room temperature with shaking. After removing the ACN, gel pieces were rehydrated with 10 μL of trypsin (Promega, 5 ng/μL) and incubated at 37 °C for 5 min. An additional 50 μL of AB was added to cover the gel pieces, and proteolytic digestion was carried out overnight at 37 °C with shaking at 600 rpm. The following day, supernatants were transferred to micronic vials (MPW42072LBC3, 0.5 mL, MicroTrade and 4466BLU, Thermo Scientific). Peptides were extracted from gel pieces through sequential incubations: first with 50 μL HPLC-grade water (400 rpm, 20 min, room temperature), then with 50 μL of 30% ACN/0.1% formic acid (FA) under the same conditions, and finally with 50 μL of 70% ACN/0.1% FA. All supernatants were pooled, frozen, and lyophilized for subsequent analysis.

Prior to LC-MS/MS analysis, samples were reconstituted in 45 μL of 0.1%FA and 20 μL were loaded on Evotip (EV2011, Evosep Biosystems) following standard procedure. Samples were run on an Evosep One (Evosep Biosystems) connected to a Q Exactive^™^ Plus Hybrid Quadrupole-Orbitrap^™^ Mass Spectrometer (Thermo). Peptides were separated on an Endurance Column (15 cm × 0.15 mm, 1.9 μm, EV1106, Evosep Biosystems) using the standard 30SPD method.

Ions were analyzed in data-dependent mode with an MS1 scan range of 300–1,800 m/z at 70,000 resolution. The automatic gain control (AGC) target was set to 3E6 ions and the maximum injection time was set to 50 ms. For each MS1 scan, the top 12 most abundant peaks were picked for fragmentation using a collision energy of 25 and isolation window of 1.7 m/z. MS2 spectra were acquired in centroid mode at a resolution of 17,500 with an AGC target of 5E4 and maximum injection time of 100 ms. Dynamic exclusion was set to 10 s. Ions were excluded if the charge was unassigned, equal to 1 or greater than 6.

###### BN-PAGE-MS data processing

Mass spectrometry data were processed using Spectrum Mill (Broad Institute). Extraction of raw files retained spectra within a precursor mass range of 600–6,000 Da and a minimum MS1 signal-to-noise ratio of 25. MS1 spectra were merged if they were within a retention time range of +/− 45 s, or within a precursor m/z tolerance of +/− 1.4 m/z. MS/MS searching was performed against the ACANB proteome database (Stefely et al^50^). Digestion parameters were set to “trypsin allow P” with an allowance of 4 missed cleavages. The MS/MS search included fixed modification of carbamidomethylation on cysteine. Variable modifications were acetylation of the protein N-terminus, oxidation of methionine, conversion of glutamine to pyro-glutamic acid, deamidation of asparagine and conversion of carbamidomethylated cysteines to pyrocarbamidomethyl cysteines. Both parent and fragment mass tolerances were set at 20 ppm. Peptide matches were validated using a maximum FDR threshold of 1%.

Of note, *Acanthamoeba* has some genes that appear to be recent duplications. These paralogs are often different at the level of their DNA and RNA sequences but may have identical or highly similar protein amino acid sequences. In this BN-PAGE-MS dataset where each row represents a different gene, these groups of proteins with identical amino acid sequences are effectively displayed twice, as they are indistinguishable at the level of the peptides observed by mass spectrometry proteomics.

###### BN–PAGE-MS data analysis

Protein groups were expanded such that all proteins in the protein group receiving the same top score (thus indistinguishable peptide evidence) were considered as detected. Relative abundance was calculated for each protein as intensity value / Max intensity value observed across any slice 1–60 (calculated separately for Digitonin and DDM datasets). Heatmaps in Figure 3B are limited to AcMitoCarta proteins and were generated in Excel using the gene-based data in Table S4C. AcMitoCarta proteins in orthogroups with mitochondrial proteins in either humans or yeast are colored blue, and proteins in orthogroups lacking such human or yeast mitochondrial proteins are termed “unique” and colored green. Complexes were sorted by peak intensity and slice of peak intensity. To identify co-migration candidates for each known protein complex, we (i) manually identified “bait” proteins (with homology to human or yeast complex subunits) that showed a coherent profile in both Digitonin and DDM, (ii) created a “bait” profile as the average of these bait proteins in the 60 Digitonin slices and separately in the 60 DDM slices, (iii) scored all other proteins using Pearson correlation to the “bait” profile to each experiment, (iv) manually reviewed high-scoring proteins as potential co-migration hits. Figure 3C shows observed co-migrating members of identified complexes (complexes sorted by slice of peak intensity). Figure 3D was generated in R at the protein group level, using iBAQ values for the first protein per protein group (unlike the rest of Figure 3 which shows relative abundance per protein calculated using intensity rather than iBAQ).

##### Immunoprecipitation-mass spectrometry (IP-MS)

Crude mitochondrial pellets from *Acanthamoeba* cells transfected with the plasmid of interest were isolated (crude mito prep methods as detailed above). Pellets were lysed in 250 μL icecold buffer (0.3% (w/v) DDM, 150 mM NaCl, 50 mM Tris pH 7.5, 1x Roche complete protease inhibitor mix, 500 μM freshly added PMSF) and left on ice for 10 min. Insoluble materials were pelleted (20,000 *g*, 10 min, 4 °C) and the protein concentration of the cleared lysate was determined using a BCA. Equal masses of cleared lysate (0.4 mg) diluted to a total volume of 0.9 mL were mixed with 50 μL pre-equilibrated anti-HA magnetic beads (Thermo Cat#88837) for 2 h at 4 °C with end-over-end mixing. After incubation, the beads were washed twice in buffer containing a small amount of DDM (0.03% (w/v) DDM, 150 mM NaCl, 50 mM Tris pH 7.5, 1x Roche complete protease inhibitor mix), then again in DDM-free buffer (150 mM NaCl 50 mM Tris pH 7.5, 1x Roche complete protease inhibitor mix). The beads were washed once with Tris-buffered saline (75 mM NaCl, 50 mM Tris pH 8) (TBS), resuspended in 50 μL TBS, then snap frozen in liquid nitrogen and stored at −80 °C.

For the HydA IP-MS set, two replicates were performed. One replicate of the IP was done according to the protocol above (under standard “normoxia” conditions). A second replicate of the IP was conducted in a glovebox anaerobic chamber (Coy Laboratory Products Inc.) (2.5% H_2(g)_, 97.5% N_2(g)_) with buffers that had been deoxygenated overnight. The results were found to be similar for both the normoxia and anoxia IPs, so they were analyzed together as shown in the figures.

Following affinity capture, magnetic beads carrying the respective *Acanthamoeba* bait proteins were washed four times with 200 μL of 50 mM Tris-HCl (pH 7.5) and then subjected to an initial on-bead digestion in 100 μL of digestion buffer (2 M urea, 50 mM Tris-HCl pH 7.5, 1 mM DTT, 0.4 μg trypsin) at 25 °C for 1 h with shaking at 1,000 rpm; the resulting eluate was collected into a clean tube. A second round of on-bead digestion was carried out by adding an additional 100 μL of the same digestion buffer and incubating under identical conditions for 30 min, and the eluate was combined with the first. Beads were then washed twice with 60 μL of urea wash buffer (2 M urea, 50 mM Tris-HCl pH 7.5), and the washes were combined with the collected supernatants. The combined eluate was reduced with 4 mM DTT at 25 °C for 30 min with shaking at 1,000 rpm and subsequently alkylated with 10 mM IAA at 25 °C for 45 min with shaking at 1,000 rpm in the dark. An additional 0.5 μg of trypsin was then added for an overnight digestion at 37 °C with shaking at 800 rpm. The following day, digests were acidified to 1% (v/v) formic acid (final pH < 3) using neat formic acid. Acidified peptides were desalted on homemade C18 StageTips and analyzed by LC-MS/MS on a Bruker timsTOF HT instrument coupled to an NanoElute 2 (Bruker). LC/MS runs and data searching was carried out as described above in the mitochondrial proteomics section. MS data was normalized and missing values were imputed as described above for the PCP data analysis.

##### Whole cell perturbation profiling

###### O_2_ perturbation profiling cultures

*Acanthamoeba* was first cultured under normoxia (21% O_2_, ambient air) in standard media as described above until it reached confluency (95–100% confluency, adherent cells). The cells were harvested, washed with encystation medium (20 mM Tris-HCl, pH 8.8, 100 mM KCl, 8 mM MgSO_4_, 0.4 mM CaCl_2_, 1 mM NaHCO_3_), and then resuspended in a small volume of encystation medium for inoculation of the experimental cultures.

A total of 30 experimental cultures were conducted, with each of 10 culture conditions in biological triplicate (separate flasks)—spanning two media conditions (standard (rich) medium or encystation (starvation) medium) and five different O_2_ perturbation atmospheric conditions: (i) ambient air (21% O_2_) for 24 hours, (ii) hypoxia (1% O_2_, 99% N_2_ mixed gas) for 24 hours, (iii) near-anoxia (100% N_2(g)_) for 24 hours, (iv) near-anoxia (100% N_2(g)_) for 24 hours followed by 1 hour of re-oxygenation under ambient air (21% O_2_), or (v) near-anoxia (100% N_2(g)_) for 24 hours followed by 6 hours of re-oxygenation under ambient air (21% O_2_). At the beginning of the experiment, sealed chambers were repeatedly flushed with pre-mixed gasses to establish defined atmospheres. O_2_ levels < 0.01% in the near-anoxia condition were confirmed using a colorimetric O_2_ detection pellet (Anaero-Indicator, Cat# 30-02, Mitsubishi Gas Chemical America, Inc). T-75 flasks (13 mL of media, final volume) were inoculated with 2x10^6 cells (normoxic standard media condition), 4x10^6 cells (all other conditions with standard media), or 10x10^6 cells (encystation media conditions), to achieve similar culture densities at the end of the experiment (based on prior growth optimization studies).

Where indicated for re-oxygenation, the cultures were removed from the sealed chamber (near-anoxic), placed in the biosafety hood, and opened (uncapped) to allow gas to quickly equilibrate with the ambient (normoxic) air (~1 min). The flasks were then re-capped and the media was agitated by shaking briefly to fully re-oxygenate the media. The cultured were then returned to the incubator (30 °C, stationary dry, ambient air) for the indicated re-oxygenation time.

Phase contrast microscopy images of each culture were captured immediately prior to harvest. Using the media already in the flask, loosely adherent cells were mechanically detached from the tissue culture flask (hitting the back of the flask 5 times and then rocking the flask back and forth 5 times). Cell detachment was completed by gently scraping (rubber scraper) for any flasks with encystation media. The media (12–13 mL, including non-adherent cells) was decanted into a 15-mL conical tube on ice with 60 uL of 100 mM PMSF stock (protease inhibitor). PBS with PMSF (2–3 mL, 4 °C) was added to the culture flask (to bring the total harvest volume to 15 mL). The detached and suspended cells were transferred to the same 15-mL conical tube as the first harvest of non-adherent cells with the decanted media. The combined cells were harvested by centrifugation (5,000 *g*, 4 °C, 5 min). The supernatant was decanted and discarded. PBS with 2x Protease Inhibitor Mix (975 uL, 4 °C) was added and the cells were resuspended. The sample was aliquoted into 1.5-mL microcentrifuge tubes on ice as follows: 475 uL for protein analysis, 475 uL for RNA analysis, ~50–100 uL for microscopy analysis. The cells were pelleted by centrifugation (10,000 *g*, 3 min, 4 °C). The supernatant was removed and discarded, and the cell pellets were further processed as follows: (i) The cell pellet for protein analysis was immediately frozen in N_2(l)_ and then stored at –80 °C. (ii) The cell pellet for RNA analysis was resuspended in TRI reagent (600 uL, 4 °C) and then stored at −80 °C. The cell pellet for electron microscopy analysis was resuspended in PBS (50 uL, 4 °C), mixed with FGP fixative (2.5% paraformaldehyde, 5% glutaraldehyde, 0.06% picric acid in 0.2 M Cacodylate buffer) (50 uL, 4 °C), pelleted by centrifugation (1,000 *g*, 4 °C, 5 min), and then stored as a pellet in this fixative solution at 4 °C, protected from light. (iv) Cell pellets for fluorescence microscopy were resuspended in 4% PFA (100 uL, 4 °C) and then stored as a pellet in this fixative solution at 4 °C, protected from light.

###### Lysis of O_2_ perturbation profiling samples for proteomics

The cell pellets were resuspended in SDS Lysis Buffer (150 uL, 5% SDS, 50 mM TEAB, 2 mM MgCl_2_, 2 ug/mL aprotinin, 10 ug/mL leupeptin, 1 mM PMSF). For the encystation media samples with mature cysts, added a 100 uL scoop of 0.5-mm glass beads and 50 uL of extra SDS Lysis Buffer to each tube. Incubated the tubes on top of ice (15 min) to start lysis. For samples with mature cysts, lysed cells by bead beating (BioSpec MiniBeadbeater operated in a cold room at 4 °C, cycles of 1 min on, 2 min off, four total cycles for a total of 4 min of bead beating time). For all samples, added 2 uL of benzonase and incubated on ice (20 min). Centrifuged (4 °C, 10 min, 18,000 *g*) to pellet insoluble debris and transferred 150–175 uL of the cleared lysate into a fresh tube on ice. Measured the protein concentration of each cleared lysate by BCA assay. Snap froze the remaining cleared lysate in N_2(l)_ and stored at −80 °C.

###### RNA isolation for O_2_ perturbation profiling transcriptomics

The samples of cells in TRI reagent were thawed on ice and resuspended by vortexing. For the samples with mature cysts, added a 100 uL scoop of 0.5-mm glass beads to each tube and lysed cells by bead beating (BioSpec MiniBeadbeater operated in a cold room at 4 °C, cycles of 1 min on, 5 min off to allow samples to cool on ice, total bead beating time of 3 min). All the other samples were lysed by simple vortexing. Centrifuged (4 °C, 5 min, 15,000 *g*) to pellet insoluble debris and transferred the cleared lysate into a fresh tube. Conducted RNA extraction per protocol (Direct-zol RNA Miniprep Plus). Added EtOH (equal volume of TRI reagent sample, 1:1, v/v, 600 uL, rt) and mixed by vortexing. Transferred the mixture into a Zymo-Spin IIICG Column in a collection tube and centrifuged (15,000 *g*, 30 s, rt). Transferred the column into a new collection tube and discarded the flow-through. Performed DNAase I treatment. Added Direct-zol RNA PreWash (400 uL) to the column, centrifuged (15,000 *g*, 30 s, rt), and discarded the flow-through. For a second time, added Direct-zol RNA PreWash (400 uL) to the column, centrifuged (15,000 *g*, 30 s, rt), and discarded the flow-through. Added RNA Wash Buffer (700 uL) to the column, centrifuged (15,000 *g*, 1 min, rt), and discarded the flow through. After the flow through was discarded, centrifuged again (15,000 *g*, 1 min, rt) to completely remove the wash buffer, and then discarded the flow through. Transferred the column carefully into an RNase-free tube. To elute RNA, added Dnase/Rnase-free water (50 uL) directly to the column matrix and centrifuged (15,000 *g*, 30 s, rt). Measured RNA concentrations by NanoDrop. Stored RNA at −80 °C.

###### Carbon and iron nutrient perturbations

*Acanthamoeba* was first cultured under normoxia (21% O_2_, ambient air) in standard media until it reached confluency (95–100% confluency, adherent cells). The cells were harvested, washed with glucose-free medium, and then resuspended in a small volume of glucose-free medium for inoculation of the experimental cultures.

A total of 18 experimental cultures were conducted, with each of six culture medium conditions in biological triplicate (separate flasks). All six culture media conditions included a consistent base composition of 20 g/L proteose peptone, 1 g/L yeast extract, 4 mM MgSO_4_, 0.4 mM CaCl_2_, 3.4 mM sodium citrate, 2.5 mM Na_2_HPO_4_, 2.5 mM KH_2_PO_4_, pH 6.5. Variable components include the carbohydrate and the iron source: (i) glucose (100 mM) and 50 uM ferrous ammonium sulfate (Fe(NH_4_)_2_(SO_4_)_2_) (“standard medium”), (ii) galactose (100 mM) and 50 uM ferrous ammonium sulfate (Fe(NH_4_)_2_(SO_4_)_2_), (iii) glycerol (100 mM) and 50 uM ferrous ammonium sulfate (Fe(NH_4_)_2_(SO_4_)_2_), (iv) no added carbohydrate and 50 uM ferrous ammonium sulfate (Fe(NH_4_)_2_(SO_4_)_2_), (v) glucose (100 mM) and no added iron compound, (vi) glucose (100 mM) and 50 uM ferric ammonium sulfate (FeNH_4_(SO_4_)_2_). The cultures were seeded into this variable media, and this variable media was refreshed 12 hours after the cultures were inoculated. The cultures were harvested when they achieved ~75% confluency, which occurred at variable time points based on differential proliferation rates in the variable media (1 day for glucose, no carbohydrate, ferric, and no-iron cultures, 2 days for the galactose cultures, and 3 days for the glycerol cultures). Samples for proteomics, transcriptomics, and microscopy were harvested and processed as detailed above for the O_2_ perturbation profiling experiment.

###### RNA-seq transcriptomics for whole cell perturbation profiling

Isolated RNA from both the O_2_ perturbation experiment and the carbon and iron nutrient perturbation experiments was submitted together for batched RNA-seq transcriptomics (150 base pair paired end reads) (Azenta).

###### RNA-seq data processing

The raw sequencing reads were processed using a custom bulk RNA-seq processing pipeline to align paired end reads to the 2021 release of the *Acanthamoeba castellanii* str. Neff genome sequence and the ACANB genome annotation^50^ (STAR – outSAMattributes NH HI NM MD AS XS nM, and –twopassMode Basic), remove duplicate reads (Picard 2.27.5 MarkDuplicates tool^151,152^, as distributed in GATK v 4.3.0.0), assign reads to genes (featureCounts v 2.0.6^153^ options -t exon -p, -M, --countReadPairs, and –primary), create BAM files (SAMTools v 1.6^154^), and aggregate read counts per gene (featureCounts) into a single read count matrix. The raw count matrix was joined with the metadata about the samples and genes using AnnData (v 0.8.0)^155^, and Scanpy (v 1.9.3)^156^ was used to conduct a principal components analysis (PCA). The raw counts were first normalized using transcripts per million (TPM), then log-scaled (scanpy.pp.log1p). Highly variable genes were identified (scanpy.pp.highly_variable_genes min_mean = 0.5, max_mean = 8, min_disp = 1.0).

###### Preparation of whole cell samples for perturbation profiling mass spectrometry

Whole cell pellets were processed for mass spectrometry. Samples were lysed in SDS lysis buffer (5% SDS, 50 mM TEAB pH 8.5, 2 mM MgCl_2_, 2 μg/mL Aprotinin, 10 μg/mL Leupeptin, 1 mM PMSF, disrupted by gentle vortexing, and incubated at room temperature for 15 min. 3 μL 250 units/μL benzonase (Thomas Scientific, E1014-25KU) was then added to shear DNA in each sample, following by mixing and incubation for 15 min at room temperature. Sample lysates were centrifuged for 10 min at 20,000 *g* to clear, and the supernatant was transferred for S-Trap digestion. BCA protein assay was used to estimate protein concentration in the supernatant. Disulfide bonds were reduced in 5 mM DTT for 1 h at 25 °C and 1,000 rpm shaking, followed by alkylation of cysteine residues in 10 mM IAA in the dark for 45 min at 25 °C and 1,000 rpm shaking. The samples were acidified at 1:10 12% phosphoric acid:lysate (v/v).

An equivalent of 30–50 ug protein from each sample was aliquoted for S-Trap clean-up and digestion. Proteins in samples were then precipitated with 6× sample volume of ice-cold S-Trap buffer (90% methanol, 100 mM TEAB). The buffer with precipitate was thoroughly mixed before being transferred to a S-Trap Midi (Protifi) and then centrifuged at 4,000 *g* for 1 min. The precipitated proteins were washed 4 times, each with 3 mL S-Trap buffer, centrifuged at 4,000 *g* for 1 min. The proteins deposited on the S-Trap were then digested overnight with 350 μL digestion buffer (50 mM TEAB with trypsin and LysC, each at 1:50 enzyme:substrate weight:weight ratio).

Specifically, the digestion buffer was passed through each S-Trap column with 1 min centrifugation at 4,000 *g* and then added back atop of the S-Trap columns. The cartridges were left capped overnight at 25 °C. After overnight digestion, peptides were eluted from the S-Trap, first with 500 μL 50 mM TEAB and next with 500 μL 0.1% FA, each for 30 s at 1,000 *g*. The final elution of 500 μL 50% ©/0.1% FA was centrifuged for 1 min at 4,000 *g* to clear the cartridge. Eluates were snap frozen and vacuum-centrifuged until dry.

Dry digested peptides were reconstituted in 1 mL of 1% FA and desalted with 40 mg Sep-Pak tC18 96-well Plate (Waters, SKU:186002320). Briefly, C18 wells were conditioned sequentially with 1 mL of 100% ©, 1mL of 50% ©/0.1% FA, and 4x 1mL of 0.1% FA. Next, acidified peptides were loaded onto the C18 wells and washed with 3x 1mL uL of 0.1% FA, followed by 1x 1mL 1% FA. Desalted peptides were then eluted from the C18 resin using 2x 600uL of 50% ©/ 0.1% FA, snap-frozen, and vacuum-centrifuged until completely dry. Dry desalted peptides were then reconstituted in 30% ©/0.1% FA and quantified using a BCA assay.

###### LC-MS/MS DIA analysis

All peptide samples were reconstituted in 3% ©/0.1% FA for LC-MS/MS analysis on an Orbitrap Astral mass spectrometer coupled to a Vanquish Neo UHPLC system. 500 ng peptides from each sample were injected onto a PepSep C18 column (Bruker Daltonics, 25 cm length x 75 μm inner diameter, 1.5 μm particular size) with a flow rate of 300 nL/min. Samples were run on a linear 60 minute gradient from 99% solvent A (0.1% FA), 1 min 3% solvent B (99.9% ©, 0.1% FA), 46 min 22% B, 51.5 min 34% B, 52.5 min 50% B. MS1 spectra were measured with resolution of 240,000 at 200 m/z and a mass range from 300 to 1,800 m/z. The maximum injection time for MS1 was set at 5 ms and with an AGC target of 2E6. MS1 spectra were acquired with a timed loop control of 0.6 s. MS2 scans covered a mass range of 380–980 m/z with an isolation window of 4 Th. The maximum injection time was set to 5 ms for MS2 scans, with an AGC target of 5E5. Precursors were fragmented with higher-energy collisional dissociation (HCD) with a normalized collision energy (NCE) set to 27%.

###### Mass spectrometry data search and processing

MS spectra were searched using Spectronaut 19. Data were searched against the ACANB^50^ protein database in direct DIA mode using quantification settings with a precursor Q-value cutoff of 0.01, a precursor PEP cutoff of 0.2, protein Q-value cutoff (experiment) of 0.01, a protein Q-value cutoff (run) of 0.05, and a protein PEP cutoff of 0.75. The MS/MS search included the fixed modification of Carbamidomethyl © and variable modifications, Oxidation (M), Acetyl (Protein N-term), Deamidation (N), and FormylMet (Protein N-term), with a maximum variable modification count of 5. MS intensity normalization, expansion, and imputation were performed as described for the mitochondrial enrichment dataset.

##### Hydrogenase procedures

###### HydA sequence analysis

The primary amino acid sequence of *Acanthamoeba* HydA (ACANB03778_t1) was aligned using MUSCLE with that of *Clostridium pasteurianum* HydA (UniProt P29166), which has a solved x-ray crystal structure (PDB 1FEH), and a series of additional HydA homologs identified through BLASTP against the NCBI NR database (species and gene IDs listed in figure). Conserved iron-sulfur cluster metal-binding ligands of *Clostridium* HydA identified based on the x-ray structure were mapped to *Acanthamoeba* HydA residues in the sequence alignment.

###### Anaerobic isolation of mitochondria

*Acanthamoeba* was cultured in ten 182-cm side-arm flasks (30 mL standard medium/flask, 30 °C, stationary dry incubator, ambient air) until the cultures proliferated to the point of confluency (95–100% confluent, adherent cells). At this point, the spent media was decanted and replaced with fresh standard medium (30 mL, pre-warmed to 30 °C). The cultures were then moved to a glovebox anaerobic chamber (Coy Laboratory Products Inc.) and incubated (24 hours, 30 °C, dry incubator, stationary) (2.5% H_2(g)_, 97.5% N_2(g)_). At least 12 hours prior to the mitochondrial isolation procedure, all plasticware and required buffers were de-oxygenated and cooled inside of the anaerobic chamber.

The following procedure was performed primarily inside of the anaerobic chamber under strictly anoxic conditions. For brief centrifugations outside of the anoxic chamber, tubes were tightly capped and further sealed with parafilm. Working inside of the anoxic chamber, both adherent cells and non-adherent cells were harvested. Using the existing media in the flasks, the adherent cells were mechanically detached from the tissue culture flask (hitting the back of the flask 5 times, hitting the side of the flask 5 times, and then rocking the flask back and forth 5 times) and transferred to 250-mL plastic conical tubes. To consume any small amounts of O_2_, sodium hydrosulfite (DTH) was added to a final concentration of 5 mM. The harvested cells were centrifuged (800 *g*, 7 min, 4 °C) to collect intact cells. The cells were resuspended in phosphate buffered saline (PBS) with 0.5 mM PMSF and 5 mM DTH (45 mL, 4 °C) and then centrifuged (800 *g*, 7 min, 4 °C) again to collect washed intact cells. The supernatants were decanted and disposed, and the cell pellets were resuspended in sucrose-HEPES-EDTA buffer with BSA, 2x protease inhibitors, and 5 mM DTH (0.28 M sucrose, 10 mM HEPES-KOH, pH 7.2, 0.2 mM EDTA, 1 mg/mL fatty acid free BSA, 2x Roche complete protease inhibitor mix, 0.5 mM freshly added PMSF, and 5 mM DTH) (final volume to 30 mL, 4 °C). Aliquots of whole-cell samples (300 uL) were centrifuged (1,000 *g*, 7 min, 4 °C) to isolate washed cell pellets as needed. The rest of the cell suspension was homogenized with a Dounce homogenizer (15 mL of cell suspension in a 15-mL glass Dounce homogenizer on cold pellets, 12 strokes with tight pestle, minimizing bubble formation). The whole-cell homogenate was transferred into a 50-mL conical tube. The combined cell homogenates were centrifuged at slow speed (800 *g*, 10 min, 4 °C) to pellet intact cells and nuclei. The slow-spin supernatant was recovered, transferred to a clean 50-mL conical tube on ice, and centrifuged again at slow speed (800 *g*, 10 min, 4 °C) to clear any remaining intact cells or nuclei. The slow-spin supernatant was recovered, transferred to a clean 50-mL conical tube on ice, and centrifuged at high speed (8,000 *g*, 10 min, 4 °C) to pellet mitochondria. The high-speed supernatant was decanted and disposed. The high-speed pellet of anoxic mitochondria was gently resuspended into an experimental buffer for assays as described below.

###### H_2(g)_ generation assays

Building on established protocols for [FeFe]-hydrogenase enzymology,^111^ the principle of this H_2(g)_ generation assay is to use reduced methyl viologen as an electron donor to allow HydA to catalyze the reaction: 2 H^+^ + 2 e^−^ → H_2_. Cells or isolated mitochondria were resuspended in reaction buffer (*e.g.*, potassium phosphate, 62.5 mM, pH 6.25) to a final reaction volume of 0.5 mL. Reactions were performed in a custom-machined air-tight acrylic chamber including space for the reaction fluid, a glass-coated stir bar, an O-ring sealed entry port for an H_2_ electrode, and a cap for addition of reagents. Reactions were started by the addition of reduced methyl viologen (10 mM methyl viologen and 30 mM DTH final concentrations). H_2_ produced was detected with an H_2_ sensor electrode in a 1/4″ steel tube (Unisense Cat# H2-ST-1/4, amperometric Clark-type electrode connected to an H_2_ UniAmp (amplifier for H_2_ electrode, Unisense Cat# H2 UNIAMP). Data was collected with SensorTrace Logger (Unisense Cat# STLOGGER, software for running H_2_ electrode).

###### H_2(g)_ consumption assays

Building on established protocols for [FeFe]-hydrogenase enzymology,^111^ the principle of this H_2(g)_ consumption assay is to use the electrons from HydA-catalyzed oxidation of H_2(g)_ to transform oxidized methyl viologen (colorless) into reduced methyl viologen (blue in color). The following procedure was performed in an anoxic chamber (2.5% H_2(g)_, 97.5% N_2(g)_), taking advantage of the 2.5% H_2(g)_ in the anaerobic chamber as the substrate for this H_2(g)_ consumption assay. Both adherent cells and non-adherent cells were harvested. Using the existing media in the flasks, the cells were mechanically detached from the tissue culture flask (hitting the back of the flask 5 times, hitting the side of the flask 5 times, and then rocking the flask back and forth 5 times) and transferred to 50-mL conical tubes. The harvested cells were centrifuged (1,000 *g*, 10 min, 4 °C) to collect intact cells. The harvested cell pellets were transferred back into the anaerobic chamber. The supernatant was decanted. The cell pellets were resuspended in 50 mM Tris HCl, pH 8, 50 mM KCl, 4% glycerol, 0.2% Tween 20 and mixed to generate a crude lysate, which was used as a direct input to the reaction. 1-mL reactions were conducted in plastic cuvettes. Methyl viologen (oxidized) was added to a final concentration of 10 mM to start the reaction inside of the anaerobic chamber with 2.5% H_2(g)_. The reactions were incubated (30 °C, 5 min) inside of a UV-Vis Spectrophotometer, DS-C Chrome, DeNovix (inside of Coy chamber), collecting spectra every 10 seconds. Pictures of the reaction cuvettes were also captured with a digital camera after the 5-minute reaction time was complete (shown in figure).

##### Chemical inhibitor assays

Chemical inhibitor (drug) assays with *Acanthamoeba* clinical isolates were performed using the CellTiter Glo assay essentially as described previously.^122^ Chemical stocks were prepared in DMSO (20 mM stocks). White, flat bottom, tissue culture treated 96-well plates were seeded with 4,000 *Acanthamoeba* trophozoites in a final volume of 100 uL of standard culture medium with antibiotics (Pen-Strep) (20 g/L proteose peptone, 1 g/L yeast extract, 4 mM MgSO_4_, 0.4 mM CaCl_2_, 3.4 mM sodium citrate, 50 uM Fe(NH_4_)_2_(SO_4_)_2_, 2.5 mM Na_2_HPO_4_, 2.5 mM KH_2_PO_4_, pH 6.5, 100 mM glucose, Pen-Strep). Chemical inhibitors were included at the indicated concentrations (all wells included the DMSO vehicle to a final concentration of 0.25%, v/v). *Acanthamoeba* lines were cultured with inhibitors under normoxia (21% O_2_, ambient air) at 30 °C in a dry incubator for 36 hours, after which the samples were processed per standard protocol for CellTiter Glo assay (Promega), including cell lysis and luminescence plate reading. Luminescence reads were processed as follows: (i) Calculated average signal in untreated (control) wells, (ii) Calculated relative signal for each (relative to the control average), (iii) analyzed and visualized data with GraphPad Prism software (Prism for macOS, Version 11.0.0 (93), Feb 10, 2026), using the standard approach for IC_50_ analysis using out of the box package without any constraints, nonlinear regression, and variable slope (four parameters).

### QUANTIFICATION AND STATISTICAL ANALYSIS

Statistical analyses were performed in Excel and R. Details of the benchmark analysis of published and newly generated mitochondrial proteomics datasets are provided in the Methods and include how benchmark datasets were created, corrected for size, and used to determine the sensitivity and precision of the MitoViaPCP list and previously published lists. Two-sided Student’s t-tests (shown in volcano plots and barplots in the figures) were performed using the function t.test in Excel on input data that met assumptions of normal distribution. Adjusted p-values were computed via Benjamini-Hochberg.

## Supplementary Material

1

2 **Data S1: MitoCarta Tree of Life (MitoTOL) consortium membership roster and co-submitted MitoTOL manuscripts,** related to STAR Methods.

3 **Table S1: Mass spectrometry proteomics data, related to** Figure 1. DIA MS intensity and iBAQ values for Protein Groups (**Tab A**) and peptides (**Tab B**) corresponding to the mitochondrial serial purification and MitoIP experiment (8 conditions x 3 biological replicates = 24 samples).

4 **Table S2: MitoViaPCP and AcMitoCarta, related to** Figure 1. Gene-based summary of mitochondrial Protein Correlation Profiling (PCP) results for all genes (**Tab A**), the MitoViaPCP inventory (**Tab B**), and the AcMitoCarta (**Tab C**). **Tab D** includes related sensitivity and precision values used to generate the MitoViaPCP proteomics receiver operating characteristic (ROC) curves shown in Figure 1.

5 **Table S3: Orthologous groups and MitoPathways, related to** Figure 2. Orthologous group (OG) assignment for *Acanthamoeba castellanii*, human, yeast, and *Dictyostelium*, including human MitoPathways annotations (**Tab A**), and human MitoPathway centric analysis (**Tab B** and **C**).

6 **Table S4: Mitochondrial complexome BN-PAGE-MS data, related to** Figure 3. Protein group-based MS iBAQ values and intensity values for BN-PAGE mitochondrial complexome analysis in Digitonin (60 slices) (**Tab A**) and DDM (60 slices) (**Tab B**) (120 total samples). **Tab C** shows relative protein abundance heatmaps for Digitonin and DDM (note that this shows a gene-based analysis as described in Methods, where protein groups are expanded such that protein group intensities are assigned to all proteins in the protein group). Tab C also shows correlations between markers of select complexes and the profile of each protein. **Tab D** shows gel slice by MW data. **Tab E** shows apparent and predicted MWs for key complexes.

7 **Table S5: IP-MS data, related to** Figure 3. LFQ DIA MS Intensity for Protein Groups (**Tabs A** and **C**) and peptides (**Tabs B** and **D**) corresponding to immunoprecipitation (IP) pulldown of selected tagged constructs. Note that this includes two sets of IPs, one set related to OXPHOS targets (Tabs A and B) and one set related to HydA (Tabs C and D). **Tab E** provides a combined gene-based summary.

8 **Table S6: Perturbation profiling proteomics data, related to** Figure 5. DIA-MS Intensity and iBAQ values for protein groups and peptides corresponding to O_2_ perturbation experiment (10 conditions x 3 replicates = 30 samples) (**Tabs A and B**) and the carbon (carbohydrate) and iron nutrient perturbation experiment (6 conditions x 3 replicates = 18 samples) (**Tabs C and D**), plus an integrated table with a gene-based summary (**Tab E**).

9

10 **Table S7: Perturbation profiling transcriptomics data, related to** Figure 5. RNA-seq data for both the O_2_ perturbation experiment (10 conditions x 3 replicates = 30 samples) and the carbon and iron nutrient perturbation experiment (6 conditions x 3 replicates = 18 samples) (total of 48 samples).

## Figures and Tables

**Figure 1.**
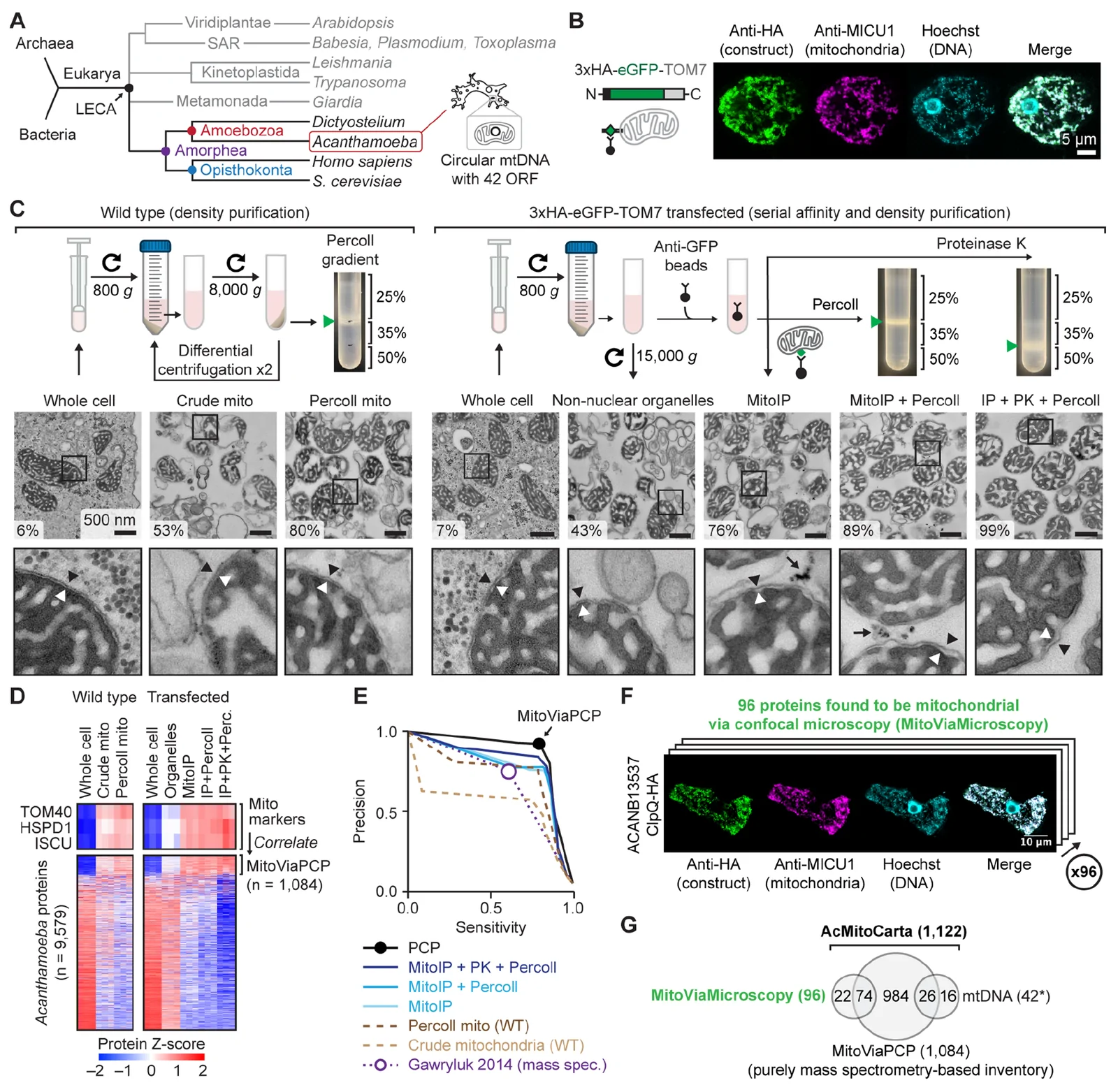
An experimentally defined *Acanthamoeba* MitoCarta via mass spectrometry proteomics and microscopy (A) Phylogenetic tree showing relationship of *Acanthamoeba* to other domains, clades, and organisms. SAR, stramenopiles, alveolates, and rhizarians. (B) Confocal immunofluorescence microscopy of *Acanthamoeba* transfected with 3xHA-eGFP-TOM7. DNA, mtDNA (small punctate structures in cytoplasm) and nuclear DNA. (C) Workflow for isolation of mitochondria and TEM of fractions (scale bar 500 nm for each). The inset percentage indicates the area of mitochondria relative to that of the total cellular material in representative TEM images. The inset box is expanded in the high-power image below. Black and white triangles, mito outer and inner membranes; arrow, magnetic beads. (D) Heatmap of relative abundances (Z-scores) of proteins quantified via DIA MS proteomics, sorted by correlation with the average profile of three mitochondrial marker proteins (top) using the transfected sample subset (n = 3 biological replicates). (E) Sensitivity-precision curves for fractions of increasing mitochondrial purity (log_2_(fraction/whole cell) of single replicates compared to protein correlation profiling (PCP) and the 2014 Gawryluk et al MS dataset.^33^ (F) Confocal microscopy of *Acanthamoeba* transfected with a C-terminal HA-tagged construct of the indicated protein, an example of 96 total proteins found to be mitochondrial via microscopy (see Figure S2). (G) Integration of MitoViaPCP with MitoViaMicroscopy and mtDNA to yield the AcMitoCarta. *Note: There are 42 mtDNA ORFs, but the COX1-2 fusion might be processed into two proteins.^33–35^. See also Figures S1 and S2.

**Figure 2.**
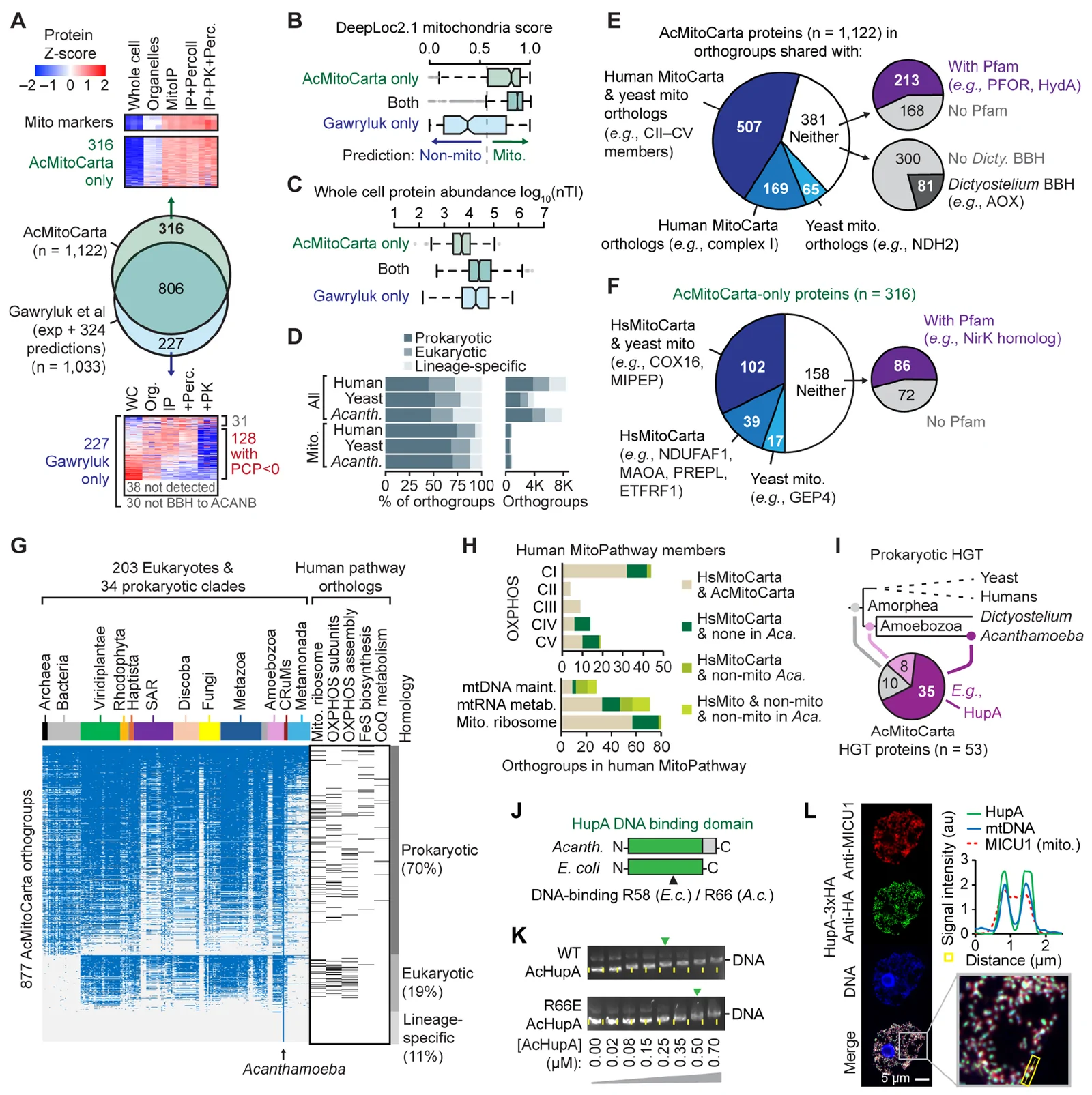
Unique features and mosaic ancestry of the AcMitoCarta (A) Venn diagram of AcMitoCarta and the mitoproteome of Gawryluk et al.^33^ Heat maps show Z-scores for our PCP proteomics. (B) Box plots of DeepLoc2.1 mito scores for the sets of proteins depicted in panel A. Center lines, medians; box limits, 25th & 75th percentiles; whiskers, 1.5x interquartile range (IQR); dots, outliers. n = 316, 806, 197. (C) For the protein sets depicted in panel A, box plots of whole cell protein abundance (log_10_ normalized MS intensity in PCP proteomics). Center lines, medians; box limits, 25th & 75th percentiles; whiskers, 1.5x IQR; dots, outliers. n = 301, 792, 153 sample points. (D) Comparison of mitochondrial protein homology relationships to those of all proteins from humans, yeast (*S. cerevisiae*), and *Acanthamoeba* (based on orthogroup membership; lineage indicates Opisthokonta or Amoebozoa). (E) Pie charts indicate shared AcMitoCarta orthogroup memberships with mitochondrial proteins of humans or yeast (*S. cerevisiae*). The fractions of unique mitochondrial proteins without a Pfam or without a bidirectional best hit (BBH) in *Dictyostelium* are also shown. (F) Pie charts as above, but for only the 316 AcMitoCarta-specific proteins indicated in the Venn diagram in panel A. (G) Presence and absence matrix for AcMitoCarta proteins and their orthologs across 203 eukaryotes, 34 prokaryotic clades, and a small set of archaea (rows indicate orthogroups in AcMitoCarta, columns indicate species, and blue marks indicate shared orthogroup membership). On the right, examples of orthogroups mapping to human MitoPathways, with tick marks indicating pathway members. (H) Human-centric MitoPathways analysis, with bar graphs indicating *Acanthamoeba* orthogroup mapping (or lack thereof) to mammalian MitoPathway members in the indicated pathways. (I) Fractions of AcMitoCarta proteins arising from prokaryotic HGT at the indicated evolutionary branch. (J) *Acanthamoeba* HupA protein domain schematic indicating a DNA binding domain shared with *E. coli* HupA (green). (K) Electromobility shift assay for DNA binding of WT & R66E mutant recombinant purified AcHupA. Yellow marks show baseline migration of DNA, which shifts when bound to HupA. The green triangle marks the lane in which a full shift above the baseline is observed. (L) Confocal microscopy of HupA-3xHA in *Acanthamoeba* showing co-localization with mtDNA. The signal intensity across one mitochondrion is quantified in the indicated box (yellow) as an example. See also Figure S3.

**Figure 3.**
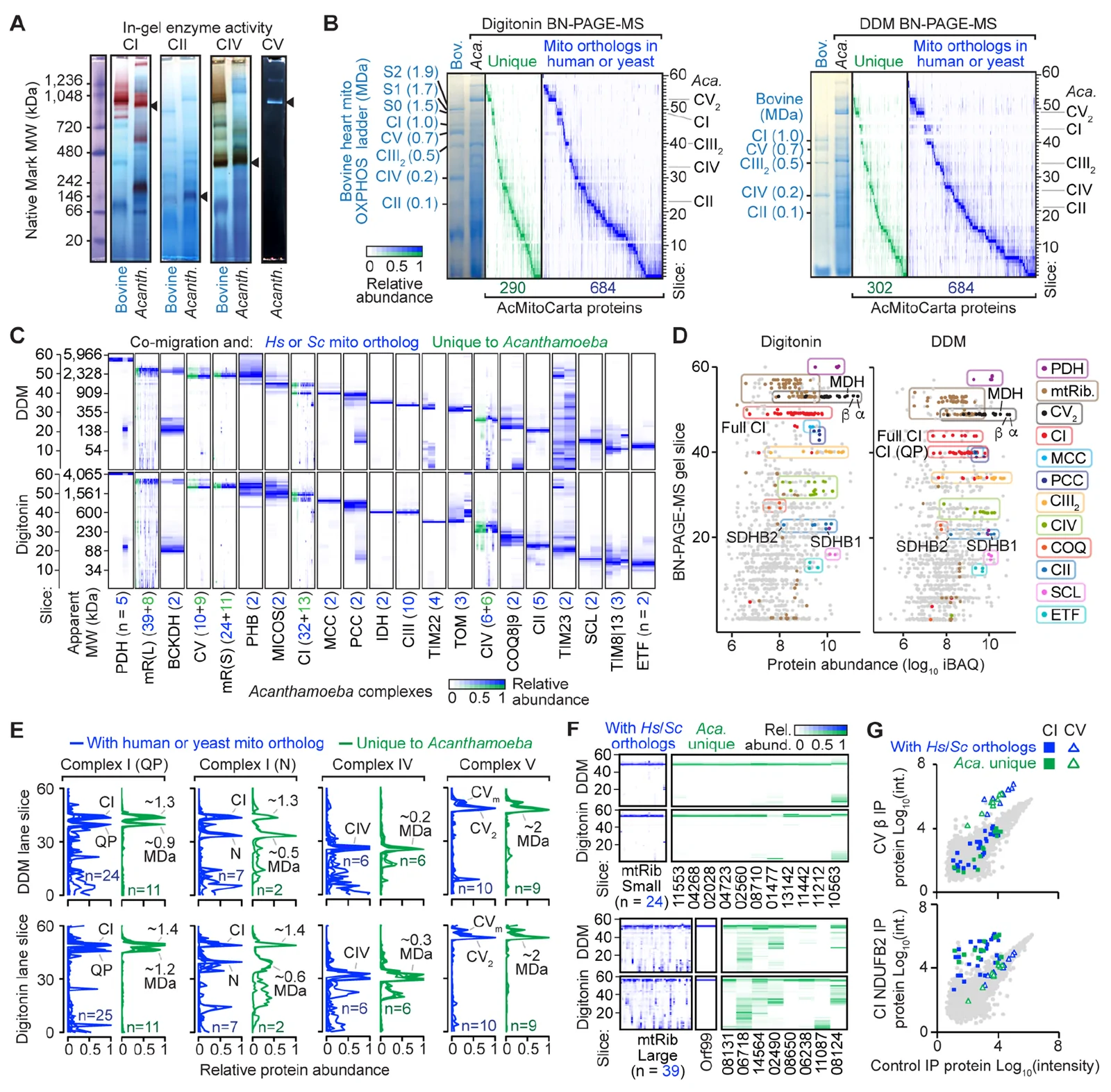
*Acanthamoeba* mitochondrial complexome via BN-PAGE-MS (A) Native PAGE in-gel activity assays for OXPHOS CI, II, IV, and V. Triangles indicate primary enzyme activity band. (B) BN-PAGE gels cut into 60 slices each for MS analysis. Heatmaps indicate relative abundances of mito proteins detected, with each protein normalized to maximum intensity across gel slices in each lane. Blue denotes proteins with orthologs in human or yeast mitochondria, while green denotes unique proteins in AcMitoCarta. S0-S2, supercomplexes of bovine OXPHOS. (C) Heatmaps indicating relative abundances of BN-PAGE-MS observed co-migrating protein members of each of the indicated complexes. Apparent MWs for slices are estimates based on migration of bovine OXPHOS complexes (see Figure S4A). Blue indicates conserved members of the homologous human complexes and green indicates unique members. (D) Apparent MWs and abundances of AcMitoCarta proteins identified in each BN-PAGE slice based on iBAQ MS proteomics. For visualization, only slices with relative abundance ≥0.9 are displayed. Complex members are defined in Table S4. (E) BN-PAGE-MS protein co-migration profiles for OXPHOS complexes I, IV, and V (ATP synthase). Apparent MWs (MDa) are indicated for major peaks. CV_2_, likely CV dimer; CV_m_, CV multimer (possible tetramer). (F) Heatmaps indicating relative abundances of observed co-migrating protein members of the large and small subunits of the mitochondrial ribosome across BN-PAGE-MS gel slices. MtDNA-encoded orf99 (homolog of MRPL19) is highlighted as an example. Numbers indicate ACANB gene ID numbers (*e.g.*, ACANB11553). (G) Scatter plots of protein abundance in IP-MS of CI (NDUFB2 subunit IP) and CV (ATP5F1B subunit IP). Control IP indicates the untransfected WT control. See also Figure S4.

**Figure 4.**
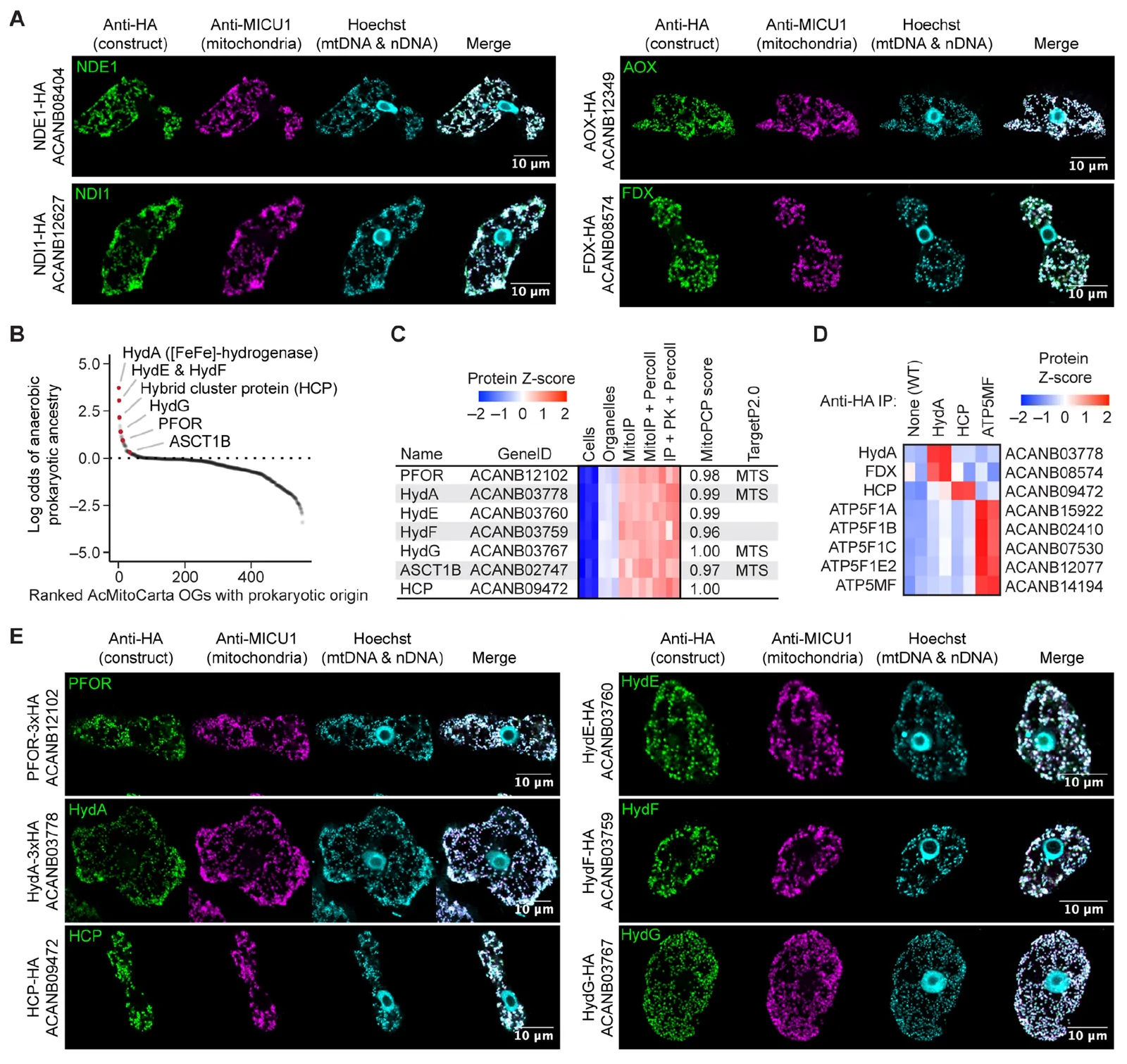
Aerobic and anaerobic bioenergetics pathways of *Acanthamoeba* mitochondria not seen in humans (A) Confocal microscopy of *Acanthamoeba* transfected with C-terminal HA-tagged constructs of the indicated proteins. (B) Rank plot of AcMitoCarta proteins based on log odds of anaerobic prokaryotic ancestry, highlighting key anaerobic enzymes. (C) Heatmap of relative protein abundance (Z-scores) across the PCP mitochondrial proteomics experiment (detailed in Figure 1, n = 3 biological replicates). MTS by TargetP2.0.^61^ (D) Heatmap (protein Z-scores) of selected proteins in IP-MS of HydA-3xHA, HCP-HA, a complex V subunit (ACANB14194), or control (untransfected cells) (n = 2 replicates). (E) Confocal microscopy of *Acanthamoeba* transfected with C-terminal HA-tagged constructs of the indicated proteins.

**Figure 5.**
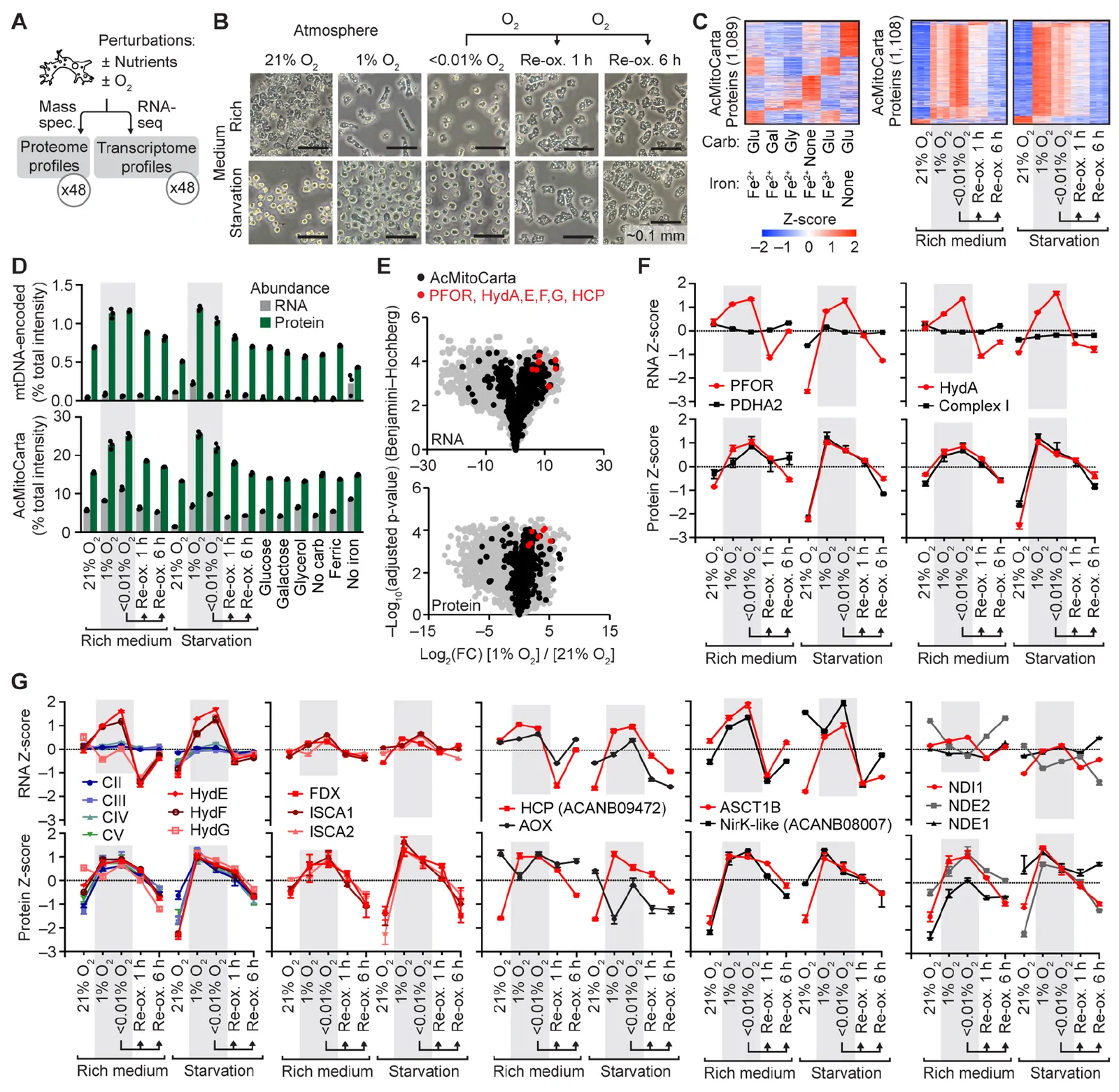
Dynamic remodeling of *Acanthamoeba* mitochondrial bioenergetics pathways in response to O_2_ fluctuations (A) Overview of perturbations and profiling approaches. (B) Phase contrast light microscopy of *Acanthamoeba* cultured under the indicated O_2_ atmosphere. (C) Heatmaps of relative AcMitoCarta protein abundances (Z-score) across the indicated perturbation conditions (n = 3 biological replicates, number of AcMitoCarta proteins detected by MS are indicated). (D) Bar graphs indicating the average relative total summed intensities (based on normalized total intensity for proteomics) of mitochondrial proteins (or encoding RNA, based on summed RNA-seq transcripts per million, TPM). Points indicate each of the three biological replicates. (E) Volcano plots of transcriptomic (top) or proteomic (bottom) profiling data for *Acanthamoeba* cultured under hypoxia (1% O_2_) compared to normoxia (21% O_2_) in starvation medium (n = 3 biological replicates) (two-sided Student’s t-test). (F) and (G) Protein and RNA Z-score trajectories for selected proteins and pathways across the O_2_ perturbation profiling conditions (n = 3 biological replicates indicated by points, lines connect the medians). CI–CV shows the means for nuclear DNA encoded complex members. Z-scores were calculated across all ten conditions shown. See also Figure S5.

**Figure 6.**
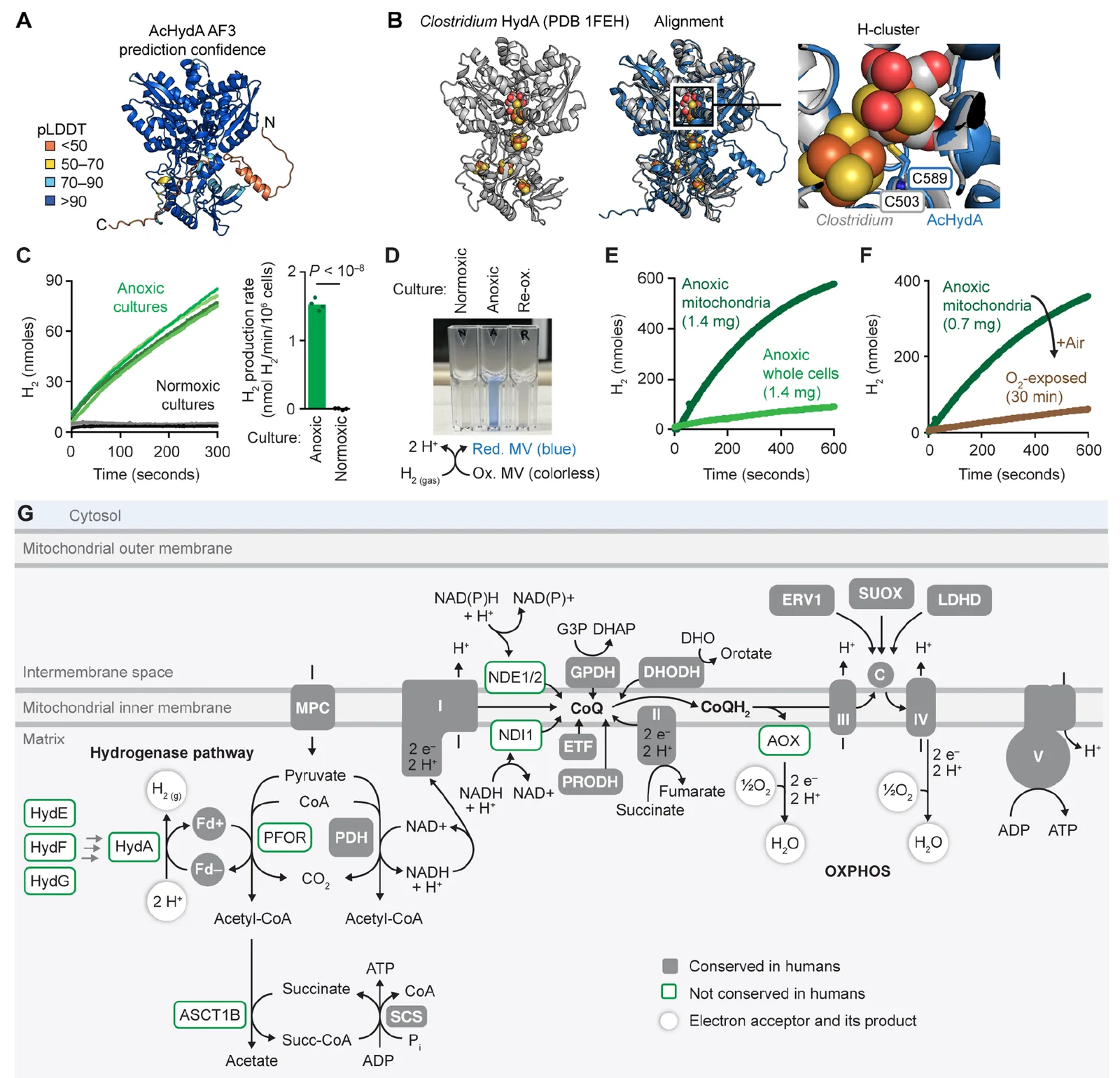
Hydrogenase activity in *Acanthamoeba* mitochondria (A) AlphaFold3^109^ model of *Acanthamoeba* HydA, colored by predicted local distance difference test (pLDDT). (B) AlphaFold3^109^ model of AcHydA (blue) aligned with the published crystal structure of *Clostridium pasteurianum* HydA (PDB 1FEH) (gray). Iron-sulfur clusters and the H-cluster are shown with spheres. Example of alignment of key H-cluster residues C589 (AcHydA) and C503 (*Clostridium* HydA). (C) H_2_ production capacity of *Acanthamoeba* cells after culturing under anoxia or normoxia, as measured using a Clark-type H_2_ specific electrode (n = 4 biological replicates, *P* value indicates two-sided Student’s t-test). (D) Colorimetric detection of hydrogenase activity in whole cells using methyl viologen (reduced form is blue). (E) H_2_ production capacity of equal masses of *Acanthamoeba* cells or mitochondria (each after culturing under anoxia). (F) H_2_ production capacity of *Acanthamoeba* mitochondria after a 30-minute exposure to air (21% O_2_), compared to mitochondria maintained under anoxia. (G) Model for *Acanthamoeba*’s highly branched mitochondrial ETC. Proteins or complexes seen in humans are shown in gray. Proteins not found in humans are shown in white boxes outlined with green. I–V, OXPHOS CI–V; C, cytochrome C; DHODH, dihydroorotate dehydrogenase; ERV1, flavin-linked sulfhydryl oxidase, essential for respiration and viability; Fd, ferredoxin; GPDH, glycerol-3-phosphate dehydrogenase; LDHD, D-lactate dehydrogenase; MPC, mitochondrial pyruvate carrier; PRODH, proline dehydrogenase; SUOX, sulfite oxidase. See also Figure S6.

**Figure 7.**
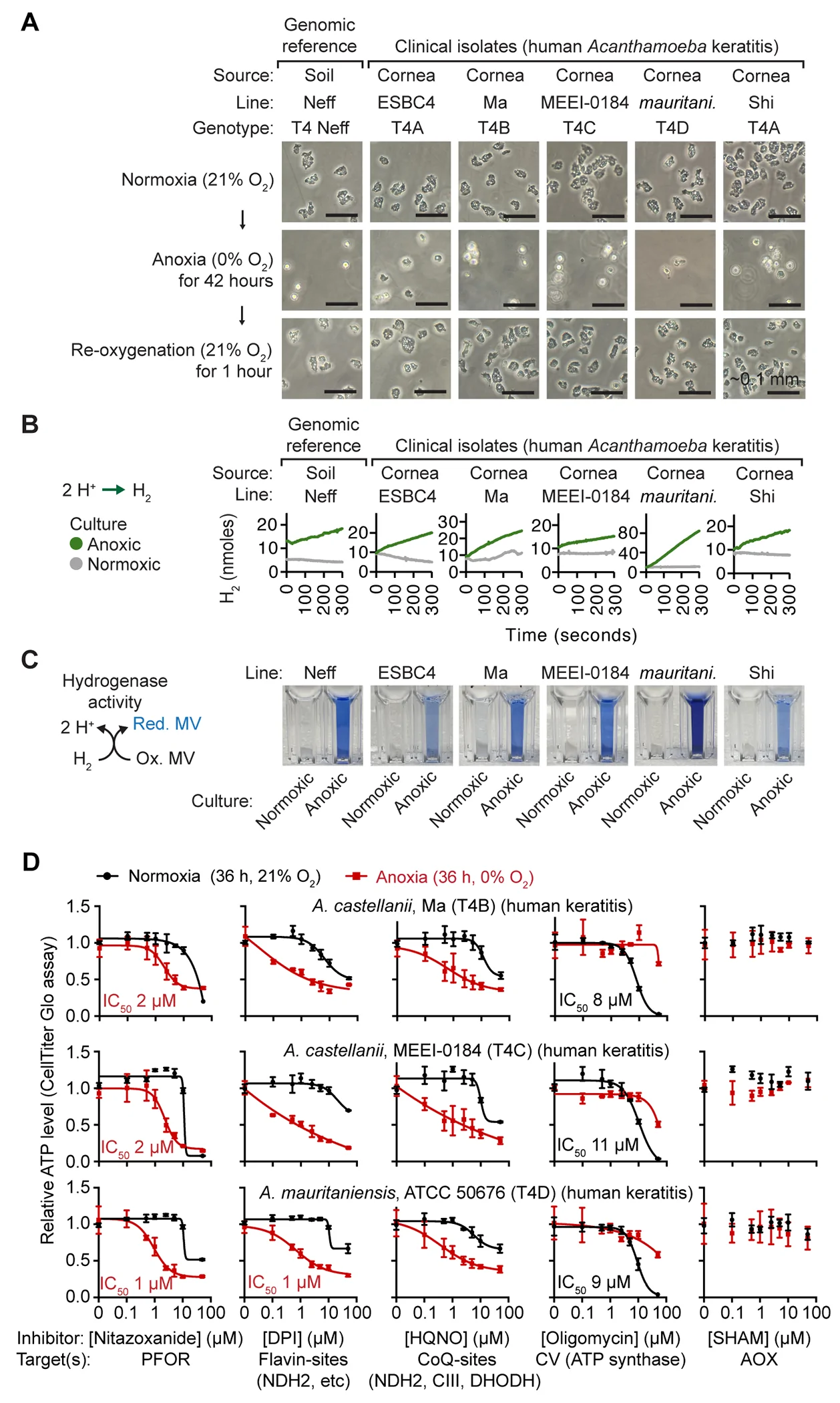
Chemical inhibition of *Acanthamoeba* lines under aerobic and anaerobic conditions (A)Phase contrast light microscopy of clinical *Acanthamoeba* lines or the Neff strain cultured under normoxia, anoxia (42 h), and then re-oxygenation (1 h). (B) H_2_ production capacity of clinical *Acanthamoeba* lines and the Neff strain after culturing under anoxia or normoxia, as measured using a Clark-type H_2_ specific electrode. (C) Colorimetric detection of hydrogenase activity in whole cells (clinical *Acanthamoeba* lines and the Neff strain) using methyl viologen (reduced form is blue). (D) Relative ATP levels (via luciferase-based CellTiter Glo assay) in clinical *Acanthamoeba* lines cultured under anoxia or normoxia with the indicated inhibitors (n = 3 biological replicates, error bars indicate standard deviation). IC_50_s were determined using nonlinear regression with variable slope (four parameters). See also Figure S7.

**Table:** KEY RESOURCES TABLE

REAGENT OR RESOURCE	SOURCE	IDENTIFIER
Antibodies		
Custom anti-AcMICU1 (*Acanthamoeba castellanii* MICU1) antibody, produced in Rabbit	This study	NA
HA-Tag (C29F4) Rabbit mAb	Cell Signaling	3724S
Pan-Actin (D18C11) Rabbit mAb	Cell Signaling	8456S
Anti-rabbit IgG, HRP-linked Antibody	Cell Signaling	7074S
HA Tag Monoclonal Antibody (16B12), Alexa Fluor^™^ 488	Thermo	A-21287
Goat anti-Rabbit IgG (H+L) Cross-Adsorbed Secondary Antibody, Alexa Fluor^™^ 647	Thermo	A21244
Bacterial strains		
NEB^®^ 5-alpha Competent *E. coli* (High Efficiency)	NEB	C2987H
NiCo21(DE3) Competent *E. coli*	NEB	C2529H
Chemicals, peptides, and recombinant proteins		
AcMICU1, recombinant protein, purified from *E. coli*	This study	
AcHupA (ACANB05819), recombinant protein, wild type, purified from *E. coli*	This study	
AcHupA (ACANB05819), recombinant protein, R66E mutant, purified from *E. coli*	This study	
6xHis-ULP1, recombinant protein, purified from *E. coli*	This study	
Streptavidin-HRP	Thermo	21126
Benzonase (Purity >99%)	Millipore	70664-3
Proteinase K	Sigma	P5568-1ML
Cytochrome c from equine heart	Sigma	C2506
N-oxo-2-heptyl-4-Hydroxyquinoline (HQNO)	Cayman Chemical	15159
Oligomycin	Sigma-Aldrich	495455
Nitazoxanide (NTZ)	Sigma-Aldrich	N0290
Salicylhydroxamic acid (SHAM)	Sigma-Aldrich	S607
Diphenyleneiodonium chloride (DPI)	MedChemExpress	HY-100965
Percoll	Cytiva	17089101
Aprotinin	Sigma	A6103-25MG
Leupeptin	Sigma, Roche	11017128001
β-Nicotinamide adenine dinucleotide, reduced disodium salt hydrate	Sigma	N8129
Nitrotetrazolium Blue chloride	Sigma	N6876
Iodonitrotetrazolium chloride	Sigma	I8377
Di-Sodium succinate	Sigma	8.18601
n-Dodecyl β-D-maltoside	Sigma	D5172
Phenazine methosulfate	Sigma	P9625
3,3′-Diaminobenzidine (DAB)	Sigma	D8001
EveryBlot Blocking Buffer	Bio-Rad	12010020
ProLong^™^ Glass Antifade Mountant with NucBlue^™^ Stain	Thermo	P36981
cOmplete^™^ Protease Inhibitor Cocktail	Sigma / Roche	11697498001
Methyl viologen dichloride hydrate	Sigma	856177-1G
Sodium dithionite (DTH) (Sodium hydrosulfite)	Sigma	157953-100G
Adenosine 5′-triphosphate disodium salt hydrate	Sigma	A26209
Lead nitrate	Sigma	228621
TRI Reagent Solution	Thermo	AM9738
Triethylammonium bicarbonate buffer (1.0 M, pH 8.5; TEAB in H_2_O)	Sigma	T7408-100ML
2XYT Broth, granulated	Research Products International	X15680-5000.0
Beta-meraptoethanol	Sigma	M6250
HiTrap TM SP HP cation exchange chromatography column	Cytivia	29051324
Ni-NTA Agarose	Qiagen	30230
Water	MilliporeSigma	WX0008-1
UltraPure SDS Solution, 10%	Invitrogen	15553-035
Triethylammonium bicarbonate buffer, 1 M	MilliporeSigma	18597-500ML
Magnesium chloride solution, BioUltra, Molecular Biology, ~1 M in H_2_O	Sigma-Aldrich	63069-100ML
Phenylmethanesulfonyl fluoride solution, 0.1 M in ethanol (T)	Sigma-Aldrich	93482-50ML-F
Pierce DTT (Dithiothreitol), No-Weigh Format	Thermo Scientific	A39255
Iodoacetamide	Sigma-Aldrich	A3221-10VL
S-Trap Mini Column	PROTIFI	C02-mini-40
S-Trap Micro Column	PROTIFI	C02-micro-40
Phosphoric Acid	LabChem	LC186401
Formic Acid	MilliporeSigma	5330020050
Acetonitrile	J.T.Baker	9829-03
Sep-Pak tC18 96-well Plate, 40 mg Sorbent per Well	Waters	186002320
Empore C18 Extraction Disks	CSD Analytical	76333-132
Methanol	Honeywell	34966-4X4L
Trifluoroacetic acid	Sigma-Aldrich	302031-10X1ML
1-Propanol	Sigma-Aldrich	279544-1L
Sequencing Grade Modified Trypsin	Promega	V5113
Lysyl EndopeptidaseR, Mass Spectrometry Grade (Lys-C)	FUJIFILM Wako Pure Chemical Corporation	129-02541
Evotips	Evosep	EV2011
Evosep Endurance Column (0.15 mm x 15 cm, 1.9μm)	Evosep	EV1106
Evosep Stainless Steel Emitter (ID 30μm)	Evosep	EV1086
0.50 mL Alphanumeric Micronic Vial Rack	Micronic	MPW42072LBC3
Matrix SepraSeal Cap Mats	Thermo Scientific	4466BLU
96-Well Microplate Flat Bottom Clear	VWR	82050-760
PepSep C18 25 cm x 75 μm, 1.5 μm	Bruker Daltonics	1893484
Aurora Elite 15×75 CSI C18 UHPLC column	IonOpticks	AUR4-15075C18-CSI
UNIPLATE microplates	Whatman	7701-5200
Critical commercial assays		
Pierce BCA Protein Assay Kit	Thermo Fischer Scientific	23225
CellTiter-Glo^®^ Luminescent Cell Viability Assay	Promega	G7571
Deposited data		
*Acanthamoeba* mitochondrial protein correlation profiling (MitoPCP) mass spectrometry proteomics dataset	This study	MSV000101796
*Acanthamoeba* Blue Native PAGE Mass Spectrometry (BN-PAGE-MS) proteomics dataset, mitochondrial solubilized in DDM	This study	MSV000101796
*Acanthamoeba* Blue Native PAGE Mass Spectrometry (BN-PAGE-MS) proteomics dataset, mitochondrial solubilized in digitonin	This study	MSV000101796
*Acanthamoeba* hypoxia whole cell perturbation mass spectrometry proteomics dataset	This study	MSV000101796
*Acanthamoeba* carbon and iron whole cell perturbation mass spectrometry proteomics dataset	This study	MSV000101796
*Acanthamoeba* OXPHOS complex (I, IV, and V) immunoprecipitation mass spectrometry (IP-MS) proteomics dataset	This study	MSV000101796
*Acanthamoeba* HydA immunoprecipitation mass spectrometry (IP-MS) proteomics dataset	This study	MSV000101796
*Acanthamoeba* hypoxia, carbon, and iron whole cell perturbation RNA-seq transcriptomics dataset	This study	GSE338845
*Acanthamoeba* MitoCarta Confocal Microscopy	This study	S-BIAD3379
Experimental models: Organisms/strains		
*Acanthamoeba castellanii* strain Neff, wild type genome reference strain, genotype T4 Neff	ATCC	30010
*Acanthamoeba mauritani*, Pussard and Pons, SAWE90/1, clinical keratitis, genotype T4D	ATCC	50676
*Acanthamoeba castellanii* strain Ma, clinical keratitis, genotype T4B	ATCC	50370
*Acanthamoeba castellanii* strain MEEI-0184, clinical keratitis, genotype T4C, originally a local clinical isolate from Massachusetts Eye and Ear Institute.	Noorjahan Panjwani	PMID: 16113295
*Acanthamoeba castellanii* strain ESBC4, clinical keratitis, genotype T4A, originally via Public Health Laboratory Service (PHLS) in the United Kingdom.	Noorjahan Panjwani	PMID: 16505041
*Acanthamoeba castellanii* strain “Shi”, clinical keratitis, genotype T4A, originally via Public Health Laboratory Service (PHLS) in the United Kingdom.	Noorjahan Panjwani	PMID: 16505041
Recombinant DNA		
GAPDH-EGFP-polyAtailRev circular plasmid vector for *Acanthamoeba* (backbone used for the plasmids below)	John Samuelson, Boston University	N/A
Plasmid: JASp002_3xHA-eGFP-AcTOM7 [ACANB09923]	This study	N/A
Plasmid: FDp048_AcHupA-3xHA [ACANB05819]	This study	N/A
Plasmid: FDp049_PFOR-3xHA [ACANB12102]	This study	N/A
Plasmid: FDp050_HydA-3xHA [ACANB03778]	This study	N/A
Plasmid: FDp051_AcMCU-3xHA [ACANB08247]	This study	N/A
Plasmid: FDp052_ACANB00781-3xHA	This study	N/A
Plasmid: FDp053_ACANB05578-3xHA	This study	N/A
Plasmid: FDp055_ACANB06593-3xHA	This study	N/A
Plasmid: FDp056_ACANB14194-3xHA	This study	N/A
Plasmid: FDp057_ACANB02343-3xHA	This study	N/A
Plasmid: FDp071_ACANB10343-HA	This study	N/A
Plasmid: FDp073_ACANB05718-HA	This study	N/A
Plasmid: FDp075_ACANB10528-HA	This study	N/A
Plasmid: FDp076_ACANB08131-HA	This study	N/A
Plasmid: FDp077_ACANB12896-HA	This study	N/A
Plasmid: FDp079_ACANB03779-HA	This study	N/A
Plasmid: FDp080_ACANB15245-HA	This study	N/A
Plasmid: FDp081_ACANB00148-HA	This study	N/A
Plasmid: FDp082_ACANB08348-HA	This study	N/A
Plasmid: FDp083_ACANB02609-HA	This study	N/A
Plasmid: FDp085_ACANB01257-HA	This study	N/A
Plasmid: FDp086_ACANB00213-HA	This study	N/A
Plasmid: FDp087_ACANB09930-HA	This study	N/A
Plasmid: FDp088_ACANB05979-HA	This study	N/A
Plasmid: FDp091_ACANB12651-HA	This study	N/A
Plasmid: FDp092_ACANB01367-HA	This study	N/A
Plasmid: FDp093_NDUFB2-HA [ACANB10013]	This study	N/A
Plasmid: FDp095_Cox20-HA [ACANB12771]	This study	N/A
Plasmid: FDp096_Cox10-HA [ACANB09223]	This study	N/A
Plasmid: FDp097_ACANB07515-HA	This study	N/A
Plasmid: FDp098_ACANB15172-HA	This study	N/A
Plasmid: FDp099_FDX-HA [ACANB08574]	This study	N/A
Plasmid: FDp100_ACANB09473-HA	This study	N/A
Plasmid: FDp101_ACANB10705-HA	This study	N/A
Plasmid: FDp102_ACANB11954-HA	This study	N/A
Plasmid: FDp103_ACANB04460-HA	This study	N/A
Plasmid: FDp106_ACANB04724-HA	This study	N/A
Plasmid: FDp107_ACANB14707-HA	This study	N/A
Plasmid: FDp108_ATP5F1B-6His-HA (ACANB02410)	This study	N/A
Plasmid: FDp109_ACANB03888-HA	This study	N/A
Plasmid: FDp110_ACANB06490-HA	This study	N/A
Plasmid: FDp111_ACANB11190-HA	This study	N/A
Plasmid: FDp112_OsmC-HA [ACANB09029]	This study	N/A
Plasmid: FDp113_ACANB00733-HA	This study	N/A
Plasmid: FDp114_ACANB02298-HA	This study	N/A
Plasmid: FDp118_ACANB04196-HA	This study	N/A
Plasmid: FDp121_Romo1-HA [ACANB03153]	This study	N/A
Plasmid: FDp122_Mpv17-HA [ACANB07266]	This study	N/A
Plasmid: FDp127_UQCRH-HA [ACABI09199_t1] [ACANB09203]	This study	N/A
Plasmid: FDp128_ETFRF1-HA [ACANB05786]	This study	N/A
Plasmid: FDp136_HydF-HA [ACANB03759]	This study	N/A
Plasmid: FDp138_HCP-HA [ACANB09472]	This study	N/A
Plasmid: FDp142_ACANB00049-HA	This study	N/A
Plasmid: FDp143_ACANB03144-HA	This study	N/A
Plasmid: FDp144_ACANB12223-HA	This study	N/A
Plasmid: FDp148_ACANB14825-HA	This study	N/A
Plasmid: FDp152_ ACANB02332-HA	This study	N/A
Plasmid: FDp159_TIMM22-HA (ACANB11083)	This study	N/A
Plasmid: FDp160_Coq9-HA (ACANB03812)	This study	N/A
Plasmid: FDp161_ACANB03485-HA	This study	N/A
Plasmid: FDp164_ACANB10746-HA	This study	N/A
Plasmid: FDp167_ACANB06558-HA	This study	N/A
Plasmid: FDp168_ACANB04387-HA	This study	N/A
Plasmid: FDp169_ATP10-HA (ACANB02405)	This study	N/A
Plasmid: FDp170_ACANB05570-HA	This study	N/A
Plasmid: FDp171_FumeraseC-HA [ACANB05801]	This study	N/A
Plasmid: FDp172_AOX-HA [ACANB12349]	This study	N/A
Plasmid: FDp175_HydE-HA [ACANB03760]	This study	N/A
Plasmid: FDp176_HydG-HA [ACANB03767]	This study	N/A
Plasmid: FDp183_ACANB04425-HA-StrepTagII	This study	N/A
Plasmid: TMIp113_SQOR-HA (ACANB03641)	This study	N/A
Plasmid: TMIp114_NDE1-HA (ACANB08404)	This study	N/A
Plasmid: TMIp115_NDI1-HA (ACANB12627)	This study	N/A
Plasmid: TMIp117_ACANB14329-HA	This study	N/A
Plasmid: TMIp134_ACANB03328-HA	This study	N/A
Plasmid: TMIp135_ACANB05509-HA	This study	N/A
Plasmid: TMIp136_ACANB12766-HA	This study	N/A
Plasmid: TMIp137_ACANB06015-HA	This study	N/A
Plasmid: TMIp138_ACANB05560-HA	This study	N/A
Plasmid: TMIp151_ClpQ-HA (ACANB13537)	This study	N/A
Plasmid: TMIp152_CLS-HA (ACANB11675)	This study	N/A
Plasmid: TMIp154_NirK-HA (ACANB08007)	This study	N/A
Plasmid: TMIp156_GPD2-HA (ACANB10817)	This study	N/A
Plasmid: TMIp157_DHODH-HA (ACANB01358)	This study	N/A
Plasmid: TMIp158_GFER-HA (ACANB10606)	This study	N/A
Plasmid: TMIp160_LDHD-HA (ACANB06998)	This study	N/A
Plasmid: TMIp162_ETFA-HA (ACANB10428)	This study	N/A
Plasmid: TMIp163_MRPL34-HA (ACANB09489)	This study	N/A
Plasmid: TMIp165_MRPL21-HA (ACANB07091)	This study	N/A
Plasmid: TMIp167_MPC1-HA (ACANB08877)	This study	N/A
Plasmid: TMIp168_C5orf63-HA (ACANB02697)	This study	N/A
Plasmid: TMIp169_ACANB11884-HA	This study	N/A
Plasmid: TMIp170_MTX1-HA (ACANB07918)	This study	N/A
Plasmid: TMIp171_ALKBH7-HA (ACANB04895)	This study	N/A
Plasmid: TMIp172_FAHD1-HA (ACANB09218)	This study	N/A
Plasmid: TMIp174_LYRM9-HA (ACANB14642)	This study	N/A
Plasmid: TMIp176_CPOX-HA (ACANB01598)	This study	N/A
Plasmid: TMIp195_3xHA-eGFP-MIRO2 (ACANB10948)	This study	N/A
Plasmid: AcMICU1_pET30a+	This study	N/A
Plasmid: His-SUMO-AcHupA (ACANB05819)	This study	N/A
Plasmid: His-SUMO-AcHupA (ACANB05819) R66E mutant	This study	N/A
Plasmid: pET33b-6xHis-ULP1	Bil Clemons, Caltech	N/A
Software and algorithms		
Excel for Mac	Microsoft	Version 16.102.3
FIJI (Image J)	National Institutes of Health	
SensorTrace Logger (for H_2_ sensor electrode)	Unisense	STLOGGER
Prism 11 for Mac		Version 11.0.0 (93)
PyMOL	Schrödinger	3.1.5.1
Other		
μMACS^™^ GFP Isolation Kit	Miltenyi	130-091-122
Pre-Separation Filters (30 μm)	Miltenyi	130-041-407
LS Columns	Miltenyi	130-042-401
QuadroMACS^™^, magnetic separator and multi stand	Miltenyi	130-091-051
H_2_ sensor electrode in a 1/4” steel tube	Unisense	H2-ST-1/4
H_2_ UniAmp amplifier for H_2_ electrode	Unisense	H2 UNIAMP
Vinyl Anaerobic Chamber	Coy Lab Products	Type C
Lipofectamine 3000 Kit	Thermo	L3000001
Protein A kit	Thermo	89960
Mini-Beadbeater-16	BioSpec	607
Glass beads (0.5 mm diameter)	BioSpec	11079105
NativePAGE Sample Prep Kit	Thermo	BN2008
NativePAGE Running Buffer Kit	Thermo	BN2007
NativePAGE Novex 3% to 12% Bis-Tris protein gels	Thermo	BN2011BX10
GenCatch TM Plasmid DNA Mini-Prep Kit	Epoch life sciences	2160250
Bovine Heart Mitochondria	Abcam	ab110338
Direct-zol RNA Miniprep	Zymo Research	R2050
Q5 Site-Directed Mutagenesis Kit	New England Biolabs	E0554S

## Data Availability

Code for orthogroup and HGT pipelines is available on GitHub: https://github.com/michaelzhuchen. The original mass spectra and protein sequence database have been deposited in the public proteomics repository MassIVE (http://massive.ucsd.edu) (MSV000101796). Confocal microscopy data have been deposited at the BioImage Archive (SBIAD3379). Bulk RNA-seq data have been deposited at the NCBI GEO data repository (GSE338845).

## Highlights

- Experimentally defined *Acanthamoeba* mitoproteome with 1,122 proteins
- 20 *Acanthamoeba* mitochondrial complexes mapped by BN-PAGE-MS
- Dynamic O_2_-dependent remodeling of bioenergetics including an anaerobic hydrogenase
- *Acanthamoeba* derived from human eye infections can produce hydrogen gas in anoxia
