# Supplementary material for: Mitochondrial proteome of Acanthamoeba delineates aerobic and anaerobic pathways dynamically regulated by oxygen
Source: Cell. Author manuscript; Available in PMC 2026 Oct 2. (PMC13632651; doi:10.1016/j.cell.2026.08.056)
Supplement: Supplementary file 1 [file NIHMS2208820-supplement-1.pdf]

Supplemental Figures

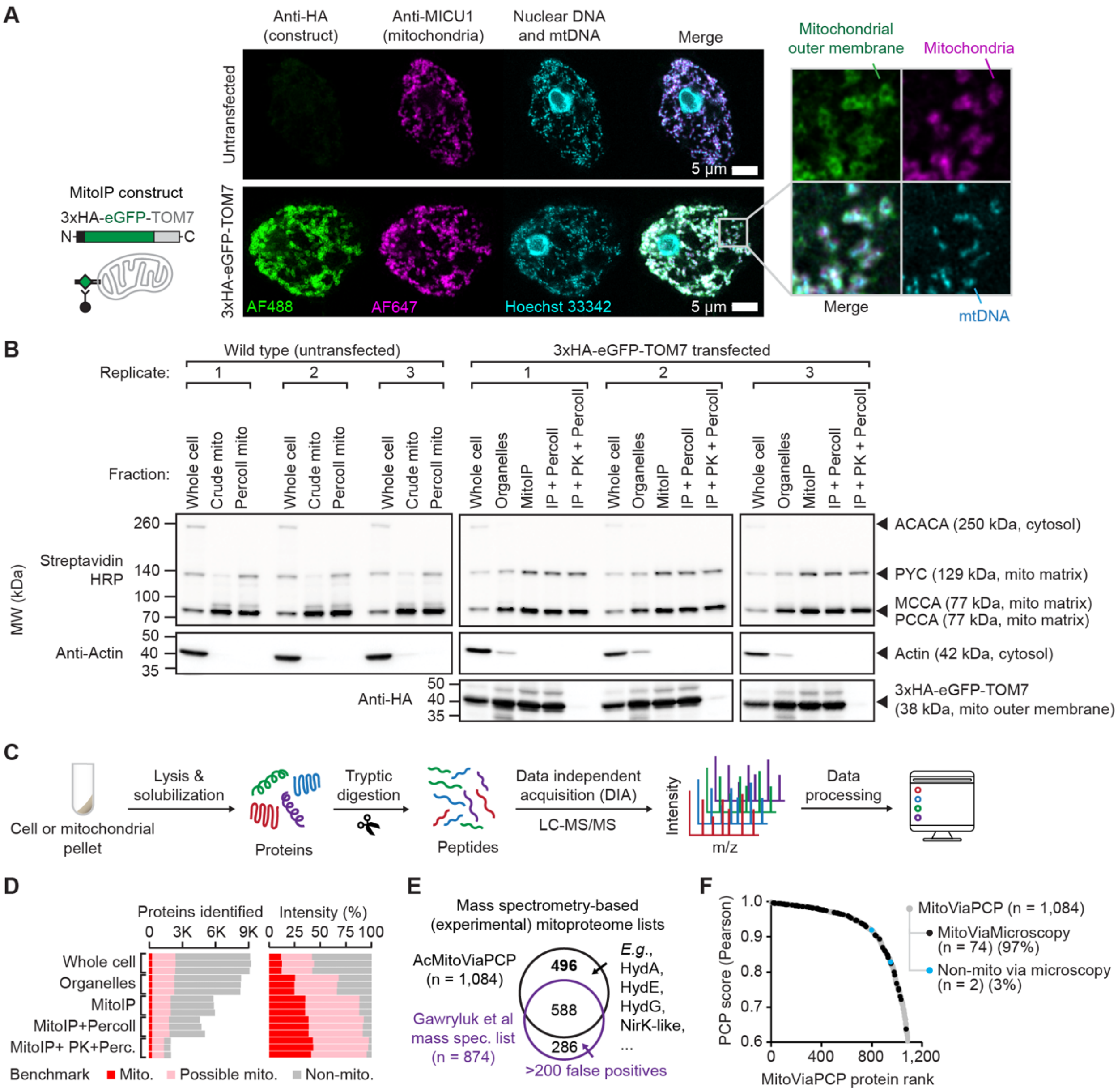

Figure S1. Mass spectrometry-based mito via protein correlation profiling (MitoViaPCP) inventory, related to Figure 1

(A) Confocal microscopy of *Acanthamoeba* transfected with 3xHA-eGFP-TOM7 (and an untransfected wild type control). GFP, green fluorescence protein; AF488, AlexaFluor488; AF647, AlexaFluor647. Note that the same 3xHA-eGFP-TOM7 transfected images are also shown in **Figure 1B** (without the untransfected control or the 'zoom in' shown here).

(B) Immunoblots of fractions (detailed in **Figure 1C**) collected during isolation of mitochondria (biological triplicate, 1–3). The streptavidin-HRP blot shows endogenously biotinylated proteins: ACACA, acetyl-CoA carboxylase alpha, cytosolic; PYC, pyruvate carboxylase, mitochondrial; MCCA, methylcrotonyl-CoA carboxylase alpha, mitochondrial; PCCA, propionyl-CoA carboxylase alpha, mitochondrial.

(C) Overview of MS-based mitochondrial proteomics workflow. m/z, mass to charge ratio.

(D) Number of protein groups identified and quantified by MS proteomics in fractions of increasing mitochondrial purity. The fractions of total intensity are also shown. Sets of proteins were benchmarked as mitochondrial, possible mitochondrial, or non-mitochondrial (see Methods for details of benchmarking).

(E) Venn Diagram of AcMitoViaPCP list compared to the Gawryluk et al (2014)[1] mass spectrometry-based list.

(F) Summary of microscopy results for 76 MitoViaPCP proteins tested, ranked by MitoViaPCP score.

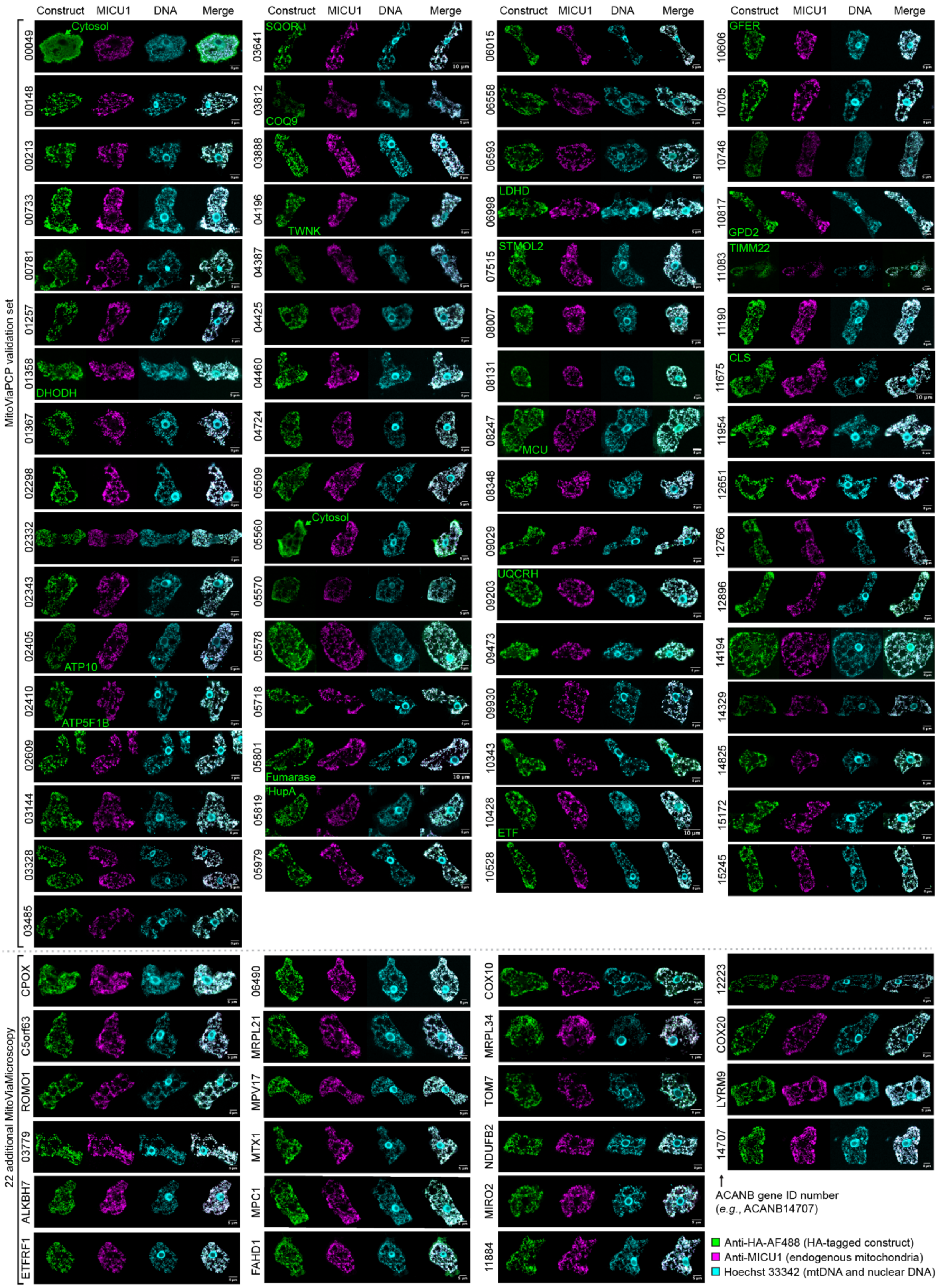

Figure S2. Confocal immunofluorescence microscopy, related to Figure 1 (legend on next page)

Figure S2. Confocal immunofluorescence microscopy, related to Figure 1 (figure on prior page)

Confocal fluorescence microscopy of *Acanthamoeba* transfected with HA- or 3xHA- tagged constructs of the indicated proteins (ACANB gene ID numbers are shown to the left of each image set; *e.g.*, 14707 refers to ACANB14707). Most of the constructs shown are C-terminal -HA or -3xHA constructs, except for N-terminal tags on TOM7 (3xHA-eGFP-TOM7) and MIRO2 (RHOT2, 3xHA-eGFP-MIRO2). Anti-HA, construct; anti-MICU1, mitochondria; DNA, mtDNA (small punctate structures in cytoplasm) and nuclear DNA. This figure shows 65 of the 76 tested MitoViaPCP proteins. One additional image is shown in **Figure 1**, and 10 additional images are shown in **Figure 4**. Twenty-two additional mitochondrial proteins via microscopy (MitoViaMicroscopy-only) are also shown.

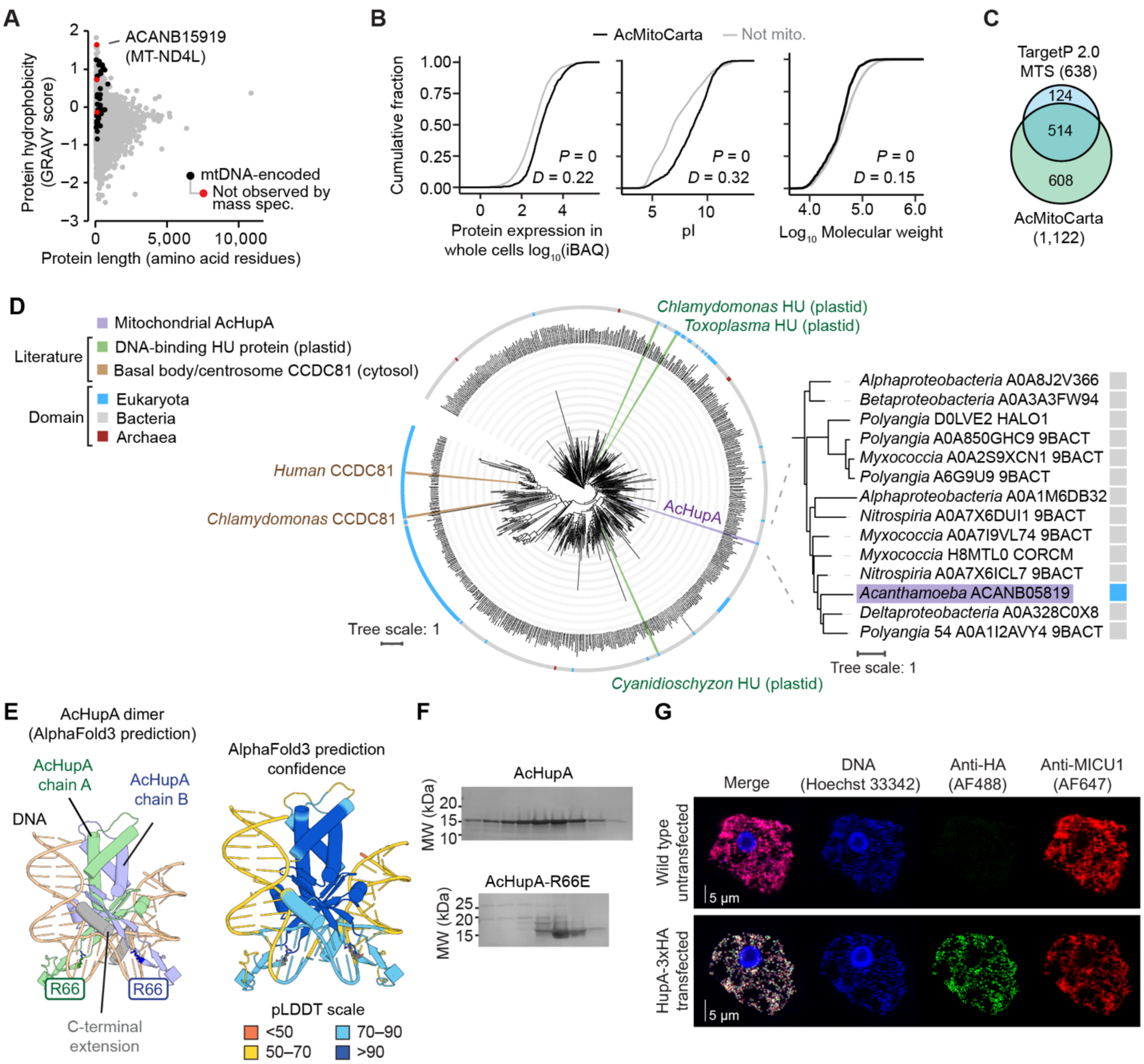

Figure S3. AcMitoCarta features and an example of an HGT-derived mitochondrial protein, HupA, related to Figure 2

(A) Scatter plot of protein hydrophobicity scores (GRAVY scores) and protein length, highlighting mtDNA ORFs. Red points indicate mtDNA-encoded proteins not detected by MS proteomics.

(B) Cumulative distribution function plots for AcMitoCarta proteins (black) compared to non-mitochondrial proteins (gray). Significance assessed by Kolmogorov-Smirnov test. Log_{10} iBAQ quantitation MS proteomics (based on a single replicate of the WT cells; n = 8,157 non-mito, n = 1,082 mito proteins). pI, predicted isoelectric point of the protein (BioPerl pICalculator; n = 14,822 non-mito, n = 1,122 mito). Log_{10}(MW), n = 14,822 non-mito, n = 1,122 mito proteins.

(C) Venn diagram indicating the overlap between AcMitoCarta proteins (n = 1,122) and Target 2.0 MTS predictions (n = 638).

(D) Phylogeny of HupA reveals multiple independent HGT to eukaryotes. Species domain annotations are shown for eukaryotes (blue), bacteria (gray), and archaea (red). Literature-based annotations are shown for *Cyanidioschyzon merolae* chloroplast HupA[2], *Chlamydomonas* plastid HupA[3], and *Toxoplasma* plastid HupA[4] (each in green). Cytosolic basal body/centrosome CCDC81 homologs[5] from humans[6] and *Chlamydomonas*[7] are also indicated (brown).

(E) AlphaFold3[8] model of AcHupA dimer bound to double-stranded DNA (dsDNA), on the right colored by predicted local distance difference test (pLDDT) from low to high confidence.

(F) Coomassie-stained sodium dodecyl sulfate-polyacrylamide gel electrophoresis (SDS-PAGE) gels of purified AcHupA (wild type) and AcHupA-R66E (mutant) after ion exchange purification.

(G) Confocal fluorescence microscopy of AcHupA-3xHA in *Acanthamoeba* (same image as in **Figure 2**), shown here next to untransfected (wild type) control for direct side-by-side comparison.

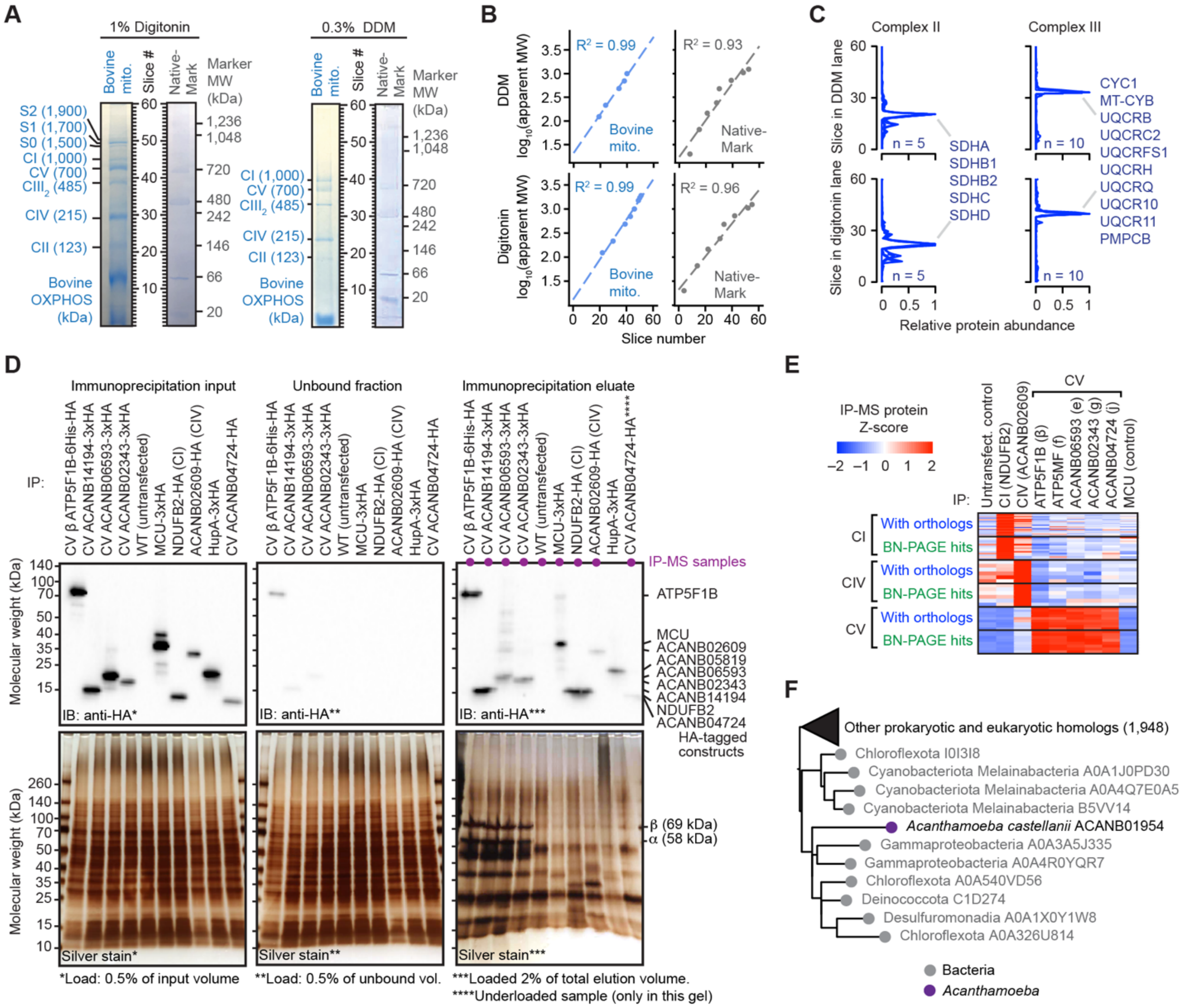

Figure S4. BN-PAGE-MS complexome profiling data analysis and IP-MS validation, related to Figure 3

(A) Images of BN-PAGE gel lanes with MW markers that were run in parallel with the *Acanthamoeba* mito BN-PAGE-MS lanes shown in **Figure 3B** (note that the bovine lanes shown here are identical to the bovine lanes shown in the main figure). The lanes shown here have either bovine mitochondria OXPHOS complex bands (with known MWs) or a pre-stained ladder of soluble proteins (NativeMark). Slice numbers for BN-PAGE-MS of an adjacent *Acanthamoeba* mito lane (see main figure) are indicated. CI, complex I; CII, complex II, CIII, complex III; CIV, complex IV; CV, complex V; S0-S2, OXPHOS supercomplexes in the bovine mitochondria sample.

(B) Analysis of MW by slice across the BN-PAGE gels using either the bovine mito bands or a pre-stained ladder (NativeMark).

(C) BN-PAGE-MS protein co-migration profiles for OXPHOS complexes II and III.

(D) Anti-HA immunoblots and silver stain protein gels of immunoprecipitation fractions. WT, wild type (untransfected); IP-MS, immunoprecipitation-mass spectrometry (samples corresponding to IP-MS datasets reported here are indicated with violet dots on the right gels). The far-right lane (IP eluate for ACANB04724) was underloaded due to SDS-PAGE sample loss, but the related IP-MS sample was not affected. The IP eluate samples for the CV constructs show a distinct pattern of bands (a "barcode") likely corresponding to CV subunits. AcMCU and AcHupA serve as non-OXPHOS controls in this IP.

(E) Heatmaps of protein abundance in IP-MS of OXPHOS complexes I, IV, and V. Groups of proteins with orthologs in human or yeast mitochondria are indicated ("with orthologs" in blue). BN-PAGE-MS hits (unique to *Acanthamoeba*, without orthologs in their human or yeast complexes) are also indicated. MCU, homolog of mitochondrial calcium uniporter[9].

(F) Phylogeny of the orthogroup containing ACANB01954 (*Acanthamoeba* member shown in purple), with distant homologs collapsed to focus on its bacterial sister and cousin homologs (gray).

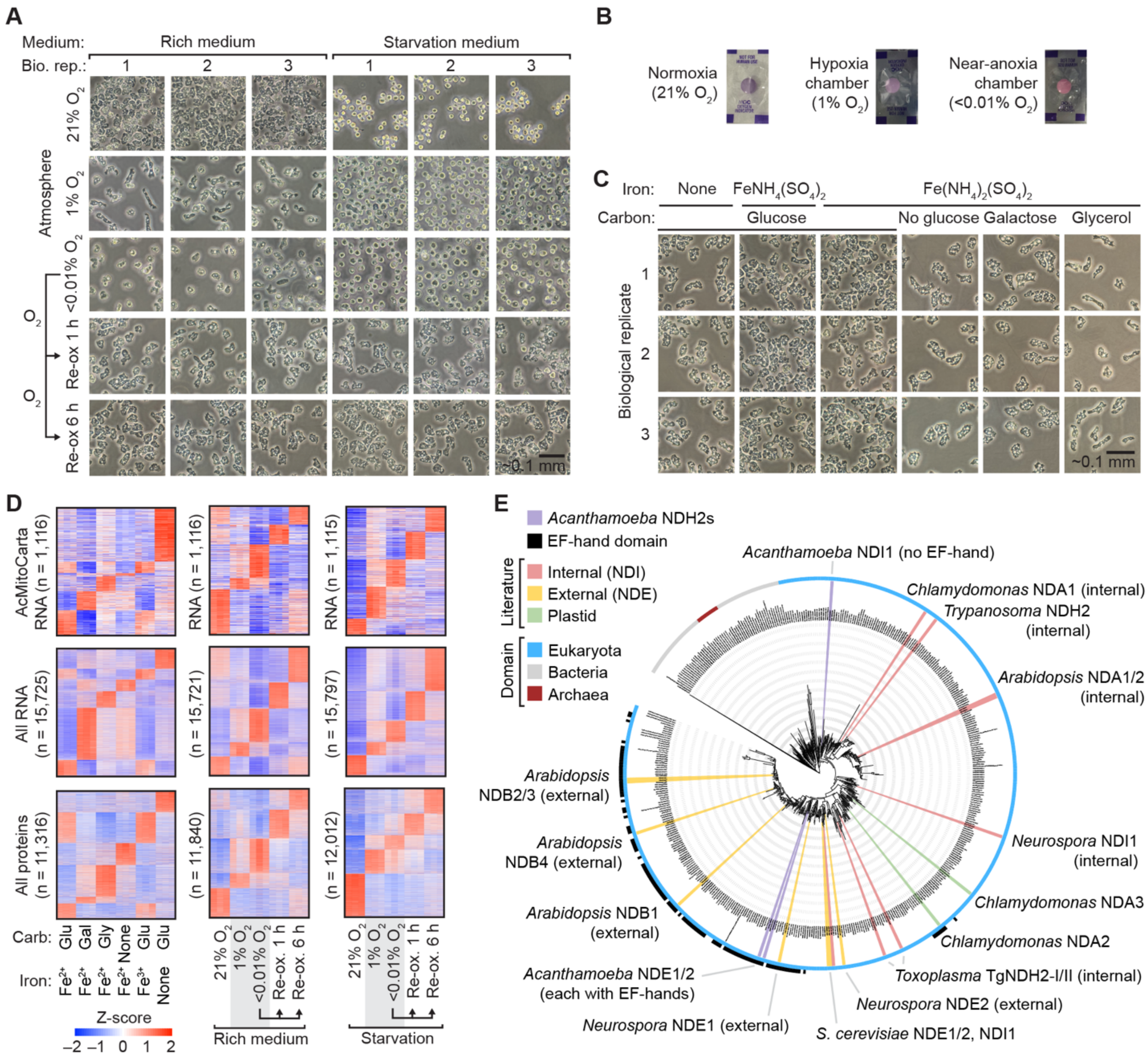

Figure S5. Perturbation profiling microscopy and data analysis, related to Figure 5

(A) Phase contrast microscopy of *Acanthamoeba* cultured for 24 hours under the indicated O_2 atmosphere and in the indicated medium (n = 3 biological replicates). Where indicated, re-oxygenation (re-ox.) was performed for 1 h or 6 h under 21% O_2. Scale bars are estimated to be ~0.1 mm and magnification is approximately equal across the images. Mature cysts are seen in starvation medium under 21% O_2. Non-adherent spheroid morphology was seen in starvation medium under 1% O_2 or <0.01% O_2. Note that one biological replicate set of these images is also shown in the main text **Figure 5**, depicted here again for direct comparison with the other two biological replicates of the set.

(B) O_2 indicator pellets inside the chambers with controlled oxygen level in the O_2 perturbation experiment are shown (pink color of indicator pellet shows lack of O_2).

(C) Phase contrast microscopy of *Acanthamoeba* cultured in media with the indicated carbon and iron composition (n = 3 biological replicates). Scale bars are estimated to be ~0.1 mm and magnification is approximately equal across the images. Proliferation was slower in galactose, glycerol, and "no glucose" conditions. Cells exhibit oblong morphology in glycerol. Changes in vacuolation were also observed across the conditions.

(D) Heatmaps of relative transcript or protein abundances (Z-score) across the indicated perturbation conditions (n = 3 biological replicates). Z-scores were calculated and sorted separately across each of the subsets shown. The number of observed transcripts or proteins in each set is indicated.

(E) Phylogeny of NDH2 homologs. Species domain annotations are shown for eukaryotes (blue), bacteria (gray), and archaea (red). Literature-based annotations are highlighted for characterized NDE (external NDH2) and NDI (internal NDH2) proteins. The three *Acanthamoeba* NDH2s are highlighted and assigned to NDE or NDI names based on this analysis. Of note, in *Acanthamoeba*, both NDE1 and NDE2 have EF-hand domains, while NDI1 does not have an EF-hand.

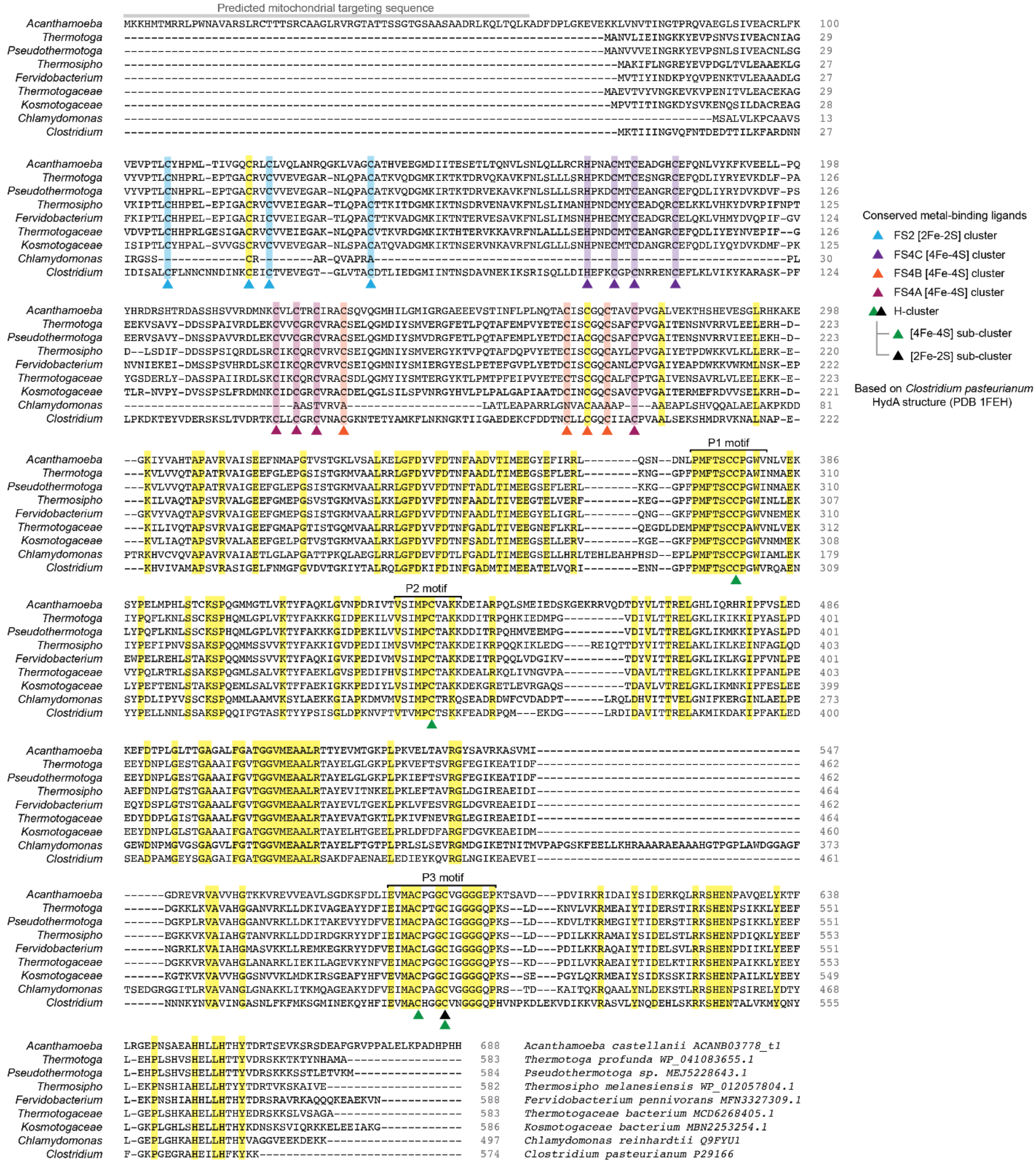

Figure S6. *Acanthamoeba* HydA protein sequence alignment highlighting conserved residues, related to Figure 6
Alignment of *Acanthamoeba* HydA (ACANB03778) protein sequence with those of top BLASTP hits and two structurally-characterized HydA proteins, focusing on *Clostridium pasteurianum* (PDB 1FEH). Residues highlighted in yellow are conserved across all the aligned sequences. Residues noted with triangles are conserved metal ligand binding residues critical for HydA enzyme activity and seen in both *Acanthamoeba* HydA and *Clostridium pasteurianum* HydA.

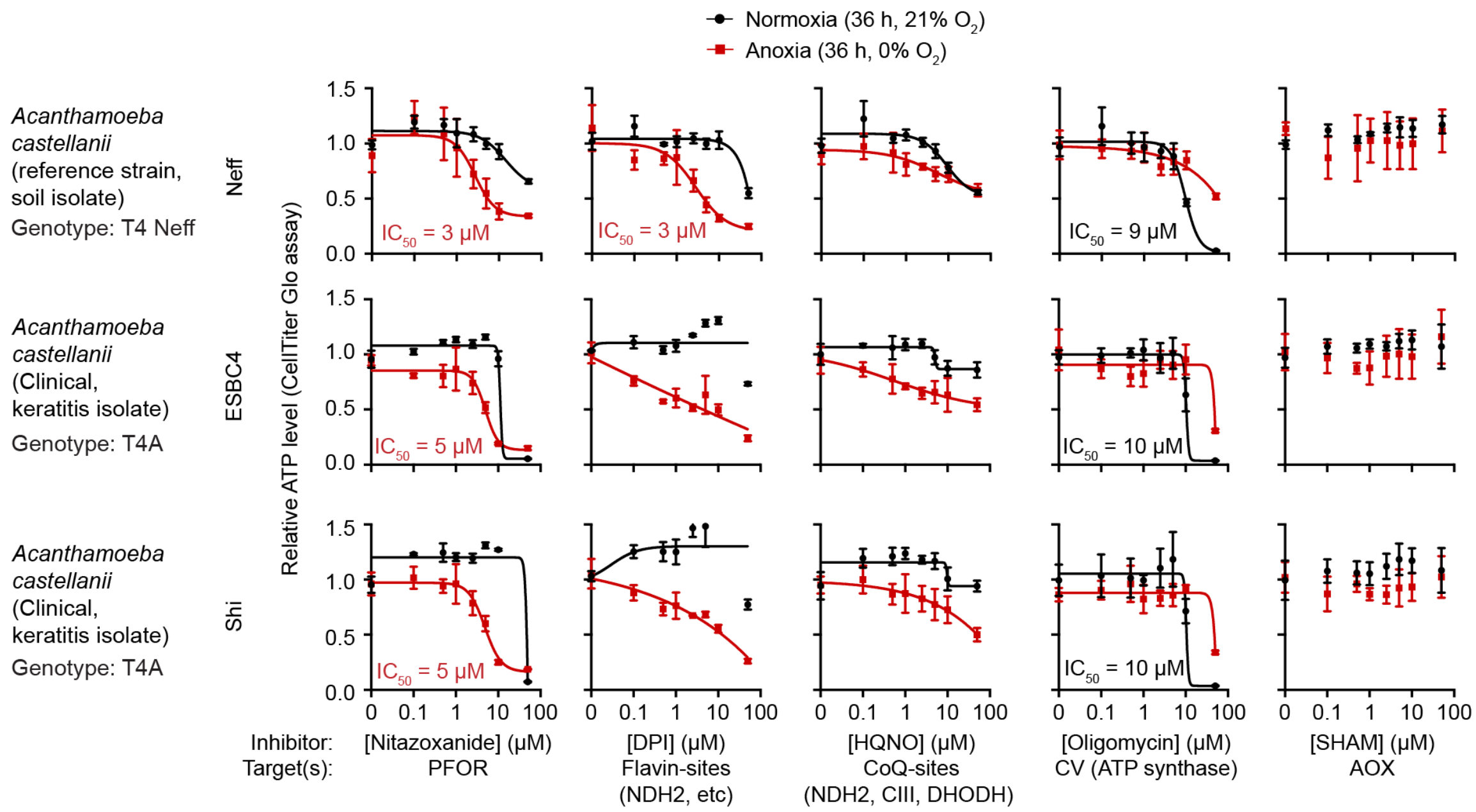

Figure S7. Additional inhibitor dose-response curves for clinical *Acanthamoeba* lines, related to Figure 7
Relative ATP levels (via luciferase based CellTiter Glo assay) in *Acanthamoeba* lines cultured under anoxia or normoxia with the indicated inhibitors (n = 3 biological replicates, error bars indicate standard deviation). IC_{50}s were determined using Prism software nonlinear regression with variable slope (four parameters).

References for Supplemental Information

1. Gawryluk, R.M.R., Chisholm, K.A., Pinto, D.M., and Gray, M.W. (2014). Compositional complexity of the mitochondrial proteome of a unicellular eukaryote (Acanthamoeba castellanii, supergroup Amoebozoa) rivals that of animals, fungi, and plants. J. Proteomics *109*, 400–416. https://doi.org/10.1016/J.JPROT.2014.07.005.

2. Kobayashi, T., Takahara, M., Miyagishima, S.Y., Kuroiwa, H., Sasaki, N., Ohta, N., Matsuzaki, M., and Kuroiwa, T. (2002). Detection and Localization of a Chloroplast-Encoded HU-Like Protein That Organizes Chloroplast Nucleoids. Plant Cell *14*, 1579. https://doi.org/10.1105/TPC.002717.

3. Karcher, D., Köster, D., Schadach, A., Klevesath, A., and Bock, R. (2009). The Chlamydomonas Chloroplast HLP Protein Is Required for Nucleoid Organization and Genome Maintenance. Mol. Plant *2*, 1223–1232. https://doi.org/10.1093/MP/SSP083.

4. Reiff, S.B., Vaishnava, S., and Striepena, B. (2012). The HU protein is important for apicoplast genome maintenance and inheritance in Toxoplasma gondii. Eukaryot. Cell *11*, 905–915. https://doi.org/10.1128/EC.00029-12.

5. Burroughs, A.M., Kaur, G., Zhang, D., and Aravind, L. (2017). Novel clades of the HU/IHF superfamily point to unexpected roles in the eukaryotic centrosome, chromosome partitioning, and biologic conflicts. Cell Cycle *16*, 1093–1103. https://doi.org/10.1080/15384101.2017.1315494.

6. Firat-Karalar, E.N., Sante, J., Elliott, S., and Stearns, T. (2014). Proteomic analysis of mammalian sperm cells identifies new components of the centrosome. J. Cell Sci. *127*, 4128–4133. https://doi.org/10.1242/JCS.157008.

7. Hamel, V., Steib, E., Hamelin, R., Armand, F., Borgers, S., Flückiger, I., Busso, C., Olieric, N., Sorzano, C.O.S., Steinmetz, M.O., et al. (2017). Identification of Chlamydomonas Central Core Centriolar Proteins Reveals a Role for Human WDR90 in Ciliogenesis. Current Biology *27*, 2486-2498.e6. https://doi.org/10.1016/j.cub.2017.07.011.

8. Abramson, J., Adler, J., Dunger, J., Evans, R., Green, T., Pritzel, A., Ronneberger, O., Willmore, L., Ballard, A.J., Bambrick, J., et al. (2024). Accurate structure prediction of biomolecular interactions with AlphaFold 3. Nature 2024 630:8016 *630*, 493–500. https://doi.org/10.1038/s41586-024-07487-w.

9. Baughman, J.M., Perocchi, F., Girgis, H.S., Plovanich, M., Belcher-Timme, C.A., Sancak, Y., Bao, X.R., Strittmatter, L., Goldberger, O., Bogorad, R.L., et al. (2011). Integrative genomics identifies MCU as an essential component of the mitochondrial calcium uniporter. Nature 2011 476:7360 *476*, 341–345. https://doi.org/10.1038/nature10234.
